# Supplementary material for: Genome-wide association meta-analysis of spontaneous coronary artery dissection identifies risk variants and genes related to artery integrity and tissue-mediated coagulation
Source: Nat Genet. 2023 May 29;55(6):964–72. doi: 10.1038/s41588-023-01410-1 (PMC10260398; doi:10.1038/s41588-023-01410-1)
Supplement: Supplementary file 1 — Supplementary Note, Figs. 1–11, Tables 1–17 and consortium membership lists. [file 41588_2023_1410_MOESM1_ESM.pdf]

nature genetics

Article

https://doi.org/10.1038/s41588-023-01410-1

Genome-wide association meta-analysis of spontaneous coronary artery dissection identifies risk variants and genes related to artery integrity and tissue-mediated coagulation

In the format provided by the authors and unedited

Supplementary information

Table of contents

Supplementary note
- Cohorts specific clinical and genetic analyses methods
- Studies specific funding and acknowledgements
- References

Supplementary Figures
- Supplementary Figure 1: GWAS meta-analysis design.
- Supplementary Figure 2. Quantile-quantile plot representation of SNP-based association analysis in individual studies.
- Supplementary Figure 3. Newly identified SCAD genetic risk loci.
- Supplementary Figure 4. Conditional analyses to illustrate 2 independent association signals on the *COL4A1/COL4A2* locus.
- Supplementary Figure 5. Representation of SCAD SNPs enrichment open chromatin regions of 73 tissue with available H3K27ac ChIP-Seq experiments in ENCODE database.
- Supplementary Figure 6. Enrichment of SCAD SNPs in open chromatin regions from human adult cells.
- Supplementary Figure 7. SCAD associated variants overlapping with chromatin marks indicating potential regulatory regions.
- Supplementary Figure 8. Integration of SCAD genetic association with expression of potential target genes in relevant tissues.
- Supplementary Figure 9. Bayesian networks constructed using gene expression of top candidate genes in SCAD risk loci.
- Supplementary Figure 10. Association of lead SNPs with SCAD according to FMD status.
- Supplementary Figure 11. Forest plot representing the genetic correlation between SCAD and CAD unadjusted and after mtCOJO conditioning on cardiometabolic traits.

Supplementary Tables
- Supplementary Table 1: Clinical characteristics of the study populations
- Supplementary Table 2 : Lead associated variants at genome-wide significant SCAD loci in each study and meta-analysis.
- Supplementary Table 3: SNP heritability estimates
- Supplementary Table 4: SNP heritability of the top loci region (1 Mb)
- Supplementary Table 5: Annotation of candidate functional SNPs
- Supplementary Table 6: Colocalization of association with SCAD and eQTL association.
- Supplementary Table 7: Transcriptome-wide associated genes with SCAD in 3 artery tissues (GTEx v7 gene expression models).
- Supplementary Table 8: Druggable genome lookup
- Supplementary Table 9: Colocalization of association with SCAD and five other traits at SCAD top loci
- Supplementary Table 10: Genetic correlation between SCAD and cardiovascular and neurovascular diseases and trait
- Supplementary Table 11: Comparison of SCAD meta-analysis results, unstratified and stratified on FMD status
- Supplementary Table 12: Genetic correlation between SCAD and CAD conditioned on cardiovascular risk factors and blood traits (mtCOJO)
- Supplementary Table 13: Mendelian randomization (MR) analysis between SCAD or CAD and cardiovascular risk factors and blood traits
- Supplementary Table 14: Mendelian randomization (MR) analysis between SCAD or CAD and cardiovascular risk factors and blood traits stratified on sex
- Supplementary Table 15: Details of genotyping, pre-imputation and post-imputation quality control steps per study.
- Supplementary Table 16: Epigenomic datasets used for annotations of SCAD associated variants.
- Supplementary Table 17: details of summary statistics used in to calculate the genetic correlation with SCAD

Consortium Authors
- DISCO Investigators
- International Stroke Genetics Consortium (ISGC) Intracranial Aneurysm Working Group
- MEGASTROKE CONSORTIUM
- CARDIoGRAMplusC4D Consortium

Supplementary note

Cohorts specific clinical and genetic analyses methods

DISCO-3C case control study

DISCO study was registered under Clinical Trials ID: NCT02799186, and approved by regional committee CPP (*comité de protection des personnes*) Sud-Est 6 2016 AU-1258. 3C study protocol was approved by «*comité consultatif de protection des personnes dans la recherche biomédicale*» Bicêtre Hôpital Bicêtre n°99-28 CCPPRB approved 10/06/99, 11/03/2003 and 17/03/2006.

Data from a national French registry of SCAD cases (51 cardiology centres) were analysed prospectively and retrospectively. All subjects 18 years old and above gave their written, informed consent to participate in the study. Exclusion criteria included in custody or guardianship, patients with iatrogenic or traumatic dissection or atherosclerotic dissection. SCAD was identified as an acute or chronic coronary syndrome defined according to universal recommendations with angiographic signs suggestive of SCAD[1] on the initial coronary angiography. In context of MINOCA, a pathological cardiac magnetic resonance (CMR) could lead to the diagnosis of SCAD retrospectively by rereading the angiograms. In case of ambiguity, intravascular ultrasound (IVUS), optical coherence tomography (OCT) or repeat coronary angiography was performed at the discretion of the operator to confirm the diagnosis of SCAD. All SCAD cases were subsequently confirmed by a core lab of 3 operators (NC, PM, GS) experienced in the field of SCAD and intra coronary imaging. Coronary segmentation was defined according to the AHA classification[2]. Screening for FMD was done at the discretion of the different centres either by CT scan, magnetic resonance, angiography or Doppler in cervico-cephalic, visceral and iliac arteries. The diagnosis of FMD was considered confirmed if at least one extra-cardiac location was verified by an experienced radiologist (LC) according to established criteria [3]. Complete screening was defined from brain to pelvis.

Blood samples were taken at the time of inclusion (during hospitalization for the prospective cohort or remotely in the retrospective cohort). DNAs was extracted from whole blood using a ChemagicTM 360 device (Perkin Elmer). Genotyping quality control was performed using PLINK v 2.022[4]. We checked for sex mismatches, first degree relatedness and ancestry estimation. Genotyping data were filtered using the criteria of Hardy-Weinberg equilibrium exact test p value smaller than 0.001 (--hwe 0.001, default value in PLINK 2.0) and missingness per marker >1% (--geno 0.01). The minimum allele frequency was set to 0.01 (--maf 0.01). Data was imputed using minimac4 incorporated in a cloud-based imputation server using HRC r1.1 2016 reference panel[5].

The Three-City Study (3C Study) is a population-based longitudinal study of the relation between vascular diseases and dementia in persons aged 65 years and older[6]. Participants were recruited from three French cities: Bordeaux (South-West), Dijon (North-East) and Montpellier (South-East). The 3C Study extended from 1999 to 2012. Participants underwent regular extensive examination. Examination included measurements of traditional vascular risk factors (blood pressure, glycaemia, lipids, etc.), cognitive functions, and subclinical vascular diseases using carotid ultrasound and cerebral magnetic-resonance imaging (MRI). DNA collection and genotyping were described previously[7].

SCAD-UK case-control studies

The UK SCAD study (ISRCTN42661582) was approved by the UK National Research Ethics Service (14/EM/0056) and the UK Health Research Authority. All patients gave written informed consent to participate in the study. Patients with SCAD were ascertained either by clinician referral to the UK national SCAD clinical service or by self-referral to an online portal. Only patients with SCAD confirmed on invasive angiography were included. Patients with

atherosclerotic, iatrogenic or traumatic coronary dissections were excluded. Demographic data was obtained from the patients' medical record and from the patient through an online survey and direct contact as required. Hypertension was defined as being on treatment prior to the first clinical SCAD event. Gestational hypertension or diabetes were not included. All angiograms were jointly reported by at least 2 SCAD-experienced interventional cardiologists. Cross sectional imaging by MRA or CTA either undertaken on research or clinical grounds were used to determine the presence or absence of extra-coronary SCAD-associated arteriopathies. Those with FMD, aneurysms or dissections were considered abnormal. Those without were considered normal. Tortuosity or focal stenotic disease were not considered abnormal in the absence of these other features.

Sequencing of SCAD cases for arm I of UK SCAD study was described previously [8]. Genomic DNA from SCAD cases was extracted and underwent paired-end 150bp WGS at Human Longevity Inc using the NovaSeq6000 platform. For SCAD cases, >98% of consensus coding sequence release 22 (CCDS) has at least 10x coverage and average coverage of the CCDS achieved 42-fold read-depth. Variants were lifted over to genome assembly GRCh37 using NCBI remap and filtered to maf > 0.01 and hwe > 1e-6 using plink 1.9. Variant identifiers were updated to UK biobank formatting and individual sample files merged. SCAD-UK II case genomic DNA were genome-wide genotyped on the Illumina-Infinium™ Global Screening Array-24 v2.0 +MD array (665,608 markers) at the Spanish National Cancer Research Centre (CNIO), in the Human Genotyping lab, a member of National Genotyping Center (CeGen) [9], whereas genotyping of the SCAD-UK II control genomic DNA were genome-wide genotyped at Applied Biosystems™ Axiom™ Genotyping Services (Thermo Fisher Scientific) using the Applied Biosystems™ Axiom™ UK Biobank WCSG Genotyping Array (825,928 markers), as part of the UK Biobank genotyping project[10]. SCAD-UK II cases and controls were pre-imputation QC filtered using PLINK v1.90b6.14. Variants were excluded if the SNP call rate (CR) < 95% for minor allele frequency (maf) > 0.05 and CR < 98% for maf ≤ 0.05 for all chromosomes 1-22, X (except for cases in the X chromosome, where due to a poorer call rate we excluded variants only if the SNP CR was < 90% for maf > 0.05 and CR < 95% for maf ≤ 0.05), maf < 0.005, hwe deviation was P < 1 x 10-06. Samples were removed if there was a sex-mismatch between reported and genetically inferred sex or have an individual CR < 95% after SNP QC. Further samples were removed if relatedness using a PLINK PI-HAT was > 0.1875, or if samples had high heterozygosity, deviating from the mean sample heterozygosity rate (above ± 3 standard deviations). Will Rayner's HRC checking tool was used to assess and correct PLINK files for strand and REF/ALT orientation of alleles, using the Haplotype Reference Consortium release 1.1 (HRC r1.1) reference panel[11]. SCAD-UK II cases and controls were separately imputed on the Michigan Imputation Server with validated vcf.gz input files for each chromosome (1-22, X) with eagle (2.4) for pre-phasing and minimac4 (1.5.7) for imputation using either the Haplotype Reference Consortium release 1.1 (HRC r1.1) reference panel or the 1000g-phase-3-v5 (https://www.internationalgenome.org/) reference panel. Post imputation the two reference panels were combined using QCTOOLv2 (https://www.well.ox.ac.uk/~gav/qctool_v2/). To combine the two reference panels the 1000g-phase-3-v5 imputation variant dataset were merged onto the HRC r1.1 imputation variant dataset, so that only variants in addition to the HRC imputation were added. Subsequently, the cases and controls were combined again using using QCTOOLv2. Post-imputation QC excluded all SNPs with an imputation R-square < 0.3, hwe deviation of P < 1 x 10-06 in cases or P < 1 x 10-04 in controls, a minor allele count < 20 or maf < 0.01 in cases.

VCCRI case control studies

The study was approved by the St. Vincent's Hospital Human Research Ethics Committee (HREC/16/SVH/338, protocol number SVH 16/245) and conducted in accordance with the

Australian National Health and Medical Research Council's National Statement on Ethical Conduct in Human Research and the CPMP/ICH Note for Guidance on Good Clinical Practice. Arm II control sample study was approved by the St. Vincent's Hospital Human Research Ethics Committee (HREC/17/SVH/315) and conducted in accordance with the Australian National Health and Medical Research Council's National Statement on Ethical Conduct in Human Research and the CPMP/ICH Note for Guidance on Good Clinical Practice. Ethics approval number 2015.028 for samples from Royal Melbourne Hospital, Melbourne, Victoria, Australia.

SCAD cases were recruited via a social media platform or through direct referral from cardiologists. SCAD diagnosis was confirmed by review of coronary angiogram images by an expert interventional cardiologist (DM) blinded to the results of the genetic analysis. FMD diagnosis or negative scan was self-reported by subjects. Screening was partial in some cases. Subjects were considered to have hypertension if they were prescribed blood pressure lowering drugs before the SCAD occurred. All subjects were asked the clinical diagnostic criteria for migraines. Control samples for arm I of VCCRI study are from the MRGB cohort[12]. For the arm II of VCCRI study, control samples were unrelated atrial fibrillation cases and unrelated dilated cardiomyopathy patients and unrelated unaffected family members.

For VCCRI I, genomic DNA was extracted (PureLink GenomicDNA Mini Kit; Invitrogen) from buccal cells collected using a cheek swab. WGS was performed using paired-end KAPA PCR-Free v2.1 libraries on the Illumina HiSeq X Ten platform with 30x coverage (Kinghorn Centre for Clinical Genomics, Sydney). Bioinformatic processing was based on that used for the MGRB cohort[12]. Reads were aligned to the GRCh37 reference genome with Burrows-Wheeler Aligner[13] and SNVs and INDELs were called with the Genome Analysis Toolkit Best Practices pipeline[14]. All variants were annotated with Annovar[15] against RefSeq (version 01-06-2017). Principal component analysis using 17,453 SNVs and projection to the 1000 Genomes principal components were used to confirm ethnicity (akt v0.3.2). Non-relatedness of subjects in the cohort was confirmed with akt v0.3.2. Genotypes were considered missing if any of the following criteria were met: $GQ < 30$; $DP < 10$; $DP > 150$; heterozygous genotype with $AB < 0.25$ or $AB > 0.75$.

For VCCRI II, genomic DNA for cases was extracted from buccal cells collected using buccal swabs or mouthwash. Scope brand mouthwash was used for DNA collection. DNA was extracted using Purelink® Genomic Kit, Invitrogen, Australia for buccal swabs and Puregene Core Kit A, Qiagen, USA for mouthwash. Control DNA extraction methods were described previously[16] (AF and DCM control samples). Samples were genotyped on the Axiom UK Biobank array (Kinghorn Centre for Clinical Genomics, Sydney). Genotyped case and control data were imputed using the MIS using the HRCr1.1 reference panel. Multiallelic SNPs and variants with gnomAD genome NFE <= 0.0001 were excluded. Non-relatedness of subjects in the cohort was confirmed with KING v2.1.4.

Mayo Clinic case control study

Study participants provided written informed consent under clinical and genetic research protocols approved by the Mayo Clinic Institutional Review Board (NCT01429727; NCT01427179).

Subjects were recruited from the Mayo Clinic patient population, including local residents, self- and physician-referred patients, and individuals who contacted investigators via the study website (www.mayo.edu/research/SCAD), and social media. Study subjects were consecutively enrolled in the Mayo Clinic SCAD registry after diagnostic confirmation of SCAD by review of coronary angiograms by an experienced interventional cardiologist. Demographic and clinical data were abstracted from questionnaires and medical records. A majority of individuals (95%) lived in the USA. FMD screening was performed in a subset of

subjects by computed tomography angiography imaging of at least two arterial beds from brain to pelvis.
Whole blood or saliva samples were obtained for genomic DNA extraction. Cases were genotyped for 713,599 SNPs by the Mayo Clinic Medical Genome Facility Genotyping Core using the Infinium OmniExpress-24 v1.2 BeadChip array (Illumina). Raw data were compiled using GenomeStudio software (Illumina) and exported to PLINK v1.9 for quality control (QC) and genome association analysis. Control genotypes were extracted from existing data files in the Mayo Genome Consortia, Center for Individualized Medicine, generated from Infinium HumanHap550, 610, 660 or OmniExpress BeadChip arrays (Illumina). QC filters excluded samples with a call rate <95%, sex-discordance, duplicates or cryptic evidence of relatedness using pairwise identical by descent estimates. To mitigate false-positive associations caused by population stratification, samples from individuals of non-Caucasian ancestry (<90% Caucasian) were detected using STRUCTURE software and excluded. Principal component analysis was used to examine the data for clustering due to platform and race. Exclusion criteria for genotyped SNPs were a call rate <95%, minor allele frequency <0.01, or deviation from Hardy-Weinberg equilibrium ($P < 1\times10^{-5}$). Following initial QC assessment, imputation was conducted separately for each platform, then combined to harmonize data. Imputation was performed on the University of Michigan Imputation Server using the Haplotype Reference Consortium reference panel. Imputed genotypes with a dosage r^2 of at least 0.7 and a minor allele frequency of at least 0.01 were used for analysis. Genome-wide association analysis of SNPs and SCAD risk were evaluated using logistic regression models assuming additive allele effects. To control for population stratification, the first 5 principal components were used as covariates in the models. Strength of association was estimated by calculating the odds ratio (OR) and corresponding 95% confidence interval (CI).

CanSCAD/MGI (UBC/MGI) case control study
Research ethics board approvals were obtained at each site of SCAD patient inclusion, and all patients provided informed consent for participation. GenSCAD study was approved by IRB approval: HUM00113268, SCAD Registry. Research ethics board approvals were obtained (IRB approval: HUM00112101), genetic analysis of arterial dysplasia and remodeling (MGI/AOS), and all patients provided informed consent for participation.
The CanSCAD genetic substudy (N=502) included SCAD patients from the prospective Canadian SCAD Cohort Study and the Non-Atherosclerotic Coronary Artery Disease (NACAD) Study. Patients presenting with acute SCAD were prospectively enrolled from 22 sites throughout North America (20 sites in Canada and 2 in the United States). SCAD diagnosis was confirmed on coronary angiography by the UBC core laboratory research team. Detailed baseline demographics, targeted history for predisposing conditions and precipitating stressors, and laboratory screening for predisposing conditions were performed. Screening for FMD was recommended for all SCAD patients, and multifocal FMD was defined according to consensus guidelines[3]. Patients were prospectively followed post-discharge at 1, 6, and 12 months, and annually thereafter for 3 years for cardiovascular (CV) events. Genetic studies were performed on CanSCAD patients who provided informed consent. Collection of DNA was obtained through blood or saliva self-collection kit (Oragene-500 kit, DNAGenotek). DNA was extracted according to the manufacturer's instruction (DNAGenotek) as previously described, and quantified using the Quant-iT PicoGreen assay (Life Technologies). DNA samples were normalized to a concentration of 50ng/µl for genotyping. The processed DNA samples were batched and transferred to the University of Michigan for GWAS analysis.
Due to the possibility of duplicate enrollment of individuals residing in the U.S. into multiple studies, particularly the Mayo clinic study which enrolled remote subjects from other institutions, this was accounted for in the current meta-analysis by removal of individuals from

U.S. sites. This led to the removal of 49 individuals from the CanSCAD resource for the current analysis. By restricting to non-Finnish European individuals, 357 individuals were included in the final analysis.

The Michigan Genomics Initiative (MGI) is a program that recruited participants while awaiting diagnostic, interventional, and surgical procedures. Participants provided a blood sample for genetic analysis and agreed to link their sample to their electronic health record and other sources of health information. The current study's analyses involved 13,756 individuals from MGI genotyped with the same version (v1.1) of the Illumina BeadArray genotyping platform as the CanSCAD study SCAD cases at the University of Michigan DNA Sequencing Core Facility. Several ICD codes corresponding to diagnoses of as arterial diseases and connective tissue disorders were excluded. Controls were matched by age, sex, and ancestry through principal components of the GWAS data.

Genotyping of all CanSCAD, and 13,756 MGI samples, excluding samples with arterial diseases and connective tissue disorders based on ICD codes[17], were conducted by the University of Michigan DNA Sequencing Core using the Illumina Infinium HTS Assay Protocol, a semi-custom Infinium CoreExome-24v1.1 BeadArray with 607,778 SNP markers (UM_HUNT_Biobank_v1-1_20006200_A), and the Illumina GenomeStudio v2011.1. This GWAS+exome chip platform includes standard genome-wide tagging SNPs (N~240,000), exomic variants (n~280,000) and custom content from previously published GWASs, additional exonic variants selected from sequencing studies, ancestry informative variants and Neanderthal variants. Data Analysis Software package with Genotyping Module v1.9.4 and Illumina GenomeStudio (version 2.0) were used to cluster and call genotypes. Sample filtering was performed to exclude samples with call rate < 98%, estimated contamination > 2.5% (BAF regress), chromosomal missingness greater than 5 times other chromosomes, and sex mismatch between genotype-inferred sex and reported gender. Variant filtering was performed to exclude probes that could not be perfectly mapped to the human genome assembly (Genome Reference Consortium Human genome build 37 and revised Cambridge Reference Sequence of the human mitochondrial DNA; BLAT); Hardy-Weinberg equilibrium deviations in European ancestry samples (P<0.00001); variant call rate < 98%. Basic quality control (QC) filters including HWE P<0.000001, and variant missing call rate >2%, were implemented for each of our lab chip data and MGI chip data, before combining the two data sets.

We merged multiple batches of genotype data of SCAD cases and then applied pre-quality control using the HRC Imputation preparation and checking tool by the McCarthy Group before merging them with MGI genotyped data, which applied the same pre-HRC-imputation QC. It compares each of our individual genotyped data with HRC reference, and corrects the variants strand-flip as well as aligns the allele codes with HRC reference alleles. It also removes A/T & G/C SNPs if MAF > 0.4, SNPs with differing alleles, SNPs with > 0.15 allele frequency difference, and SNPs not in reference panel. After this process, 351,487 polymorphic variants remained (chr1-23). The total genotyping rate was 0.99. Based on the genotyping result, we excluded 1 sample with missing call rate >2%, 1 duplicate or close relatives based on whole genome genotyping data identity-by-descent (IBD) >0.35, 2 gender mismatched samples, 2 genetic syndrome cases, and 2 are further confirmed not SCAD cases. There were no samples failing QC due to checks of inbreeding coefficient. For the merged data, we further confirmed that none of the SCAD cases and MGI controls were duplicates or overlapped based on the IBD analysis.

We then imputed autosomal chromosome genotypes of the Haplotype Reference Consortium (HRC) using the Michigan Imputation Server on the 13,756 MGI and all the UBC SCAD samples. The parameters for imputation included: 1) Minimac4 method; 2) HRC r1.1 2016 reference panel; 3) Eagle v2.3 as phase output; 4) EUR as quality control population. We filtered poorly imputed variants (R2<0.8) and rare variants (MAF <1%). We also excluded

SNPs with potential frequency mismatches comparing with reference panel (markers with Chi-squared greater than 300). 6,690,240 imputed variants (chromosomes 1-22) remained after filtering. The correlation r^2 between the ref allele frequency of our samples and the HRC reference panel was 0.999. For the matched control sample selected from 13756 MGI control pool for each case, we required the control to have the same gender, close birth years, and close ancestries as the case. We expected that every case was matched to at least one control. We took an approach that we searched from +/-5 years (5-year window) in age first, followed by +/-10 years instead, and so on to +/-30 years. We stopped searching once there was at least one control selected. From the possible controls in the applicable sex and age category, we chose the best ethnic match for each case that had the smallest principal components distance (via the top 3 PCs computed from the TRACE program). To guarantee every case could match to 6 controls, we repeated the entire procedure 6 times. Furthermore, in our final GWAS, we only focused on non-Finish European subjects, based on the criteria of within mean +/- 6 SD region of PC1 and PC2 from 1000 Genome reference. 2125 matched MGI controls were selected for 357 SCAD case samples. We finally tested the single genetic imputed variants using the PLINK program, with the first five principal components as covariates.

DEFINE-SCAD case control study

The DEFINE study (NCT01967511) protocol is approved by the Human Research Ethics Committee of the Icahn School of Medicine at Mount Sinai[18]. Patients with suspected arteriopathies were seen and assessed in the Mount Sinai Vascular Medicine Clinic. Inclusion criteria for entry into DEFINE included >=18 years of age, being freely willing to participate and fluency in English. FMD cases were required to have a clinical diagnosis of multifocal FMD confirmed by imaging [computed tomographic angiography, magnetic resonance angiography, or catheter-based angiography]. SCAD patients were required to have a clinical diagnosis of SCAD confirmed by invasive coronary angiography. Initially only females were permitted to enter the study, but from early 2017 both males and females were enrolled. For healthy controls, inclusion criteria included no clinical features of FMD, cervical artery dissection, or SCAD (including no cervical or abdominal bruits, an absence of family history of sudden death or aneurysm) and absence of any major ongoing systemic disease including any condition requiring hospitalization, immune suppression, intravenous or injected medications or that result in functional impairment in the performance of activities of daily living. Healthy controls were recruited from the general population and were pre-screened by the same clinical team and matched to study cases according to age, sex, race/ethnicity, and body mass index (BMI). Exclusion criteria (for cases and controls) included: co-morbidities which reduce life expectancy to 1 year; any solid organ or haematological transplantation, or those in whom transplantation is considered; active autoimmune disease; illicit drug use; HIV positive; prior malignancy. In controls, an additional exclusion criterion was an early-onset family history of any form of vascular disease. Healthy controls also underwent screening clinical assessment, with specific attention paid to any history or physical examination findings suggestive of FMD or other vascular disease, by two clinical experts in FMD (J.W.O. and D.K.-D.).

If the entry criteria are met and following informed consent, blood draw and skin punch-biopsy (from the medial upper arm) were performed. At the blood draw, 10mL of blood were collected into ethylenediaminetetraacetic acid (EDTA) tubes and reserved for deoxyribonucleic acid (DNA) extraction. DNA was isolated from whole blood using the Puregene Blood Core kit B (cat# 158467, Qiagen, Germantown, MD, USA). DNA was aliquoted and frozen at -80°C. Of 384 total DNA samples processed, 336 samples were processed using Illumina-Human-Omni-Express-Exome genotyping array and 48 samples were processed using Infinium OmniExpressExome-8v1.6 array, which included additional CAUSE and DEFINE study

subjects not included in this analysis. All samples showed a call rate > 99% and were thus retained. No genetically identical pairs were found, no outliers were identified in a Principal Components Analysis (run using eigenstrat), and the sex of all samples was correctly predicted for each subject. Further inspection of the samples' heterozygosity revealed that there were six subjects who did not pass the QC and were excluded. Genotyping array probes were filtered according to call rate (> 95%) and Hardy-Weinberg equilibrium test P value (> $1x10^{-6}$). Data from Illumina-Human-Omni-Express-Exome genotyping array (336 subjects) and Infinium OmniExpressExome-8v1.6 array (48 subjects) yielded 962,157 shared variants after QC. Genotyping data was imputed using the HRC (haplotype research consortium, http://www.haplotype-reference-consortium.org/) reference and the Michigan Imputation Server, using the MACH/minimac imputation pipeline. We successfully imputed 16,229,543 variants.

Studies specific fundings and acknowledgements

DISCO-3C case control study

The DISCO study was supported by the European Research Council grant (ERC-Stg-ROSALIND-716628), the French Society of Cardiology foundations "Coeur et Recherche" and "La Fédération Française de Cardiologie", and the French Coronary Atheroma and Interventional Cardiology Group (GACI). The French study acknowledge the Spanish National Cancer Research Centre, in the Human Genotyping lab, a member of CeGen Biomolecular resources platform (PRB3), to be supported by grant PT17 /0019, of the PE I+D+i 2013-2016, funded by Instituto de Salud Carlos III and a European regional development fund (ERDF). The genotyping of the controls from the Three-City Study (3C) was supported by the non-profit organization Fondation Alzheimer (Paris, France) to PA. We acknowledge all clinicians and patients who contributed to the DISCO register, the French Society of Cardiology and the French Coronary Atheroma and Interventional Cardiology Group for their support, the Clinical Research Associates of the Clermont-Ferrand University Hospital: Elodie Chazot, Carole Bellanger, Laurie Cubizolles, Aurélie Thalamy, Ouarda Lamallem. We acknowledge the Spanish National Cancer Research Centre, in the Human Genotyping lab, a member of CeGen where genotyping was performed for DISCO patients and the European Global Screening Array Consortium.

SCAD-UK case-control studies

We thank AstraZeneca's Centre for Genomics Research, Discovery Sciences, BioPharmaceuticals R&D for funding the sequencing of and providing the bioinformatics support related to subjects in cohort SCAD-UK I. The study is supported by the British Heart Foundation (BHF) PG/13/96/30608, the National Institute for Health Research (NIHR) rare disease translational collaboration, the Leicester NIHR Biomedical Research Centre and BeatSCAD. Dr Webb is funded by the British Heart Foundation (SP/16/4/32697). We are grateful for the support of SCAD-survivors and our clinical colleagues throughout the UK and the leadership of the ESC-ACCA SCAD Study Group. We specifically acknowledge the support of Jenny Middleton, Jane Plume, Donna Alexander, Daniel Lawday and Andrea Marshall for all their support for SCAD research. We thank the AstraZeneca Centre for Genomics Research Analytics and Informatics team for processing and analysis of sequencing data.

VCCRI case control studies

The VCCRI study was supported by Cardiac Society of Australia and New Zealand (CSANZ) Cardiovascular Research Innovation Grant (RMG, DWMM, DF, JCK), National Health and Medical Research Council (NHMRC), Australia (APP1161200), NSW Health Early Mid-

Career Cardiovascular Grant (EG), NSW Health Senior Clinician Cardiovascular Grant (RMG). The authors would like to thank the SCAD patients for their enthusiastic participation in this study, Sarah Ford and Pamela McKenzie and SCAD Research Inc, our colleagues who referred SCAD cases, and the Medical Genome Reference Bank including the 45 and Up and ASPREE study patients who were controls for this study. The authors would like to thank Claire MY Wong, Ketan Mishra, and Renee Johnson for their contributions to data collection and sample processing.

Mayo Clinic case control study
The Mayo Clinic study was supported by funding from SCAD Research Inc, National Institutes of Health (NIH: T32 GM72474), and resources from the Genome Consortia, Mayo Clinic Center for Individualized Medicine. The authors thank our clinical colleagues who referred SCAD cases and the patients who supported this research.

CanSCAD/MGI (UBC/MGI) case control study
The U.S. site of the CanSCAD/MGI study funding support included grants from National Institutes of Health (R01HL139672, R35HL161016), Heart and Stroke Foundation of Canada (G-17-0016340), Canadian Institutes of Health Research (grant #136799), and the University of Michigan Frankel Cardiovascular Center and M-BRISC program. S.K.G. is supported by R35HL161016, R01HL086694, Department of Defense, and the A. Alfred Taubman Institute. J.S. and L.R.B are supported by a Michael Smith Foundation for Health Research Scholar award. L.R.B is a Canada Research Chair in Precision Cardiovascular Disease Prevention. The University of Michigan Advanced Genomics Core performed genotyping of all CanSCAD and MGI samples. The authors acknowledge the University of Michigan Precision Health Initiative and Medical School Central Biorepository for providing biospecimen storage, management, processing and distribution services and the Center for Statistical Genetics in the Department of Biostatistics at the School of Public Health for genotype data management in support of this research. We thank all the participants of the studies included in the analyses. We acknowledge the FMD Society of America and Vancouver SCAD Conference organizers for enabling study enrolments at patient meetings.

DEFINE-SCAD case control study
DEFINE is supported by the US National Institutes of Health (R01HL148167) and we acknowledge the many philanthropic supporters of DEFINE who from the outset have enabled and facilitated this study. JCK acknowledges research funding from, New South Wales health grant RG194194, the Bourne Foundation and Agilent.
We acknowledge and thank all the subjects, both cases and controls, in the DEFINE study. We also thank all our clinical colleagues who referred SCAD, FMD and CeAD patients to this study.

Xia Yang lab
We acknowledge funding from the NIH/NHLBI R01HL147883 for XY, and American Heart Association Predoctoral Fellowship 829009 and UCLA Integrative Biology and Physiology Edith Hyde Fellowship for MB.

References

1 Motreff, P. *et al.* How and when to suspect spontaneous coronary artery dissection: novel insights from a single-centre series on prevalence and angiographic appearance. *EuroIntervention* **12**, e2236-e2243 (2017). https://doi.org:10.4244/EIJ-D-16-00187

2 Austen, W. G. *et al.* A reporting system on patients evaluated for coronary artery disease. Report of the Ad Hoc Committee for Grading of Coronary Artery Disease, Council on Cardiovascular Surgery, American Heart Association. *Circulation* **51**, 5-40 (1975). https://doi.org:10.1161/01.cir.51.4.5

3 Gornik, H. L. *et al.* First International Consensus on the diagnosis and management of fibromuscular dysplasia. *Vasc Med* **24**, 164-189 (2019). https://doi.org:10.1177/1358863X18821816

4 Chang, C. C. *et al.* Second-generation PLINK: rising to the challenge of larger and richer datasets. *GigaScience* **4**, 7 (2015). https://doi.org:10.1186/s13742-015-0047-8

5 Das, S. *et al.* Next-generation genotype imputation service and methods. *Nature genetics* **48**, 1284-1287 (2016). https://doi.org:10.1038/ng.3656

6 Vascular factors and risk of dementia: design of the Three-City Study and baseline characteristics of the study population. *Neuroepidemiology* **22**, 316-325 (2003). https://doi.org:10.1159/000072920

7 Duperron, M. G. *et al.* Burden of Dilated Perivascular Spaces, an Emerging Marker of Cerebral Small Vessel Disease, Is Highly Heritable. *Stroke* **49**, 282-287 (2018). https://doi.org:10.1161/strokeaha.117.019309

8 Carss, K. J. *et al.* Spontaneous Coronary Artery Dissection: Insights on Rare Genetic Variation From Genome Sequencing. *Circ Genom Precis Med* **13**, e003030 (2020). https://doi.org:10.1161/CIRCGEN.120.003030

9 Georges, A. *et al.* Genetic investigation of fibromuscular dysplasia identifies risk loci and shared genetics with common cardiovascular diseases. *Nat Commun* **12**, 6031 (2021). https://doi.org:10.1038/s41467-021-26174-2

10 Bycroft, C. *et al.* The UK Biobank resource with deep phenotyping and genomic data. *Nature* **562**, 203-209 (2018). https://doi.org:10.1038/s41586-018-0579-z

11 McCarthy, S. *et al.* A reference panel of 64,976 haplotypes for genotype imputation. *Nature genetics* **48**, 1279-1283 (2016). https://doi.org:10.1038/ng.3643

12 Pinese, M. *et al.* The Medical Genome Reference Bank contains whole genome and phenotype data of 2570 healthy elderly. *Nat Commun* **11**, 435 (2020). https://doi.org:10.1038/s41467-019-14079-0

13 Li, H. & Durbin, R. Fast and accurate short read alignment with Burrows-Wheeler transform. *Bioinformatics* **25**, 1754-1760 (2009). https://doi.org:10.1093/bioinformatics/btp324

14 Van der Auwera, G. A. *et al.* From FastQ data to high confidence variant calls: the Genome Analysis Toolkit best practices pipeline. *Curr Protoc Bioinformatics* **43**, 11.10.11-11.10.33 (2013). https://doi.org:10.1002/0471250953.bi1110s43

15 Wang, K., Li, M. & Hakonarson, H. ANNOVAR: functional annotation of genetic variants from high-throughput sequencing data. *Nucleic Acids Res* **38**, e164 (2010). https://doi.org:10.1093/nar/gkq603

16 Horvat, C. *et al.* A gene-centric strategy for identifying disease-causing rare variants in dilated cardiomyopathy. *Genet Med* **21**, 133-143 (2019). https://doi.org:10.1038/s41436-018-0036-2

17 Saw, J. *et al.* Chromosome 1q21.2 and additional loci influence risk of spontaneous coronary artery dissection and myocardial infarction. *Nat Commun* **11**, 4432 (2020). https://doi.org:10.1038/s41467-020-17558-x

18 Olin, J. W. *et al.* A Plasma Proteogenomic Signature for Fibromuscular Dysplasia. *Cardiovascular research* (2019). https://doi.org:10.1093/cvr/cvz219
19 Zhang, K. *et al.* A single-cell atlas of chromatin accessibility in the human genome. *Cell* **184**, 5985-6001 e5919 (2021). https://doi.org:10.1016/j.cell.2021.10.024

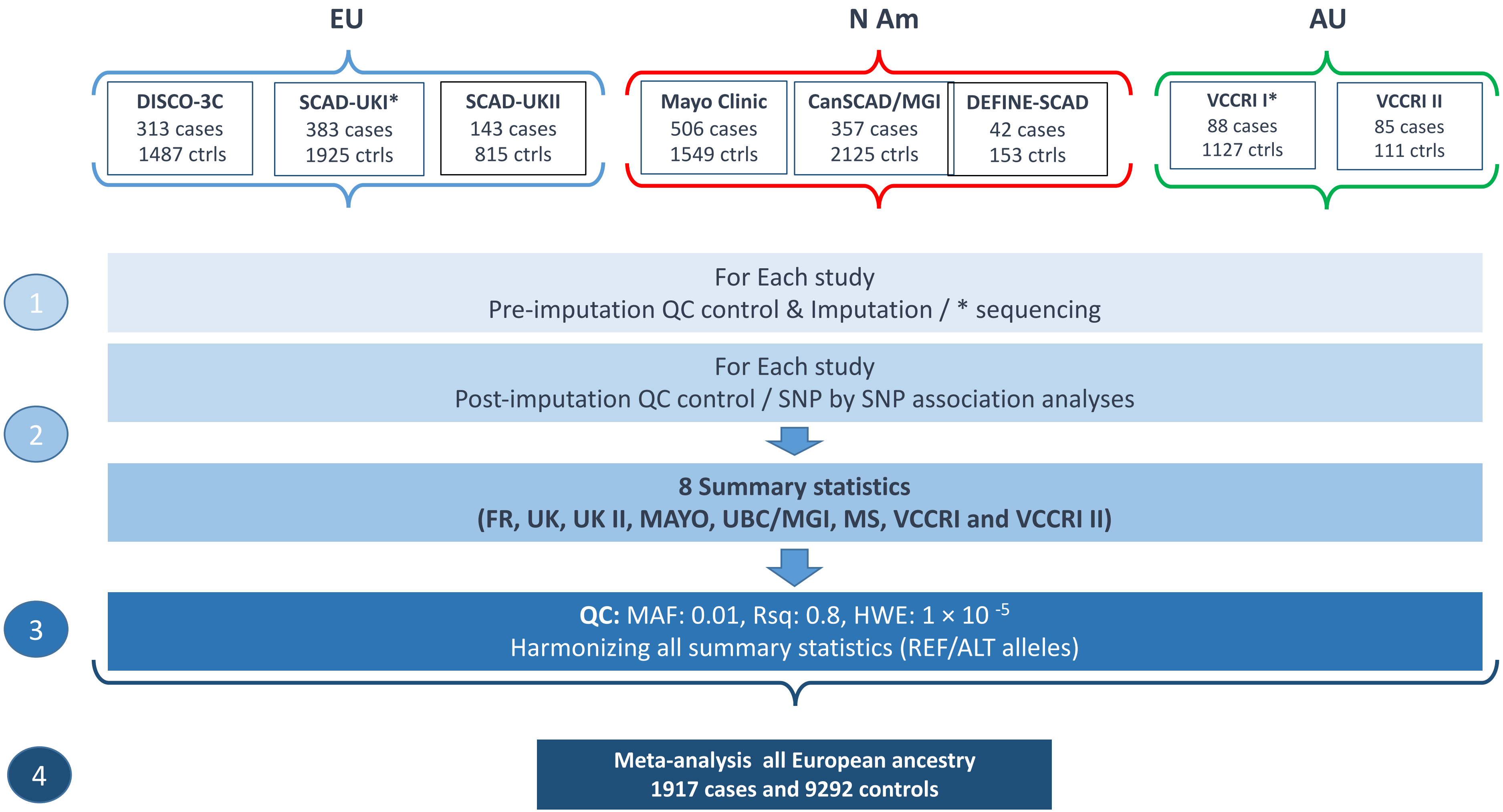

Supplementary Figure 1: GWAS meta-analysis design.

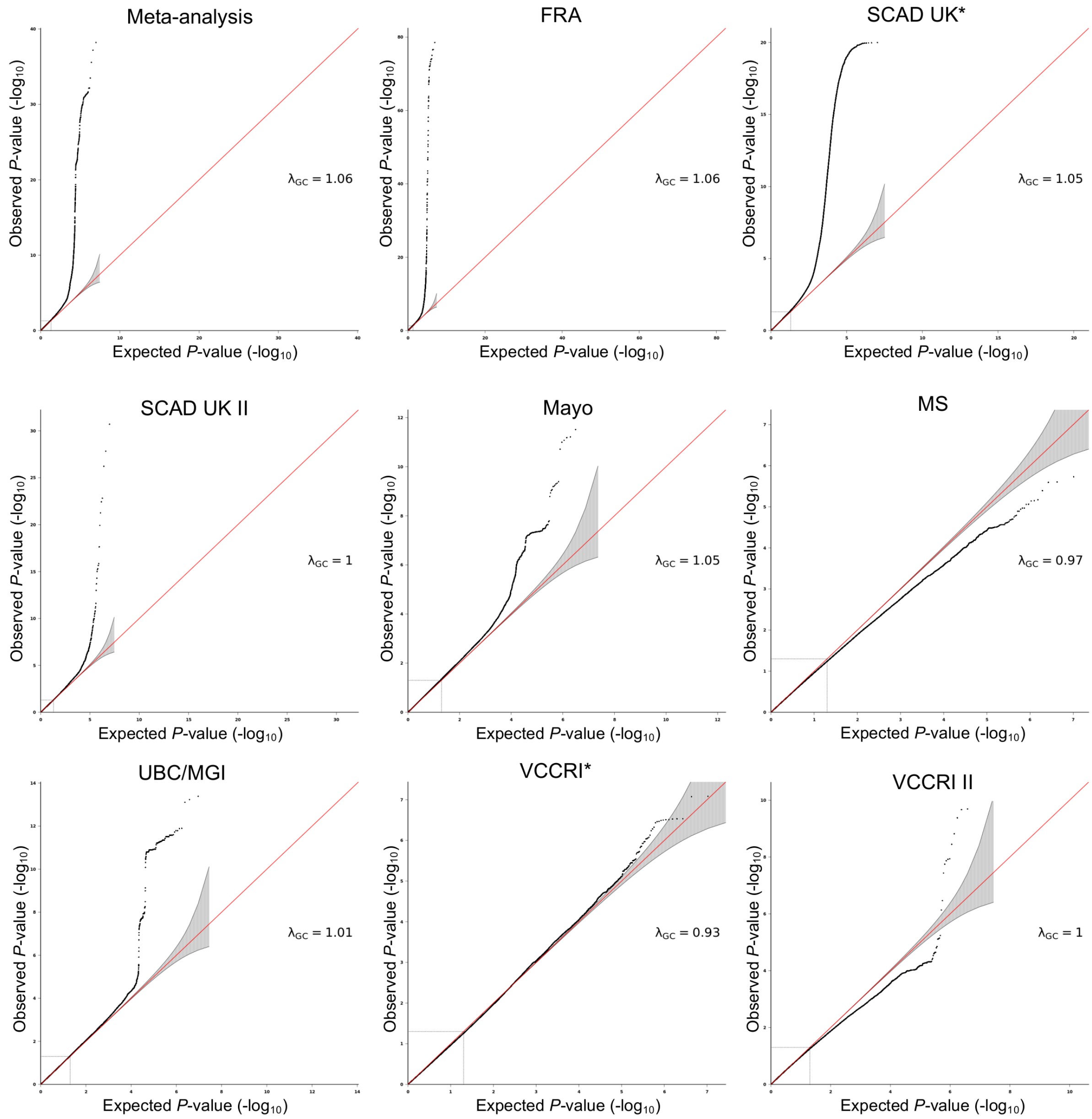

Supplementary Figure 2. Quantile-quantile plot representation of SNP-based association analysis in individual studies.

$-\log_{10}$ of observed association *P*-value value is represented on the y-axis, expected *P*-value on the x-axis. 95% concentration band (grey band) that is formed by calculating the 2.5th and 97.5th centiles of the distribution of the order statistic under random sampling and the null hypothesis. Genomic control value (λ_{GC}) is indicated for each study.

.

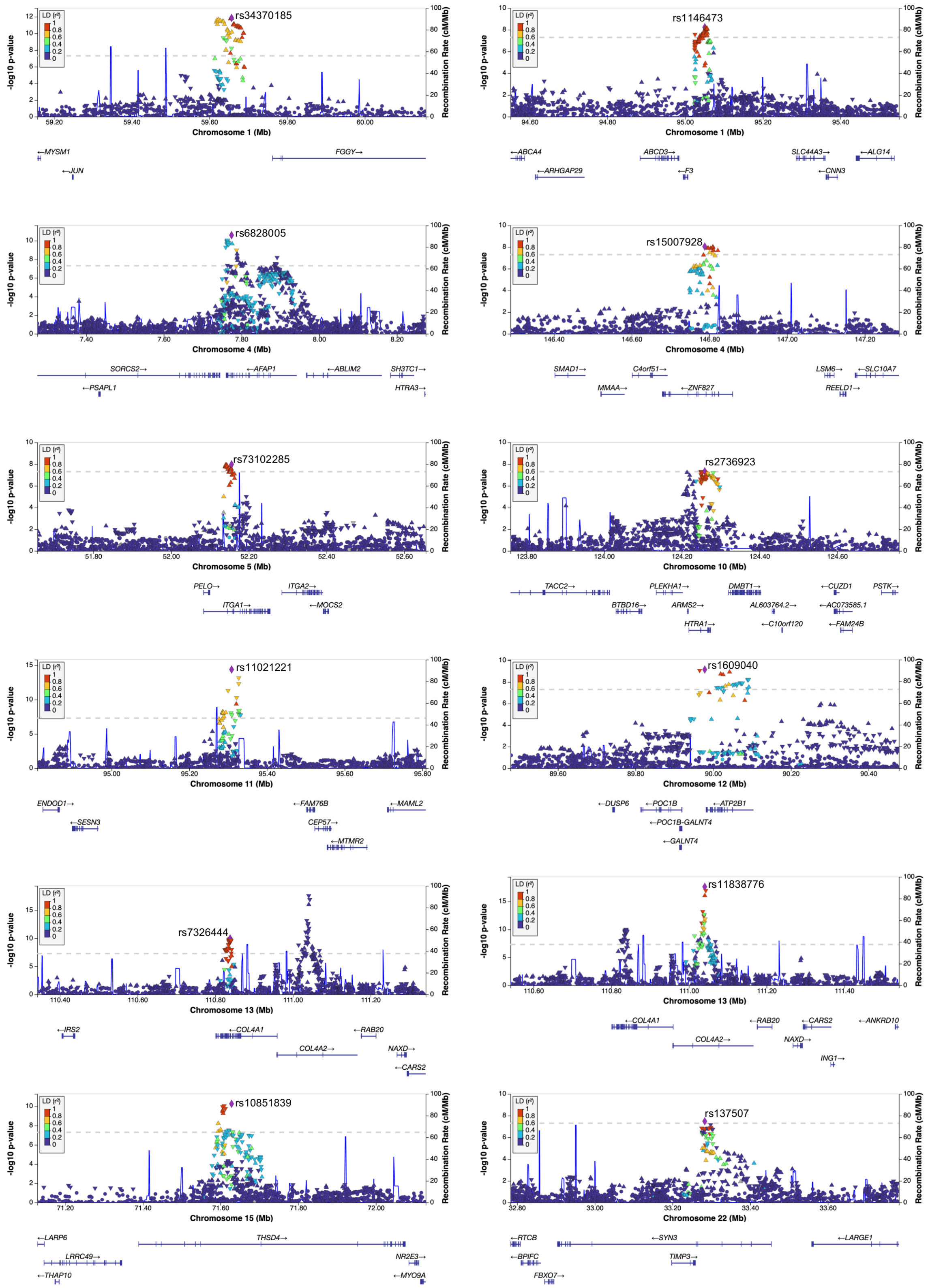

Supplementary Figure 3. Newly identified SCAD genetic risk loci. LocusZoom plots represent SCAD association at 12 loci (including 2 independent signals close to *COL4A1/COL4A2*). -$\log_{10}$ of association *P*-value (from a two-sided Wald test) is represented on the *y*-axis, genomic coordinates on the *x*-axis. RsIDs of top associated SNPs are indicated. Dot shape indicate the lead SNP (diamond shape) and effect size (β) of nominally significant variants (upper triangle: β>0, lower triangle, β<-0, round shape: p-value>0.05). Dot-color indicates linkage disequilibrium (r^2) with the lead SNP at each locus in the European subset of 1000G reference panel.

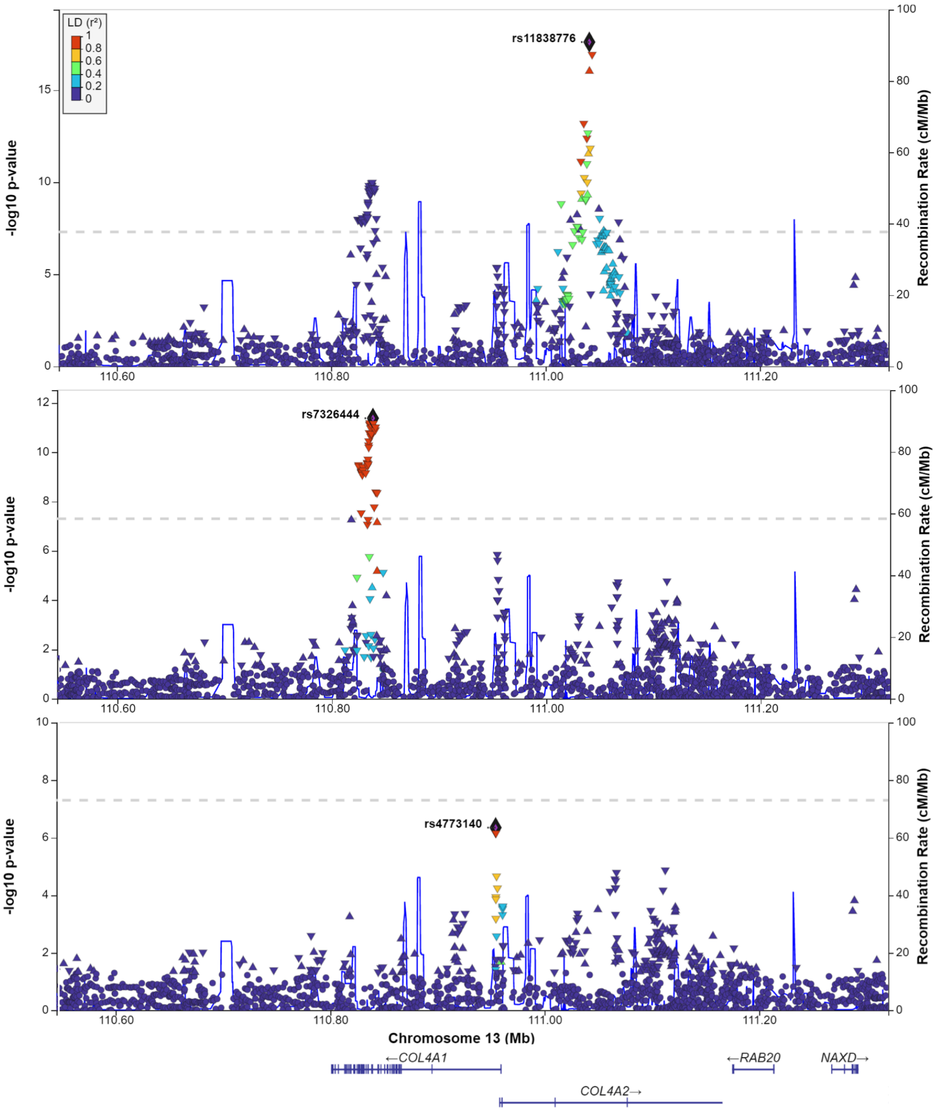

Supplementary Figure 4. Conditional analyses to illustrate 2 independent association signals on the *COL4A1/COL4A2* locus. LocusZoom plots represent SCAD associated SNPs on chromosome 13 near the *COL4A1/COL4A2* locus (A), after conditioning on to associated SNP rs11838776 (B) and after conditioning on rs11838776 and rs7326444, that showed independence from rs11838776 (C). $-\log_{10}$ of association *P*-value (from a two-sided Wald test) is represented on the *y*-axis, genomic coordinates on the *x*-axis. Dot shape indicates the lead SNP (diamond shape) and effect size (β) of nominally significant variants (upper triangle: β>0, lower triangle, β<-0, round shape: P-value>0.05). Dot-color indicates linkage disequilibrium (r^2) with the lead SNP at each locus in the European subset of the 1000G reference panel.

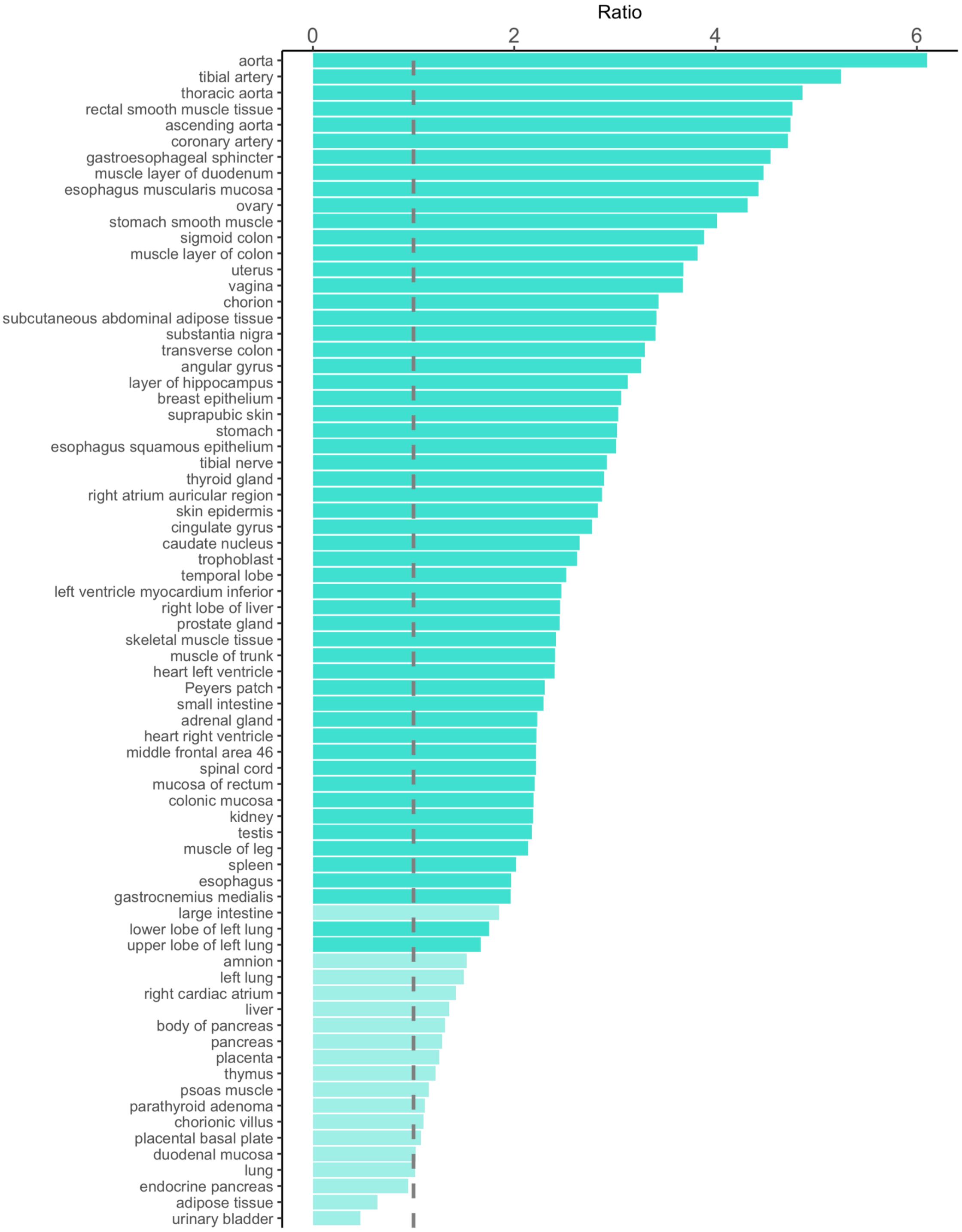

Supplementary Figure 5. Representation of SCAD SNPs enrichment in open chromatin regions from 73 tissues with available H3K27ac ChIP-Seq experiments in ENCODE database. For each tissue, H3K27ac narrowpeak files of all experiments were merged to generate one dataset. SCAD potential functional SNPS (95% credible set and LD proxies) were matched to random pools of neighbouring SNPs using GREGOR package[48]. Enrichment represents the ratio of the number of SCAD SNPs overlapping open chromatin regions over the average number of matched SNPs overlapping the same regions. *P*-value was evaluated using a binomial test with greater enrichment as alternative hypothesis (one-tailed), and is indicated by a brighter color for significant enrichment (Bonferroni adjusted *P*-value <0.05)

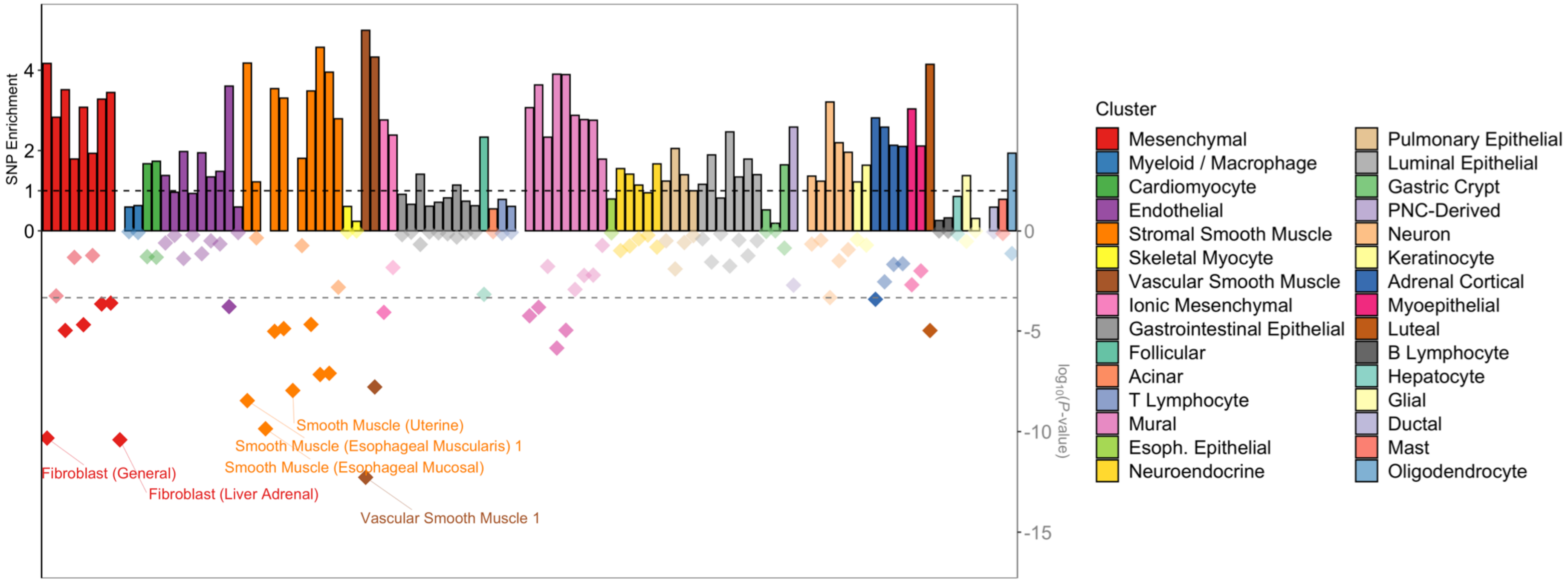

Supplementary Figure 6. Enrichment of SCAD SNPs in open chromatin regions from human adult cells. Representation of SCAD SNPs fold-enrichment (upper y-axis) and enrichment *P*-value (log scale, lower y-axis) among open chromatin regions of 105 sub clusters determined from single-nuclei ATAC-Seq in 30 adult tissues[19]. SCAD potential functional SNPS (95% credible set and LD proxies) were matched to random pools of neighbouring SNPs using GREGOR package[48]. Enrichment represents the ratio of the number of SCAD SNPs overlapping open chromatin regions over the average number of matched SNPs overlapping the same regions. *P*-value was evaluated using a binomial test with greater enrichment as alternative hypothesis (one-tailed). Bar colour represents the main cell clusters which are defined in the legend. The names of the 5% sub clusters with lower P-value for SCAD SNPs enrichment are indicated over the graph

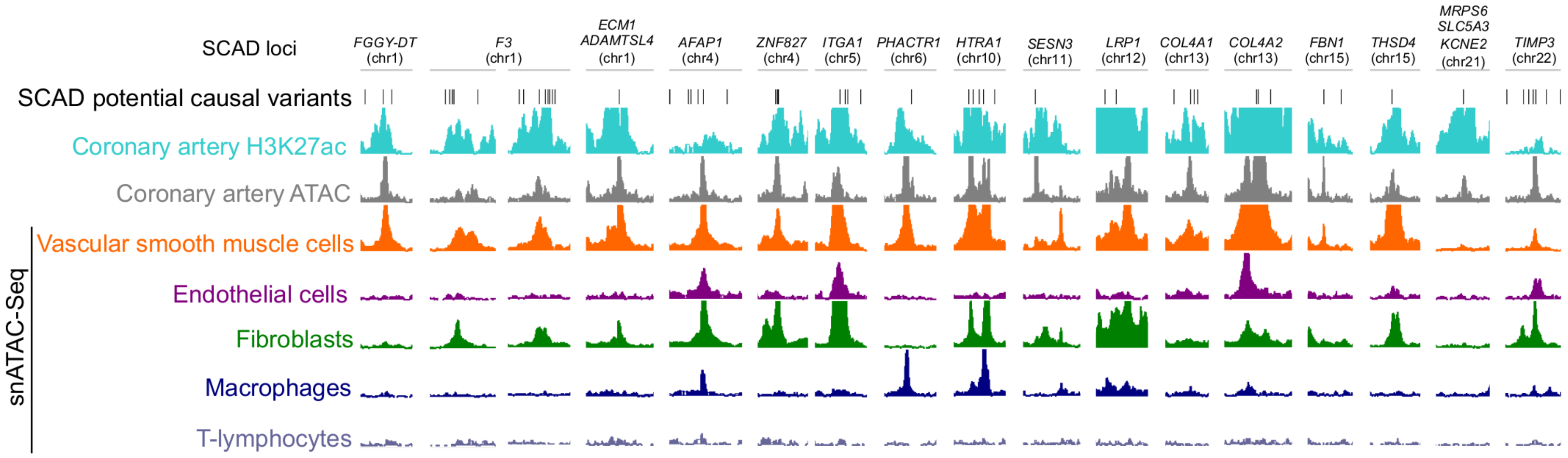

Supplementary Figure 7. SCAD associated variants overlapping with chromatin marks indicating potential regulatory regions. Genome browser visualization of H3K27ac ChIP/ATAC-Seq/snATAC-Seq read densities (in reads/million, r.p.m.) in the regions surrounding putative SCAD causal variants. snATAC-Seq density profiles correspond to sub clusters representing more than 1% of cells in artery tissue, and grouped by main cell type cluster[19]. A complete list of SCAD variants overlapping functional regions in artery tissue is available in a Supplementary file.

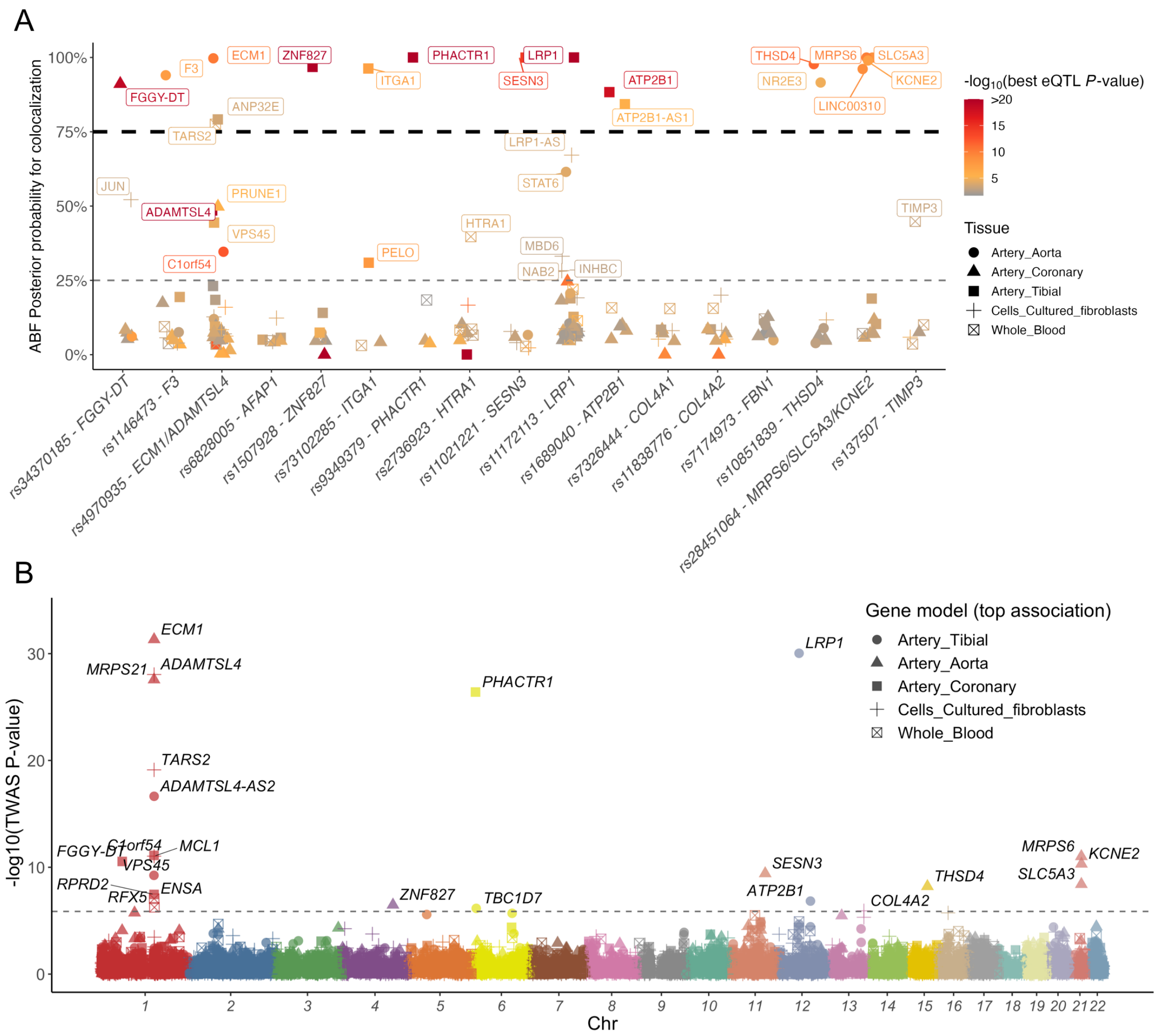

Supplementary Figure 8. Integration of SCAD genetic association with expression of potential target genes in relevant tissues.

A: Colocalization of eQTL and GWAS association at SCAD loci. Colocalization was assessed for all genes located within 500kb of SCAD top SNPs in five relevant tissues from GTEx (v8 release). *y*-axis represents the approximate Bayes factor posterior probability for eQTL and GWAs association to share one common variant at the locus. Dot color represents the *P*-value of top eQTL association involving variants at the locus, which were obtained for each variant-gene pair by testing the alternative hypothesis that the slope of a linear regression model between genotype and expression deviates from 0 and retrieved from GTEx database (v8 release)[29]. Only the tissue with highest probability for colocalization is represented for each gene, and indicated by the dot shape. Genes are grouped per SCAD lead SNP ranked by genomic position along *x*-axis. Names of genes with colocalization probability > 25% are indicated **B:** Manhattan plot representation of Transcriptome-wide association analysis (TWAS) in SCAD with gene expression models computed in 5 tissues based on GTEx (v8 release). −log10 of association *P*-value is represented on the *y*-axis, genomic coordinates on the x-axis. TWAS P-value was calculated using a two-tailed Z-test against a null distribution calculated by permutation for each gene/tissue.[56] Name of genes with Bonferroni corrected *P*-value < 0.05 are indicated.

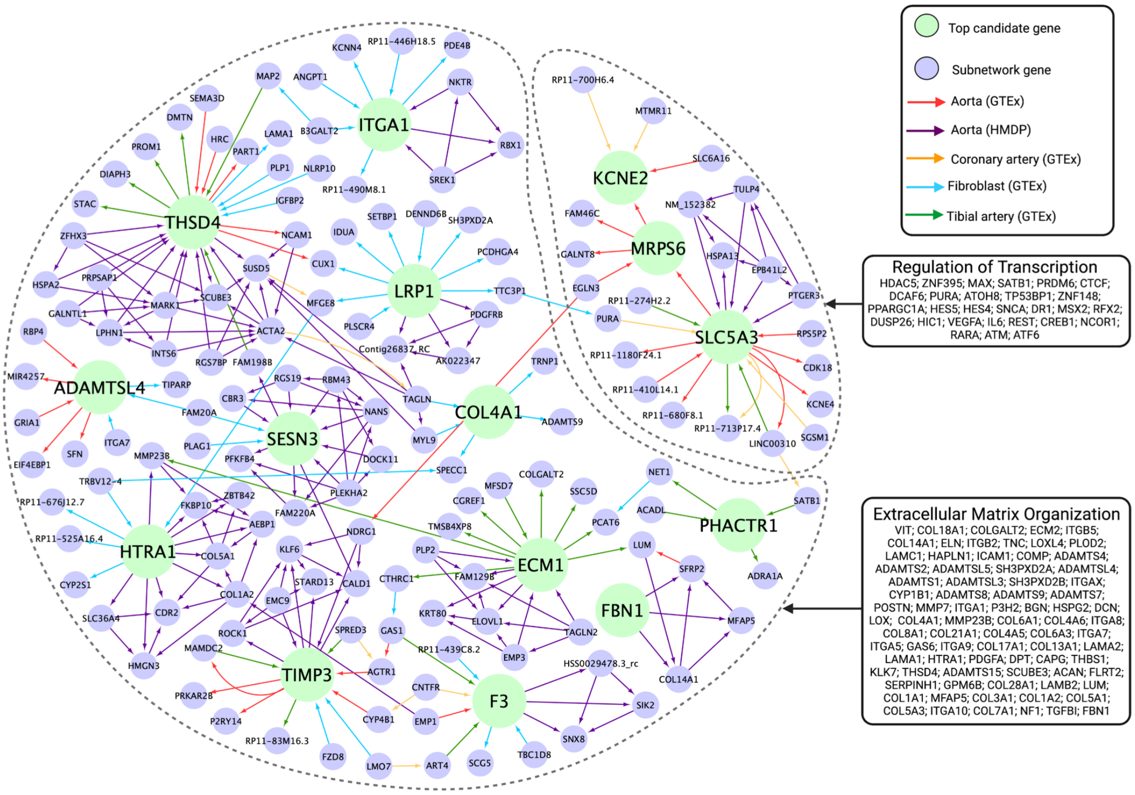

Supplementary Figure 9. Bayesian networks constructed using gene expression of top candidate genes in SCAD risk loci.

Tissue-specific Bayesian networks (BN) were constructed from the Genotype-Tissue Expression project Version 8 and Hybrid Mouse Diversity Panel gene expression data via RIMBANET. The BNs representing tissues and cell types relevant to SCAD onset were queried for the top GWAS hits. The top GWAS hits are emphasized by their larger size and color (green), whereas their surrounding subnetwork gene are shown in a smaller size and contrasting color (purple). The network edges inform on the source of the connection and the direction of regulation. Genes one edge away of the top GWAS hits are depicted within the network, whereas genes within two edges away from the GWAS hits were considered for pathway annotation. Suggested biological pathways in which denoted subnetworks function as well as the genes involved in the pathway are indicated.

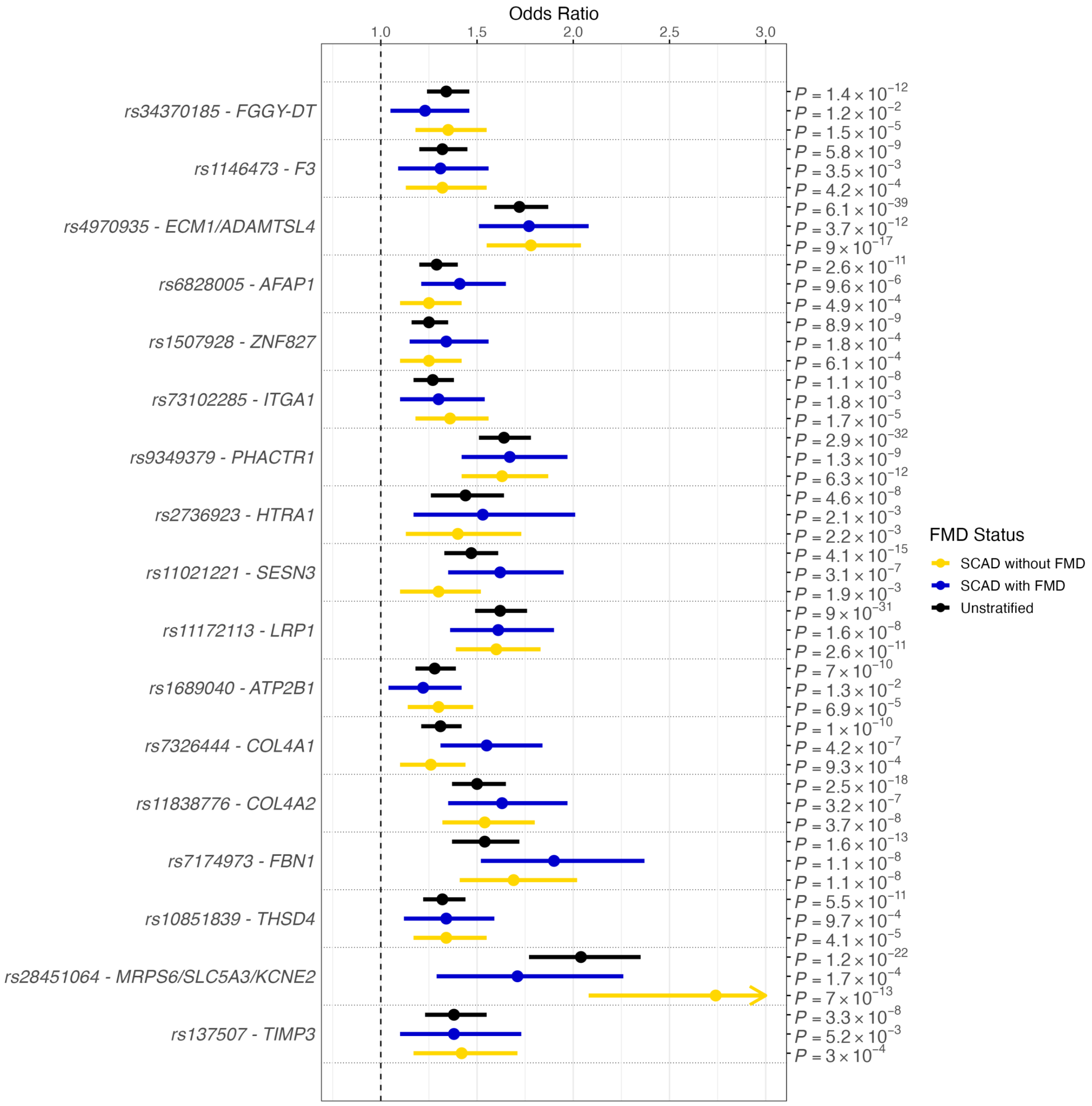

Supplementary Figure 10. Association of lead SNPs with SCAD according to FMD status. Forest plot representing the association of each SCAD top SNP in the general meta-analysis with 8 studies (N_{cases}=1,917 / $N_{controls}$=9,292) [black] as well as in the FMD stratified meta-analyses, FMD+ (N_{cases}=409 / $N_{controls}$=4,961) [blue] and FMD- (N_{cases}=614 / $N_{controls}$=5,776) [yellow] with 4 studies each. Odds Ratio of the association (centre for the error bars) is represented on the x-axis and range represents the 95 % confidence interval. Each association's P-value was obtained from two-sided Wald test and is indicated with the trait on the y-axis.

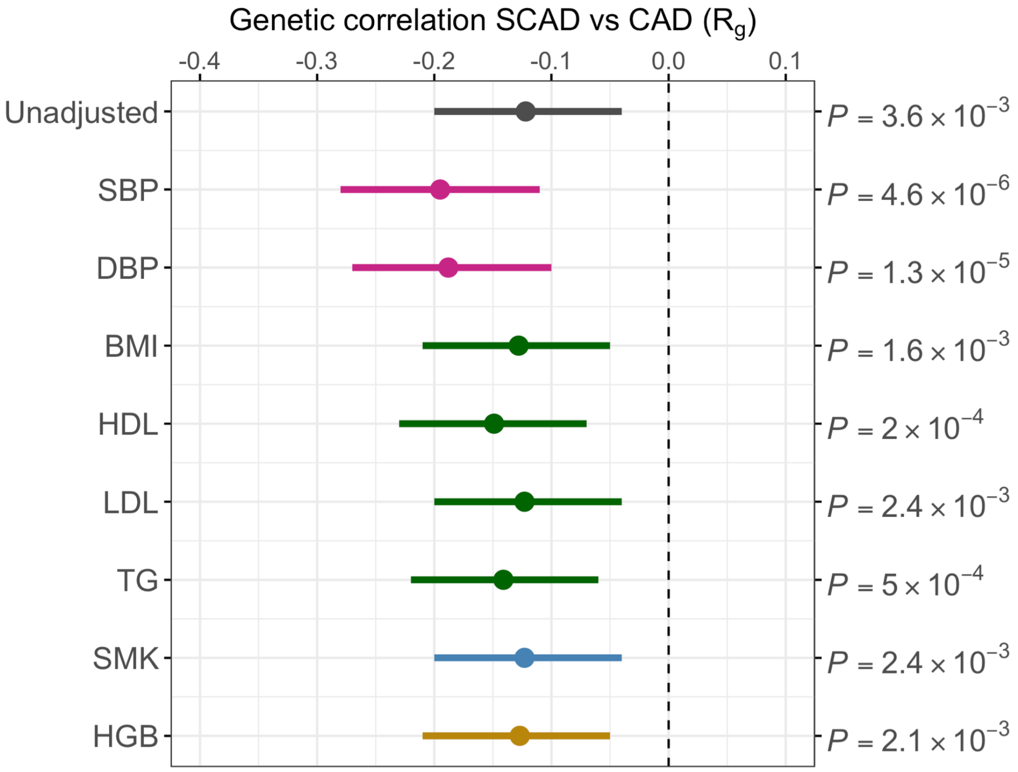

Supplementary Figure 11. **Forest plot representing the genetic correlation between SCAD (N_{cases}=1,917 / $N_{controls}$=9,292) and CAD (N_{cases}=181,522 / $N_{controls}$=984,168) unadjusted, and after mtCOJO conditioning on cardiometabolic traits**. Before re-estimation, SCAD genetic association statistics were conditioned on SBP: Systolic blood pressure (N=340,159), DBP: Diastolic blood pressure (N=340,162), BMI: Body mass index (N= 359,983), HDL: High-density lipoprotein (N=315,133), LDL: Low-density lipoprotein (N=343,621), TG: Triglycerides (N=343,992), SMK: Smoking (N_{cases}=164,638 / $N_{controls}$= 195,068) and HGB: Haemoglobin (N=408,112). Rho coefficient of Rg: genetic correlation (centre for the error bars) is represented on the *x*-axis and range represents the 95 % confidence interval. Undajusted *P*-values of genetic correlation were obtained from two-sided Wald test and are indicated with the trait on the *y*-axis.

Supplementary Tables

Supplementary Table 1: Clinical characteristics of the study populations

BMI: Body-Mass Index, HTN: Hypertension, n: count, Q1: 25% quantile, Q3: 75% quantile

Study	French Study		SCAD-UK Study I		SCAD-UK Study II		Mayo Clinic Study	
	Cases (DISCO)	Controls (3C-Study)	Cases	Controls	Cases	Controls	Cases	Controls
Type	Clinical based	Population based	Clinical based	Population based	Clinical based	Population based	Clinical based	Healthy volunteers
Inclusion criteria	age> 18,<br>1) retrospective with a diagnostic of SCAD made from 2010, or 2) prospective at the time of hospitalisation during which the diagnosis of SCAD was made.	Geographic sampling	SCAD confirmed on invasive angiography		SCAD confirmed on invasive angiography		SCAD confirmed by angiogram	No reported SCAD
Exclusion criteria	Age <18; atherosclerotic ischemic disease; iatrogenic hematoma	Age < 65y	Atherosclerotic dissection, iatrogenic dissection		Atherosclerotic dissection, iatrogenic dissection		Diagnosis of connective tissue disorder or aortopathy; iatrogenic	Diagnosis of atherosclerotic coronary artery disease, acute myocardial infarction, FMD, arterial aneurysm or dissection, cerebral infarction, Marfan syndrome, Ehlers-Danlos syndrome
Total (n)	313	1487	383	1925	139	815	506	1549
Women (n,%)	285 (91)	876 (58.9)	361 (94.2)	1815 (94.3)	115 (82.7)	665 (81.6)	484	1477
Age at study inclusion (Median, Q1,Q3)	51, 44, 59	74.36 ± 5.5<br>[65 - 94]		56,49,62		56, 48, 61	46.6 ± 9.2	64 ± 14.5
Age at SCAD (Median, Q1/Q3)	52.2, 44.55, 60	NR	47, 41, 52	NR	49.0, 43, 54	NR	46.6, 39, 53	NR
SCAD Type (1, 2, 3)	49, 237, 32	NR		NR		NR	Single vessel-397;<br>Multi-vessel-87	NR
FMD (Yes, No, NA)	140, 152, 21	NR	104,108,171		20,71,48		175, 140, 169	unknown
BMI (kg/m2)	**58**	NA	median 25 (23,29)<br>6 missing values		27 (23,31)<br>1 missing value)	NA	26.0 ± 5.9	unknown
HTN (n,%)	96 (30.7)	1171 (78.7)	94 (24.6; 1 missing value)		25 (18.0)	NA	157 (32.4)	unknown
T2D (n,%)	12 (3.2)		7 (1.8)		2 (1.4%)	NA	14 (2.9)	unknown
Migraine (n,%)	76 (28.4)		176 (48,9; 23 missing values)		67 (48.2; 2 missing values)	NA	175 (36.2)	unknown
Smoking (Non/Current/Ex)	Ever<br>220/93		266/14/103		94/9/36<br>(67, 6, 26)	NA	343/ 12 /129<br>(71 /2.5/26.7)	unknown

Study	CanSCAD/MGI Study		DEFINE-SCAD Study		VCCRI Study I		VCCRI Study II	
	Cases	Controls	Cases	Controls	Cases	Controls	Cases	Controls
Type	Clinical based	Population based	Clinical based	Clinical based	Clinical based	Population based	Clinical based	Clinical Based
Inclusion criteria	SCAD diagnosis was confirmed on coronary angiography by the UBC core laboratory research team, and categorized according to previously established Saw classification	Age, Sex, PC (PC1-PC3) matched controls	SCAD confirmed on invasive angio	Vascular disease excluded on history and physical exam. Also matched to SCAD cases by age, BMI, sex.	SCAD confirmed by angiogram	No reported SCAD	SCAD confirmed by angiogram	No Reported SCAD
Exclusion criteria	Angiogram unavailable or did not appear to be SCAD; from N=502, only Canadian samples consistent with 1000G non-Finish European ancestry (+/- 6 SD of PC1 and PC2) were retained for analysis.	Of 13,756 MGI samples eligible for the study after exclusion of vascular or connective tissue diagnoses, and matching for age, sex and ancestry (based upon genetic PC's) 2,125 matched MGI controls were retained for analysis.	Age < 18, diagnosis of connective tissue disorder or aortopathy; iatrogenic. Plus several other exclusions (HIV+, active malignancy)	Multiple - mostly relate to exclusion of vascular disease and other things like HIV+, active malignancy)	Angiogram unavailable or did not appear to be SCAD	No reported history of cancer, cardiovascular disease or neurodegenerative diseases before 70 years old	Angiogram unavailable or did not appear to be SCAD	Related to other sample
Total (n)	357	2125	42	153	88	1127	85	111
Women (n,%)	315 (88.2%)	1873 (88.1%)	41 (97.6%)	153 (100%)	80, 90.9%	672, 59.6%	83, 97.6%	46, 41.4%
Age at study inclusion (Median, Q1,Q3)	53, 46, 60	53, 46, 61	49, 41.5, 53.75	50 (43-58)	50, 44, 59	all >70 years old	52, 48, 60	61, 52, 67
Age at SCAD (Median, Q1/Q3)		NR	45.5, 36, 50.25<br>6 missing values	NA	44, 39, 52	NA	49, 43, 56	NA
SCAD Type (1, 2, 3)	117,193,36	NR	4; 32; 1; NA: 5	NR	32, 50, 4,<br>(2 NR)	NA	29, 48, 6	NA
FMD (Yes, No, NA)	149,123,85	NR	31, 10, 1	NR	14, 32, 42	NR	10, 22, 53	NR
BMI (kg/m2)	median 25.5 (22,30;9 missing values)	NA	24.55 (22, 29.15)	23.85 (20.98, 26.6)	26.32	27.5	27.04	NR
HTN (n,%)	108 (30%); 33 missing values	NA	18 (42.86%)	4 (2.61%)	17, 19%	NR	19, 22.3%	NR
T2D (n,%)	9 (2.5%); 33 missing values	NA	0 (0%)	1 (0.65%)	3, 3.4%	NR	6, 7%	NR
Migraine (n,%)	100 (28%); 33 missing values	NA	9 (21.4%)	NA	41, 46.6%	NR	48, 56.5%	NR
Smoking (Non/Current/Ex)	211/25/88	NA	34/1/7	NA	54/ 3/ 28	NR	51/ 3/ 31	NR

Supplementary Table 2 : Lead associated variants at genome-wide significant SCAD loci in each study and meta-analysis.

CHR: chromosome, POS: position, EA: effect alleles, OA: other alleles. EAF: effect allele frequencies.

OR: Odds ratio, P: Unadjusted P-values of association obtained from two-sided Wald test, Het-P: P-value from the Cochran Q statistic heterogeneity test.

Direction signs are provided for the individual association results in DISCO-3C, SCAD-UKI, Mayo Clinic, UBC/MGI, VCCRI, SCAD-UK II, VCCRI II and DEFINE-SCAD studies, respectively. Grey lines correspond to previously reported loci in SCAD

						**DISCO-3C**		**SCAD-UK I**		**Mayo Clinic**		**CanSCAD/MGI**		**VCCRI I**		**SCAD-UK II**		**VCCRI II**		**DEFINE-SCAD**		**Meta-analysis**			
**Locus**	**CHR:POS**	**rsID**	**Annotated Genes**	**EA**	**EAF**	**ORs**	**P**	**ORs**	**P**	**ORs**	**P**	**ORs**		**ORs**	**P**	**ORs**	**P**	**ORs**	**P**	**ORs**	**P**	**Direction**	**ORs**	**P**	**HET-P**
1	1:59656909	rs34370185	*FGGY-DT*	T	0.29	1.08	4.87E-01	1.46	4.84E-05	1.47	1.58E-06	1.17	7.37E-02	1.85	2.24E-04	1.31	6.15E-02	1.25	4.55E-01	2.01	1.79E-02	++++++++	1.34	1.42E-12	4.36E-02
2	1:95050472	rs1146473	*F3*	C	0.19	1.05	6.71E-01	1.30	1.65E-02	1.42	6.56E-05	1.20	7.43E-02	1.20	3.48E-01	1.91	1.30E-04	1.95	8.41E-02	1.84	6.07E-02	++++++++	1.32	5.82E-09	9.80E-02
3	1:150504062	rs4970935	*ECM1/ADAMTSL4*	C	0.28	1.73	3.41E-08	1.70	3.38E-08	1.76	1.86E-12	1.85	1.30E-12	1.48	1.99E-02	1.37	3.98E-02	2.46	4.18E-03	1.57	9.71E-02	++++++++	1.72	6.14E-39	6.36E-01
4	4:7774352	rs6828005	*AFAP1*	G	0.45	1.21	4.62E-02	1.28	6.63E-03	1.34	8.79E-05	1.27	3.43E-03	1.14	4.23E-01	1.52	1.25E-03	1.15	5.71E-01	1.50	8.91E-02	++++++++	1.29	2.60E-11	8.16E-01
5	4:146788035	rs1507928	*ZNF827*	C	0.48	1.21	4.46E-02	1.15	1.28E-01	1.38	1.79E-05	1.15	9.33E-02	1.08	6.25E-01	1.53	1.93E-03	1.57	1.03E-01	1.39	1.93E-01	++++++++	1.25	8.94E-09	3.85E-01
6	5:52155642	rs73102285	*ITGA1*	G	0.27	1.34	4.62E-03	1.47	8.34E-05	1.23	1.24E-02	1.27	7.26E-03	1.44	2.96E-02	1.06	7.04E-01	0.81	4.65E-01	0.90	7.34E-01	++++++--	1.27	1.05E-08	3.12E-01
7	6:12903957	rs9349379	*PHACTR1*	A	0.62	1.68	4.08E-07	1.38	6.70E-04	1.76	6.52E-12	1.74	7.36E-10	1.80	8.67E-04	1.50	2.85E-03	2.71	5.40E-04	1.09	7.87E-01	++++++++	1.64	2.88E-32	1.93E-01
8	10:124259062	rs2736923	*HTRA1*	A	0.89	1.39	3.36E-02	1.22	2.14E-01	1.60	3.36E-04	1.33	3.92E-02	1.37	2.59E-01	2.02	1.14E-03	-	-	1.28	5.08E-01	++++++?+	1.44	4.58E-08	6.00E-01
9	11:95308854	rs11021221	*SESN3*	A	0.17	1.65	4.08E-05	1.14	2.40E-01	1.41	2.64E-04	1.55	8.41E-06	1.57	2.43E-02	2.04	6.23E-05	1.27	4.90E-01	1.30	4.73E-01	++++++++	1.47	4.11E-15	1.92E-01
10	12:57527283	rs11172113	*LRP1*	T	0.62	1.67	6.20E-07	1.83	5.63E-10	1.64	8.56E-10	1.46	1.09E-05	1.78	1.53E-03	1.39	1.89E-02	1.56	1.05E-01	1.89	3.63E-02	++++++++	1.62	9.03E-31	7.02E-01
11	12:89978233	rs1689040	*ATP2B1*	C	0.59	1.29	8.13E-03	1.32	2.11E-03	1.24	5.82E-03	1.35	5.28E-04	1.59	7.38E-03	1.12	4.07E-01	1.14	6.64E-01	0.88	6.68E-01	+++++++-	1.28	7.04E-10	6.60E-01
12	13:110838236	rs7326444	*COL4A1*	G	0.64	1.34	3.80E-03	1.27	1.37E-02	1.31	8.93E-04	1.43	5.42E-05	1.05	7.76E-01	1.12	4.17E-01	1.38	2.53E-01	2.03	1.82E-02	++++++++	1.31	1.02E-10	5.17E-01
12	13:111040681	rs11838776	*COL4A2*	G	0.73	1.58	3.62E-05	1.59	3.12E-05	1.52	4.43E-06	1.55	1.24E-05	1.06	7.68E-01	1.52	6.10E-03	1.83	4.22E-02	0.96	8.74E-01	+++++++-	1.50	2.46E-18	4.20E-01
13	15:48763754	rs7174973	*FBN1*	G	0.11	2.16	2.70E-10	1.38	2.16E-02	1.66	3.48E-06	1.09	5.29E-01	1.36	1.85E-01	1.56	5.52E-02	1.35	5.33E-01	1.40	4.28E-01	++++++++	1.54	1.60E-13	2.96E-02
14	15:71628370	rs10851839	*THSD4*	A	0.68	1.08	4.13E-01	1.46	2.04E-04	1.40	7.59E-05	1.44	6.86E-05	1.13	4.98E-01	1.38	2.34E-02	0.91	7.06E-01	1.55	1.50E-01	++++++-+	1.32	5.51E-11	2.37E-01
15	21:35593827	rs28451064	*MRPS6/SLC5A3/KCNE2*	G	0.88	2.58	1.08E-06	2.09	2.10E-05	2.17	8.42E-08	1.90	1.42E-05	2.79	2.52E-03	1.65	1.42E-02	1.01	9.78E-01	1.87	2.17E-01	++++++++	2.04	1.16E-22	4.97E-01
16	22:33282971	rs137507	*TIMP3*	T	0.11	1.79	4.78E-06	1.02	9.20E-01	1.30	2.04E-02	1.44	2.30E-03	1.51	8.47E-02	0.91	6.61E-01	3.15	1.36E-02	2.03	4.65E-02	+++++-++	1.38	3.30E-08	1.89E-02

Supplementary Table 3: SNP heritability estimates
Values are given on the observed scale (h2obs) and liability scale (h2liab).
Prevalence used for conversion to the liability scale is shown.
Effective number samples were used for the conversion, Neff = 4/(1/Ncases+1/NControls).
For SumHer, two analyses were done: one assuming LDAK-THIN model, using a pre-calculated tagging file obtained by using LD reference data from UKBB, and one to mimic LDSC, with the same settings and reference panel (HapMap3 and 404 nonFin european from 1000G project).
Neff, effective sample size.

Trait	Method	*h* 2obs (s.e.)	*intercept (s.e.)*	Prevalence	*h* 2liab (s.e.)	Cases	Controls	Neff
SCAD	LDSC	0.73 (0.11)	1.016 (0.0072)	0.01	0.71 (0.11)	1917	9292	6357
SCAD	SumHer (LDSC)	0.77 (0.10)	1.020 (0.0079)	0.01	0.75 (0.10)	1917	9292	6357
SCAD	SumHer (LDAK-THIN)	0.71 (0.12)	1.027 (0.0088)	0.01	0.70 (0.12)	1917	9292	6357

Supplementary Table 4: SNP heritability of the top loci region (1 Mb)
The table shows local heritability estimation, p-values obtained from partitionned heritability using SumHer
LOCUS: region of 1 Mb named on lead SNP (GWAS hit), CHROM: chromosome,START: position of lead SNP - 500k bp, STOP: position of lead SNP + 500k bp,EA: effect allele, h2obs: observed heritability estimation, SD: standard deviation of h2obs

Locus	Candidate Genes	Lead SNP	CHROM	START	STOP	h2obs	SD
1	*FGGY-DT*	rs34370185	1	59156909	60156909	0.013	0.005
2	*F3*	rs1146473	1	94550472	95550472	0.009	0.004
3	*ECM1/ADAMTSL4*	rs4970935	1	150004062	151004062	0.028	0.012
4	*AFAP1*	rs6828005	4	7274352	8274352	0.012	0.004
5	*ZNF827*	rs1507928	4	146288035	147288035	0.005	0.003
6	*ITGA1*	rs73102285	5	51655642	52655642	0.011	0.005
7	*PHACTR1*	rs9349379	6	12403957	13403957	0.012	0.004
8	*HTRA1*	rs2736923	10	123759062	124759062	0.012	0.004
9	*SESN3*	rs11021221	11	94808854	95808854	0.012	0.005
10	*LRP1*	rs11172113	12	57027283	58027283	0.019	0.007
11	*ATP2B1*	rs1689040	12	89478233	90478233	0.008	0.004
12	*COL4A1*	rs7326444	13	110338236	111540681	0.022	0.006
	COL4A2	rs11838776	13				
13	*FBN1*	rs7174973	15	48263754	49263754	0.003	0.002
14	*THSD4*	rs10851839	15	71128370	72128370	0.007	0.003
15	*MRPS6/SLC5A3/KCNE2*	rs28451064	21	35093827	36093827	0.013	0.005
16	*TIMP3*	rs137507	22	32782971	33782971	0.007	0.003
	**all 16 loci (all SNPs)**	-	-	-	-	0.188	0.018

Supplementary Table 5: Annotation of candidate functional SNPs

We display SNPs previously identified as potential functional SNPs (LD r2 > 0.7 with the lead SNPs or 95% credible set) and overlapping with either H3K27ac enriched regions or open chromatin regions in coronary artery or any of 5 single-cell clusters represented in artery tissue (SMCs, endothelial cells, fibroblasts, macrophages and T-lymphocytes). Lead SNP from each locus is indicated in bold and comes first before other SNPs in the locus. Other SNPs are ranked by genomic coordinates (hg38 genome). Manually curated best candidates SNPs are indicated by a "*". Overlap with H3K27ac signal or open chromatin region is indicated by a "+".

Chr: chromosome, r2: r2 of linkage disequilibrium with lead SNP of the locus (European population in 1000 Genome reference panel), PP: posterior probability, EA: Effect allele, EAF: effect allele frequency, P: Unadjusted P-values of association obtained from two-sided Wald test,OR: Odds ratio, CI: confidence interval.

Locus	Candidate Gene(s)	Candidate SNP	SNP	Chr	Position (hg38)	Alleles	r2	Correlated Alleles	PP	EA	EAF	Direction	P	OR (95% CI)	Coronary artery H3K27ac	Coronary artery ATAC-Seq	snATAC-Seq vascular SMCs	snATAC-Seq ECs	snATAC-Seq Fibroblasts	snATAC-Seq Macrophages	snATAC-Seq T-lymphocytes
1	*FGGY-DT*		**rs34370185**	**chr1**	**59656909**	**(G/T)**	**-**	**-**	**10.9%**	**G**	**0.71**	**--------**	**1.42E-12**	**0.74 (0.69-0.81)**							
			rs3737157	chr1	59617813	(A/G)	0.71	G=A,T=G	3.8%	G	0.30	++++++++	4.40E-12	1.33 (1.23-1.45)	+						
			rs12730605	chr1	59627712	(T/C)	0.71	G=T,T=C	5.2%	C	0.30	++++++++	2.94E-12	1.33 (1.23-1.45)	+						
			rs11207414	chr1	59635514	(G/T)	0.71	G=G,T=T	3.9%	G	0.70	--------	4.08E-12	0.75 (0.69-0.81)		+					
			rs12745935	chr1	59644082	(A/G)	0.71	G=A,T=G	na	na	na	na	na	na	+	+			+		
		*	rs12746365	chr1	59644084	(G/A)	0.71	G=G,T=A	na	na	na	na	na	na	+	+			+		
		*	rs17535443	chr1	59646056	(G/A)	0.77	G=G,T=A	0.5%	G	0.72	--------	3.40E-11	0.76 (0.7-0.82)	+	+					
		*	rs11207420	chr1	59646524	(G/A)	0.77	G=G,T=A	0.4%	G	0.72	--------	5.02E-11	0.76 (0.7-0.82)	+	+					
		*	rs12733512	chr1	59646978	(C/T)	0.77	G=C,T=T	0.1%	C	0.72	--------	1.53E-10	0.76 (0.7-0.83)	+	+					
			rs12130314	chr1	59651063	(G/T)	0.76	G=G,T=T	0.1%	G	0.72	--------	2.81E-10	0.77 (0.71-0.83)	+						
			rs12753566	chr1	59654019	(T/C)	1.00	G=T,T=C	0.0%	C	0.29	++?+++++	2.40E-08	1.31 (1.19-1.44)	+						
			rs12752853	chr1	59668705	(C/T)	0.96	G=C,T=T	0.0%	C	0.71	--?-----	4.52E-07	0.78 (0.71-0.86)	+						
			rs12739904	chr1	59669488	(T/C)	0.96	G=T,T=C	1.2%	C	0.29	++++++++	1.46E-11	1.32 (1.22-1.44)	+	+					
		*	rs12758643	chr1	59669918	(C/T)	0.96	G=C,T=T	0.0%	C	0.71	--?-----	4.05E-07	0.78 (0.71-0.86)	+	+	+	+	+	+	
		*	rs7543389	chr1	59680536	(A/G)	0.96	G=A,T=G	0.8%	G	0.29	++++++++	2.16E-11	1.32 (1.22-1.43)	+	+	+				
			rs10493256	chr1	59688948	(C/T)	0.82	G=C,T=T	0.0%	C	0.70	--?-----	1.07E-06	0.79 (0.72-0.87)	+	+			+		
2	*F3*		**rs1146473**	**chr1**	**95050472**	**(T/C)**	**-**	**-**	**6.5%**	**C**	**0.19**	**++++++++**	**5.82E-09**	**1.32 (1.2-1.45)**							
			rs1146481	chr1	95045247	(G/C)	0.96	T=G,C=C	1.4%	G	0.82	--------	3.09E-08	0.77 (0.7-0.84)	+						
			rs1146480	chr1	95045362	(G/A)	0.96	T=G,C=A	1.3%	G	0.82	--------	3.20E-08	0.77 (0.7-0.84)	+						
		*	rs12354005	chr1	95045461	(A/G)	0.95	T=A,C=G	0.7%	G	0.18	++++++++	6.04E-08	1.3 (1.18-1.43)	+	+	+		+		
			rs34379452	chr1	95046652	(-/TT)	0.96	T=-,C=TT	na	na	na	na	na	na		+					
			rs1772904	chr1	95055132	(C/T)	0.99	T=C,C=T	2.6%	C	0.81	--------	1.63E-08	0.77 (0.7-0.84)	+						
			rs968662	chr1	95055317	(T/A)	0.99	T=T,C=A	2.4%	T	0.81	--------	1.78E-08	0.76 (0.7-0.84)	+						
			rs968661	chr1	95055331	(A/G)	0.99	T=A,C=G	2.2%	G	0.19	++++++++	1.84E-08	1.31 (1.19-1.43)	+						
		*	rs1778208	chr1	95056122	(A/G)	1.00	T=A,C=G	5.4%	G	0.19	++++++++	7.19E-09	1.32 (1.2-1.44)	+	+	+		+		
		*	rs927636	chr1	95056425	(A/G)	1.00	T=A,C=G	4.4%	G	0.19	++++++++	8.67E-09	1.31 (1.2-1.44)	+	+			+		
		*	rs927635	chr1	95056533	(A/G)	0.99	T=A,C=G	1.2%	G	0.19	++++++++	3.44E-08	1.3 (1.18-1.43)	+	+					
		*	rs927634	chr1	95056635	(A/T)	0.99	T=A,C=T	4.5%	T	0.19	++++++++	8.35E-09	1.31 (1.2-1.44)	+	+					
3	*ECM1/ADAMTSL4*		**rs4970935**	**chr1**	**150504062**	**(C/T)**	**-**	**-**	**90.8%**	**C**	**0.28**	**++++++++**	**6.14E-39**	**1.72 (1.59-1.87)**							
			rs4970996	chr1	150506589	(G/C)	0.87	C=G,T=C	8.9%	G	0.27	++++++++	6.42E-38	1.72 (1.58-1.87)	+						
		*	rs6693567	chr1	150510660	(C/T)	0.90	C=C,T=T	0.0%	C	0.29	++?+++++	9.79E-29	1.7 (1.55-1.87)	+	+	+		+		
			rs7364686	chr1	150513711	(A/G)	0.73	C=A,T=G	na	na	na	na	na	na		+					
4	*AFAP1*		**rs6828005**	**chr4**	**7774352**	**(G/A)**	**-**	**-**	**31.6%**	**G**	**0.45**	**++++++++**	**2.60E-11**	**1.29 (1.2-1.4)**							
			rs2285764	chr4	7763161	(T/C)	0.31	G=T,A=C	4.5%	C	0.77	--------	1.91E-10	0.75 (0.69-0.82)		+					
			rs2285762	chr4	7763405	(T/C)	0.30	G=T,A=C	9.0%	C	0.77	--------	9.31E-11	0.75 (0.69-0.82)		+	+		+		
		*	rs2269851	chr4	7763605	(T/C)	0.30	G=T,A=C	9.6%	C	0.77	--------	8.82E-11	0.75 (0.69-0.82)		+	+		+	+	
			**rs1507928**	**chr4**	**146788035**	**(T/C)**	**-**	**-**	**13.1%**	**C**	**0.48**	**++++++++**	**8.94E-09**	**1.25 (1.16-1.35)**							
		*	rs1979974	chr4	146800815	(A/G)	0.97	T=A,C=G	0.0%	G	0.48	++?+++++	2.44E-05	1.21 (1.11-1.32)	+	+	+		+		
		*	rs17020769	chr4	146800922	(C/T)	0.97	T=C,C=T	0.0%	C	0.52	--?-----	6.04E-06	0.81 (0.75-0.89)	+	+	+		+		
		*	rs13128814	chr4	146801002	(G/A)	0.71	T=G,C=A	4.1%	G	0.49	--------	3.15E-08	0.81 (0.75-0.87)	+	+	+		+		

5	***ZNF827***		rs28590383	chr4	146803248	(T/C)	0.98	T=T,C=C	9.0%	C	0.48	++++++++	1.34E-08	1.25 (1.16-1.35)	+						
		*	rs7679068	chr4	146808682	(C/T)	0.75	T=C,C=T	0.0%	C	0.49	--?-----	5.59E-05	0.83 (0.76-0.91)	+	+	+	+	+		
		*	rs7662069	chr4	146809016	(T/G)	0.89	T=T,C=G	12.3%	G	0.49	++++++++	9.53E-09	1.25 (1.16-1.35)	+	+			+		
		*	rs7662070	chr4	146809017	(T/C)	0.90	T=T,C=C	10.7%	C	0.49	++++++++	1.11E-08	1.25 (1.16-1.35)	+	+			+		
			rs10000888	chr4	146809168	(A/G)	0.75	T=A,C=G	3.1%	G	0.51	++++++++	4.27E-08	1.24 (1.15-1.34)	+	+			+		
6	***ITGA1***	*	**rs73102285**	**chr5**	**52155642**	**(A/G)**	**-**	**-**	**7.3%**	**G**	**0.27**	**++++++--**	**1.05E-08**	**1.27 (1.17-1.38)**		+	+				
			rs6867399	chr5	52135543	(C/A)	0.70	A=C,G=A	0.0%	C	0.71	--?---+-	9.82E-06	0.81 (0.73-0.89)	+						
			rs73756268	chr5	52141109	(T/G)	0.97	A=T,G=G	5.1%	G	0.27	++++++-+	1.61E-08	1.27 (1.17-1.38)	+						
			rs73756269	chr5	52141120	(C/T)	0.97	A=C,G=T	4.9%	C	0.73	------+-	1.68E-08	0.79 (0.73-0.86)	+						
			rs12110170	chr5	52142544	(T/A)	0.97	A=T,G=A	6.6%	T	0.73	------+-	1.18E-08	0.79 (0.72-0.85)	+						+
			rs60422098	chr5	52143928	(A/G)	0.97	A=A,G=G	4.0%	G	0.27	++++++-+	2.12E-08	1.27 (1.17-1.37)	+						
			rs59192299	chr5	52144090	(T/G)	0.98	A=T,G=G	5.3%	G	0.27	++++++-+	1.53E-08	1.27 (1.17-1.38)	+						
			rs35302472	chr5	52145479	(-/A)	0.86	A=-,G=A	na	na	na	na	na	na				+			
			rs10513000	chr5	52149451	(A/G)	0.99	A=A,G=G	2.0%	G	0.27	++++++--	4.56E-08	1.26 (1.16-1.37)	+						
			rs10513001	chr5	52149529	(T/G)	0.99	A=T,G=G	0.1%	G	0.27	++?+++--	9.06E-07	1.27 (1.15-1.4)	+						
			rs10046016	chr5	52150178	(A/T)	0.98	A=A,G=T	2.1%	T	0.27	++++++--	4.24E-08	1.26 (1.16-1.37)	+						
			rs7704305	chr5	52150852	(A/G)	0.97	A=A,G=G	2.3%	G	0.27	++++++--	3.92E-08	1.26 (1.16-1.37)	+			+			
			rs73754055	chr5	52154837	(G/C)	0.99	A=G,G=C	3.2%	G	0.73	------++	2.59E-08	0.79 (0.73-0.86)	+						
			rs6886404	chr5	52154995	(A/C)	0.99	A=A,G=C	2.7%	C	0.27	++++++--	3.05E-08	1.26 (1.16-1.37)	+						
			rs73754057	chr5	52156781	(T/A)	0.95	A=T,G=A	0.9%	T	0.73	------++	9.93E-08	0.8 (0.73-0.87)	+						
			rs1478445	chr5	52163105	(G/A)	0.92	A=G,G=A	1.1%	G	0.73	------++	8.12E-08	0.8 (0.73-0.86)	+						
			rs6884370	chr5	52163352	(G/C)	0.91	A=G,G=C	0.5%	G	0.73	------++	2.05E-07	0.8 (0.74-0.87)	+						
7	***PHACTR1***	*	**rs9349379**	**chr6**	**12903957**	**(A/G)**	**-**	**-**	**100.0%**	**G**	**0.38**	**--------**	**2.88E-32**	**0.61 (0.56-0.66)**	+	+					
8	***HTRA1***	*	**rs2736923**	**chr10**	**124259062**	**(G/A)**	**-**	**-**	**4.9%**	**G**	**0.11**	**------?-**	**4.58E-08**	**0.69 (0.61-0.79)**	+	+	+		+	+	
			rs11200638	chr10	124220544	(G/A)	0.02	NA	1.2%	G	0.78	----+-+-	2.54E-07	0.79 (0.72-0.86)	+	+	+			+	
			rs2239588	chr10	124250093	(G/C)	0.82	G=C,A=G	0.5%	G	0.91	++++?+++	5.31E-07	1.47 (1.26-1.7)	+						
			rs2268349	chr10	124250384	(G/T)	0.97	G=T,A=G	3.4%	G	0.89	++++++++	6.54E-08	1.44 (1.26-1.64)	+						
		*	rs2268350	chr10	124251060	(C/T)	0.97	G=T,A=C	4.5%	C	0.89	++++++++	4.89E-08	1.44 (1.26-1.64)	+	+					+
			rs2268351	chr10	124251098	(C/T)	0.82	G=T,A=C	0.0%	C	0.91	??++?+??	5.94E-05	1.49 (1.22-1.8)	+	+					
			rs2300434	chr10	124251697	(T/C)	0.97	G=C,A=T	3.6%	C	0.11	--------	6.23E-08	0.7 (0.61-0.79)	+						
			rs2300435	chr10	124252279	(G/A)	0.82	G=A,A=G	1.4%	G	0.91	++++++++	1.64E-07	1.48 (1.28-1.71)	+						
			rs2300436	chr10	124252285	(C/T)	0.82	G=T,A=C	1.4%	C	0.91	++++++++	1.62E-07	1.48 (1.28-1.71)	+						
			rs2268353	chr10	124254061	(C/T)	0.98	G=T,A=C	0.0%	C	0.89	++?+++++	1.24E-05	1.4 (1.2-1.63)	+						
			rs2246731	chr10	124254180	(G/A)	0.99	G=G,A=A	1.7%	G	0.11	------?-	1.49E-07	0.7 (0.62-0.8)	+						
			rs11200654	chr10	124254343	(C/T)	0.98	G=T,A=C	4.2%	C	0.89	++++++++	5.11E-08	1.44 (1.26-1.64)	+						
			rs3106650	chr10	124254360	(G/T)	0.99	G=G,A=T	1.0%	G	0.11	------?-	2.57E-07	0.71 (0.62-0.81)	+					+	
			rs10887154	chr10	124255340	(T/C)	0.82	G=C,A=T	1.4%	C	0.09	--------	1.61E-07	0.68 (0.59-0.78)	+	+					
			rs10887155	chr10	124255686	(G/T)	0.82	G=T,A=G	1.2%	G	0.91	++++++++	1.82E-07	1.47 (1.27-1.7)	+						
		*	rs736960	chr10	124258136	(C/T)	0.72	G=C,A=T	0.9%	C	0.10	------?-	2.84E-07	0.69 (0.6-0.8)	+	+	+		+		
		*	rs2736922	chr10	124258377	(C/T)	0.99	G=C,A=T	1.0%	C	0.11	------?-	2.51E-07	0.71 (0.62-0.81)	+	+					
			rs2672607	chr10	124258758	(A/T)	0.99	G=A,A=T	1.0%	T	0.89	++++++?+	2.56E-07	1.41 (1.24-1.61)	+	+					
			rs2247430	chr10	124259750	(C/T)	0.99	G=C,A=T	1.4%	C	0.11	------?-	1.79E-07	0.7 (0.62-0.8)	+						
			rs11200655	chr10	124260564	(C/G)	0.82	G=G,A=C	1.3%	G	0.09	--------	1.75E-07	0.68 (0.59-0.78)	+						
			rs2247541	chr10	124260739	(C/T)	0.99	G=C,A=T	2.1%	C	0.12	------?-	1.17E-07	0.7 (0.61-0.8)	+						
			rs2250511	chr10	124262419	(A/G)	0.99	G=A,A=G	1.4%	G	0.89	++++++?+	1.79E-07	1.42 (1.25-1.62)	+						
			rs2142309	chr10	124263692	(T/C)	0.98	G=T,A=C	2.1%	C	0.88	++++++?+	1.14E-07	1.43 (1.25-1.63)	+						
			rs1009326	chr10	124263873	(C/T)	0.81	G=T,A=C	1.6%	C	0.91	++++++++	1.38E-07	1.48 (1.28-1.72)	+						
			rs1009325	chr10	124263877	(A/G)	0.98	G=A,A=G	2.8%	G	0.88	++++++?+	8.53E-08	1.43 (1.26-1.64)	+						
			rs909290	chr10	124264032	(A/G)	0.97	G=A,A=G	3.7%	G	0.88	++++++?+	6.16E-08	1.44 (1.26-1.65)	+	+	+				

			rs17103659	chr10	124274603	(T/C)	0.79	G=C,A=T	1.4%	C	0.09	--------	1.55E-07	0.67 (0.58-0.78)					+	
			rs142773245	chr10	124274615	(-/T)	0.79	G=T,A=-	na	na	na	na	na	na					+	
			rs714989	chr10	124280725	(A/G)	0.53	G=G,A=A	4.2%	G	0.18	------+-	6.15E-08	0.74 (0.67-0.83)			+			
**9**	***SESN3***	*	**rs11021221**	**chr11**	**95308854**	**(T/A)**	**-**	**-**	**91.2%**	**T**	**0.83**	**--------**	**4.11E-15**	**0.68 (0.62-0.75)**	+	+				
			rs1255528	chr11	95299962	(G/A)	0.78	T=A,A=G	0.1%	G	0.20	++++++++	4.89E-12	1.38 (1.26-1.52)	+					
**10**	***LRP1***	*	**rs11172113**	**chr12**	**57527283**	**(T/C)**	**-**	**-**	**99.9%**	**C**	**0.38**	**--------**	**9.03E-31**	**0.62 (0.57-0.67)**	+	+				
			rs4759275	chr12	57525756	(G/A)	0.76	T=G,C=A	0.0%	G	0.60	++++++++	4.56E-21	1.47 (1.36-1.59)	+					
			rs4759276	chr12	57526646	(G/A)	0.85	T=G,C=A	0.0%	G	0.64	++++++++	6.16E-26	1.57 (1.44-1.7)	+					
**11**	***ATP2B1***		**rs1689040**	**chr12**	**89978233**	**(C/T)**	**-**	**-**	**23.5%**	**C**	**0.59**	**+++++++-**	**7.04E-10**	**1.28 (1.18-1.39)**						
**12**	***COL4A1***		**rs7326444**	**chr13**	**110838236**	**(G/A)**	**-**	**-**	**11.7%**	**G**	**0.64**	**++++++++**	**1.02E-10**	**1.31 (1.21-1.42)**						
			rs3783111	chr13	110835049	(C/T)	0.98	G=C,A=T	6.3%	C	0.65	++++++++	2.12E-10	1.31 (1.2-1.42)	+					
			rs2305080	chr13	110838703	(T/C)	1.00	G=T,A=C	6.5%	C	0.36	--------	2.00E-10	0.77 (0.71-0.83)	+					
			rs9521638	chr13	110839428	(A/G)	0.99	G=A,A=G	0.0%	G	0.36	--?-----	1.93E-07	0.78 (0.71-0.85)	+					
		*	rs2391817	chr13	110840412	(A/T)	0.97	G=A,A=T	4.5%	T	0.36	--------	3.01E-10	0.77 (0.71-0.83)	+	+				
			rs1977892	chr13	110840624	(G/T)	0.97	G=G,A=T	6.1%	G	0.65	++++++++	2.16E-10	1.31 (1.2-1.42)	+	+				
			rs1977893	chr13	110840860	(T/C)	0.97	G=T,A=C	0.0%	C	0.36	--?-----	4.22E-08	0.77 (0.7-0.84)	+	+				
			rs3783107	chr13	110842102	(G/A)	0.89	G=G,A=A	0.2%	G	0.63	++++++++	9.42E-09	1.27 (1.17-1.38)	+					
			rs3783106	chr13	110842153	(T/G)	0.88	G=G,A=T	0.0%	G	0.63	++?+++++	1.02E-05	1.24 (1.12-1.36)	+					
			rs3783105	chr13	110842158	(T/A)	0.88	G=A,A=T	0.0%	T	0.37	--------	1.22E-07	0.8 (0.74-0.87)	+					
	COL4A2	*	**rs11838776**	**chr13**	**111040681**	**(G/A)**	**-**	**-**	**78.2%**	**G**	**0.73**	**+++++++-**	**2.46E-18**	**1.5 (1.37-1.65)**	+	+	+			
			rs9583489	chr13	111032833	(C/T)	0.82	G=C,A=T	0.0%	C	0.73	++?++++-	7.43E-12	1.44 (1.3-1.6)	+					
			rs2391825	chr13	111035483	(G/A)	0.83	G=G,A=A	0.0%	G	0.73	+++++++-	6.45E-14	1.41 (1.29-1.55)	+					
			rs80281875	chr13	111038374	(T/C)	0.83	G=T,A=C	NA	NA	NA	NA	NA	#VALUE!	+					
			rs75345389	chr13	111038379	(T/C)	0.83	G=T,A=C	0.0%	C	0.26	----?-?+	4.09E-13	0.7 (0.63-0.77)	+					
		*	rs9515201	chr13	111040798	(A/C)	0.90	G=C,A=A	2.6%	C	0.71	++++-++-	9.31E-17	1.45 (1.33-1.59)	+	+				
			rs4773174	chr13	111041530	(C/T)	0.78	G=C,A=T	0.0%	C	0.77	+++++++-	1.45E-12	1.42 (1.29-1.56)	+					
			rs55940034	chr13	111043309	(A/G)	0.94	G=A,A=G	19.2%	G	0.28	-------+	1.17E-17	0.68 (0.62-0.74)	+					
**13**	***FBN1***		**rs7174973**	**chr15**	**48763754**	**(A/G)**	**-**	**-**	**99.8%**	**G**	**0.11**	**++++++++**	**1.60E-13**	**1.54 (1.37-1.72)**						
			rs61999107	chr15	48691494	(C/T)	0.91	A=C,G=T	0.0%	C	0.89	--?---?-	0.0008402	0.79 (0.69-0.91)	+					
			rs2015637	chr15	48716853	(T/C)	0.96	A=T,G=C	0.0%	C	0.11	++++++?+	3.07E-09	1.42 (1.27-1.6)	+					
			rs75225368	chr15	48785206	(C/T)	0.94	A=C,G=T	0.0%	C	0.90	?-------	2.44E-07	0.71 (0.62-0.81)	+					
			rs8026752	chr15	48823770	(A/G)	0.90	A=A,G=G	0.0%	G	0.10	?+++++++	3.39E-07	1.4 (1.23-1.6)	+		+			
			rs595244	chr15	48840835	(C/T)	0.89	A=C,G=T	0.0%	C	0.89	--------	1.57E-09	0.7 (0.62-0.79)	+					
		*	rs1561207	chr15	48858971	(G/T)	0.90	A=G,G=T	0.0%	G	0.89	--------	2.41E-09	0.7 (0.62-0.79)	+	+				
		*	rs2437947	chr15	48876881	(T/A)	0.90	A=T,G=A	0.0%	T	0.89	--------	2.94E-09	0.7 (0.63-0.79)		+	+			
			rs686861	chr15	48885005	(A/G)	0.89	A=A,G=G	0.0%	G	0.11	++++++++	4.66E-09	1.41 (1.26-1.59)		+				
			rs2456475	chr15	48885877	(G/A)	0.89	A=G,G=A	0.0%	G	0.89	--------	6.30E-09	0.71 (0.63-0.8)	+					
			rs2455925	chr15	48893649	(T/C)	0.89	A=T,G=C	0.0%	C	0.11	++++++++	6.84E-09	1.41 (1.25-1.58)	+					
			rs591519	chr15	48894200	(A/T)	0.89	A=A,G=T	0.0%	T	0.11	++++++++	6.06E-09	1.41 (1.26-1.58)	+					
			rs1036476	chr15	48914775	(T/C)	0.85	A=T,G=C	0.0%	C	0.11	++++++++	8.61E-09	1.41 (1.25-1.58)					+	
			rs689304	chr15	48922360	(C/T)	0.87	A=C,G=T	0.0%	C	0.89	--------	9.67E-09	0.71 (0.63-0.8)	+					
		*	rs4775769	chr15	48939888	(T/G)	0.82	A=G,G=T	0.0%	G	0.89	---?-??-	6.12E-07	0.7 (0.61-0.8)	+					
**14**	***THSD4***		**rs10851839**	**chr15**	**71628370**	**(T/A)**	**-**	**-**	**30.4%**	**T**	**0.32**	**------+-**	**5.51E-11**	**0.75 (0.69-0.82)**						
			rs3743111	chr15	71587373	(G/A)	0.70	T=G,A=A	0.0%	G	0.39	--?-----	2.06E-06	0.8 (0.73-0.88)				+		
		*	rs11853359	chr15	71621524	(G/A)	0.96	T=A,A=G	0.1%	G	0.67	++?+++-+	2.96E-08	1.31 (1.19-1.44)	+	+	+	+	+	
**15**	***MRPS6/SLC5A3 /KCNE2***	*	**rs28451064**	**chr21**	**35593827**	**(G/A)**	**-**	**-**	**99.9%**	**G**	**0.88**	**+++++++**	**1.16E-22**	**2.04 (1.77-2.35)**	+	+				
			rs9305545	chr21	35595821	(A/G)	0.82	G=A,A=G	0.0%	G	0.15	--?-----	1.16E-15	0.56 (0.49-0.65)						+
			rs9982601	chr21	35599128	(C/T)	0.78	G=C,A=T	0.0%	C	0.87	+++++++-	9.58E-17	1.75 (1.53-1.99)	+					
			rs9980618	chr21	35600505	(C/T)	0.78	G=C,A=T	0.0%	C	0.87	+++++++-	7.18E-17	1.76 (1.54-2)	+					

**16**	***TIMP3***		**rs137507**	**chr22**	**33282971**	**(T/C)**	**-**	**-**	**17.7%**	**C**	**0.89**	**-----+--**	**3.30E-08**	**0.73 (0.65-0.81)**				
			rs137498	chr22	33276313	(C/G)	0.19	T=C,C=G	2.5%	G	0.56	--------	3.32E-07	0.82 (0.76-0.88)				+
			rs137500	chr22	33276686	(A/G)	0.19	T=A,C=G	2.4%	G	0.56	--------	3.57E-07	0.82 (0.76-0.88)	+			
		*	rs4448	chr22	33276999	(G/A)	0.19	T=G,C=A	2.5%	G	0.44	++++++++	3.27E-07	1.22 (1.13-1.32)	+	+	+	+
		*	rs137503	chr22	33277200	(C/G)	0.19	T=C,C=G	2.0%	G	0.56	--------	4.21E-07	0.82 (0.76-0.89)	+	+	+	+
			rs4452	chr22	33283257	(T/C)	1.00	T=T,C=C	4.5%	C	0.89	-----+--	1.57E-07	0.74 (0.66-0.83)	+			
			rs137509	chr22	33284221	(T/G)	0.90	T=T,C=G	0.2%	G	0.90	-----+--	5.02E-06	0.76 (0.67-0.85)	+			
			rs80693	chr22	33300347	(G/T)	0.98	T=G,C=T	4.3%	G	0.12	+++++-++	1.62E-07	1.36 (1.21-1.52)		+		

Supplementary Table 6: Colocalization of association with SCAD and eQTL association.
We display the results of approximate Bayes factor colocalization analysis for the association with SCAD, on one hand, eQTL association with the indicated eGenes, on the other hand. Best eQTL P-value : Unadjusted P-values of association between SNP and gene expression reported from GTEx (v8 release). NSNPs: number of SNPs used for he analysis. All SNPs present in both studies in a 2Mb window centered on SCAD lead variant were used. H0-H4: Posterior probability that: H0: neither trait has a genetic association in the region; H1: only trait 1 has a genetic association in the region; H2: only trait 2 has a genetic association in the region; H3: both traits are associated, but with different causal variants; H4: both traits are associated and share a single causal variant.

Locus	SCAD lead SNP	eGene	Tissue	SCAD lead SNP eQTL P-value	Best eQTL SNP	Best eQTL P-value	NSNPs	H0	H1	H2	H3	H4
1	rs34370185	*FGGY-DT*	Artery_Coronary	9.6E-16	rs7543389	1.3E-19	1780	0%	0%	0%	9%	91%
		FGGY-DT	Artery_Tibial	1.4E-61	rs7543389	1.5E-69	1780	0%	0%	0%	9%	91%
		FGGY-DT	Artery_Aorta	6.6E-53	rs7543389	6.0E-59	1780	0%	0%	0%	9%	91%
2	rs1146473	*F3*	Artery_Tibial	3.8E-12	rs60897247	2.8E-16	2317	0%	0%	0%	100%	0%
		F3	Artery_Aorta	1.3E-04	rs1612481	3.6E-08	2317	0%	0%	0%	6%	94%
3	rs4970935	*ECM1*	Cells_Cultured_fibroblasts	3.8E-20	rs11801255	5.2E-25	1555	0%	0%	0%	100%	0%
		ECM1	Artery_Aorta	1.7E-07	rs6693567	5.4E-11	1555	0%	0%	0%	0%	100%
		ADAMTSL4	Cells_Cultured_fibroblasts	2.0E-28	rs6693567	1.5E-34	1555	0%	0%	0%	100%	0%
		ADAMTSL4	Artery_Tibial	8.9E-24	rs6693567	5.9E-29	1555	0%	0%	0%	51%	49%
		ADAMTSL4	Artery_Aorta	4.5E-18	rs6693567	1.7E-22	1555	0%	0%	0%	72%	28%
		HORMAD1	Cells_Cultured_fibroblasts	3.2E-73	rs7521898	1.2E-82	1555	0%	0%	0%	100%	0%
		CTSS	Artery_Tibial	1.5E-24	rs41271951	5.5E-30	1555	0%	0%	0%	100%	0%
		ADAMTSL4-AS2	Artery_Aorta	1.1E-17	rs11204664	2.5E-22	1555	0%	0%	0%	100%	0%
		ADAMTSL4-AS2	Artery_Coronary	2.1E-10	rs11204664	4.7E-14	1555	0%	0%	0%	100%	0%
		ADAMTSL4-AS2	Artery_Tibial	6.0E-10	rs11204664	5.9E-14	1555	0%	0%	0%	100%	0%
		MRPS21	Artery_Aorta	3.2E-13	rs6693697	3.2E-17	1555	0%	0%	0%	100%	0%
		MRPS21	Artery_Tibial	8.4E-09	rs3818978	1.2E-12	1555	0%	0%	0%	97%	3%
		MRPS21	Cells_Cultured_fibroblasts	7.0E-10	rs34629240	6.6E-14	1555	0%	0%	0%	100%	0%
4	rs6828005	*AFAP1*	Whole_Blood	1.2E-145	rs56069442	6.9E-159	4147	0%	0%	0%	100%	0%
5	rs1507928	*ZNF827*	Artery_Tibial	7.2E-17	rs13128814	1.3E-21	2004	0%	0%	0%	3%	97%
		ZNF827	Artery_Aorta	3.5E-08	rs13124853	6.9E-12	2004	0%	0%	0%	6%	94%
		RP11-6L6.4	Artery_Tibial	3.7E-19	rs1865531	1.7E-23	2004	0%	0%	0%	100%	0%
6	rs73102285	*ITGA1*	Artery_Tibial	2.6E-04	rs10038615	8.5E-08	3001	0%	0%	0%	4%	96%
7	rs9349379	*PHACTR1*	Artery_Tibial	1.1E-35	rs9349379	8.0E-42	2207	0%	0%	0%	0%	100%
		PHACTR1	Artery_Aorta	5.3E-13	rs9349379	2.0E-17	2207	0%	0%	0%	0%	100%
		PHACTR1	Artery_Coronary	1.1E-05	rs9349379	3.0E-09	2207	0%	0%	0%	0%	100%
		TBC1D7	Artery_Tibial	7.8E-09	rs499818	2.1E-11	2207	0%	0%	0%	98%	2%
		GFOD1	Artery_Tibial	7.9E-12	rs7742086	7.7E-15	2041	0%	0%	0%	100%	0%
		RP1-257A7.4	Artery_Tibial	2.0E-10	rs398151	1.9E-14	2207	0%	0%	0%	100%	0%
9	rs11021221	*SESN3*	Artery_Tibial	6.8E-11	rs11021221	5.4E-15	2289	0%	0%	0%	0%	100%
		SESN3	Artery_Coronary	8.0E-06	rs10831371	2.5E-09	2288	0%	0%	0%	88%	12%
		SESN3	Artery_Aorta	1.1E-07	rs11021233	2.7E-11	2289	0%	0%	0%	0%	100%
10	rs11172113	*LRP1*	Artery_Aorta	2.7E-11	rs11172113	3.6E-15	1371	0%	0%	0%	0%	100%
		LRP1	Artery_Tibial	1.9E-16	rs11172113	9.4E-21	1371	0%	0%	0%	0%	100%
11	rs1689040	*ATP2B1*	Artery_Tibial	1.7E-14	rs2681472	1.6E-18	1682	0%	0%	0%	12%	88%
12	rs11838776	*COL4A1*	Cells_Cultured_fibroblasts	3.2E-19	rs1927342	6.6E-24	1941	0%	0%	0%	100%	0%
13	rs7174973	*FBN1*	Whole_Blood	4.2E-34	rs924816	6.2E-40	1243	0%	0%	0%	100%	0%
		RP11-227D13.1	Artery_Aorta	8.7E-05	rs55694948	3.1E-08	1243	0%	1%	0%	14%	85%
		RP11-227D13.1	Artery_Tibial	9.3E-08	rs6493333	2.4E-11	1243	0%	0%	0%	100%	0%
		RP11-227D13.1	Cells_Cultured_fibroblasts	7.4E-32	rs6493333	7.4E-38	1243	0%	0%	0%	100%	0%
14	rs10851839	*THSD4*	Artery_Aorta	2.6E-08	rs11853359	4.0E-12	2075	0%	0%	0%	2%	98%
		NR2E3	Artery_Aorta	2.8E-02	rs34719283	1.6E-05	2075	0%	5%	0%	4%	92%
15	rs28451064	*KCNE2*	Artery_Aorta	1.9E-04	rs9980618	5.9E-08	1992	0%	0%	0%	1%	99%
		SLC5A3	Artery_Tibial	1.0E-03	rs8128536	3.0E-07	1992	0%	1%	0%	25%	74%
		SLC5A3	Artery_Aorta	1.1E-05	rs28451064	3.2E-09	1992	0%	0%	0%	0%	100%
		AP000318.2	Artery_Aorta	1.8E-04	rs80284318	4.6E-08	1992	0%	0%	0%	1%	98%
		LINC00310	Artery_Aorta	7.0E-06	rs9305545	1.3E-09	1992	0%	0%	0%	4%	96%
		MRPS6	Artery_Aorta	6.9E-07	rs28451064	1.2E-10	1992	0%	0%	0%	0%	100%
		MRPS6	Artery_Tibial	2.7E-03	rs1018757	2.0E-06	1992	0%	1%	0%	10%	89%

Supplementary Table 7: Transcriptome-wide associated genes with SCAD in 3 artery tissues (GTEx v8 gene expression models).
Genes with TWAS FDR < 0.05 are shown. Chr: chromosome, P-value: Unadjusted TWAS P-values obtained from two-sided Z-score test, P.Bonf : Bonferroni corrected P-value. FDR: False Discovery Rate

					Best GWAS SNP		Best eQTL SNP		TWAS			
**Gene**	**Tissue**	**Chr**	**Start**	**End**	**rsID**	**Z-Score**	**rsID**	**Z-Score**	**Z-score**	**P-value**	**P.Bonf**	**FDR**
ADAMTSL4	Cells_Transformed_fibroblasts	1	150521884	150533413	rs834238	11.8	rs6693567	-8.0	-11.7	2.E-31	5.E-27	5.E-27
LRP1	Artery_Tibial	12	57522276	57607134	rs11172113	-11.5	rs11172113	-8.2	11.5	9.E-31	2.E-26	1.E-26
ADAMTSL4	Artery_Tibial	1	150521884	150533413	rs834238	11.8	rs6693567	-7.6	-11.4	3.E-30	8.E-26	3.E-26
ADAMTSL4	Artery_Aorta	1	150521884	150533413	rs834238	11.8	rs6693567	-7.1	-11.4	5.E-30	1.E-25	3.E-26
PHACTR1	Artery_Aorta	6	12717893	13288645	rs9349379	-11.8	rs9349379	-6.2	11.2	6.E-29	2.E-24	3.E-25
PHACTR1	Artery_Tibial	6	12717893	13288645	rs9349379	-11.8	rs9349379	-9.2	10.0	2.E-23	6.E-19	1.E-19
ECM1	Artery_Aorta	1	150480538	150486265	rs834238	11.8	rs6693567	-3.9	-9.2	5.E-20	1.E-15	2.E-16
ADAMTSL4-AS2	Artery_Aorta	1	150521040	150530200	rs834238	11.8	rs6693567	-4.9	-8.7	4.E-18	1.E-13	1.E-14
ADAMTSL4-AS2	Artery_Coronary	1	150521040	150530200	rs834238	11.8	rs6655975	-3.9	-7.4	1.E-13	3.E-09	4.E-10
ECM1	Cells_Transformed_fibroblasts	1	150480538	150486265	rs834238	11.8	rs10888585	6.8	-7.1	2.E-12	5.E-08	5.E-09
C1orf54	Artery_Coronary	1	150240967	150253327	rs834238	11.8	rs1932934	3.6	7.0	3.E-12	8.E-08	7.E-09
SESN3	Artery_Aorta	11	94898704	94965705	rs2186739	7.2	rs11021233	-5.4	-6.4	1.E-10	3.E-06	3.E-07
APH1A	Whole_Blood	1	150237804	150241980	rs834238	11.8	rs2275780	-3.6	-6.2	5.E-10	1.E-05	1.E-06
FGGY-DT	Artery_Aorta	1	59597608	59664293	rs12739770	7.1	rs12758288	11.0	6.0	2.E-09	5.E-05	4.E-06
FGGY-DT	Artery_Coronary	1	59597608	59664293	rs12739770	7.1	rs7543389	6.5	6.0	3.E-09	7.E-05	5.E-06
SESN3	Artery_Tibial	11	94898704	94965705	rs2186739	7.2	rs11021233	-7.4	-5.8	8.E-09	2.E-04	1.E-05
ADAMTSL4-AS2	Artery_Tibial	1	150521040	150530200	rs834238	11.8	rs9659073	3.4	-5.8	8.E-09	2.E-04	1.E-05
FGGY-DT	Artery_Tibial	1	59597608	59664293	rs12739770	7.1	rs12758643	12.6	5.4	9.E-08	2.E-03	1.E-04
C1orf54	Artery_Aorta	1	150240967	150253327	rs834238	11.8	rs751931	-4.9	5.0	6.E-07	2.E-02	9.E-04
F3	Artery_Aorta	1	94994781	95007356	rs1146474	5.7	rs1493276	-3.8	-4.9	1.E-06	3.E-02	1.E-03
MRPS6	Artery_Aorta	21	35496253	35515334	rs9305545	-8.0	rs12627001	3.9	-4.9	1.E-06	3.E-02	2.E-03
MRPS6	Artery_Tibial	21	35496253	35515334	rs9305545	-8.0	rs1018757	5.0	-4.8	2.E-06	5.E-02	2.E-03
ZNF827	Artery_Tibial	4	146678779	146859787	rs1507928	5.8	rs1979974	7.5	4.7	3.E-06	8.E-02	3.E-03
LTBP3	Whole_Blood	11	65306276	65326401	rs1783541	4.9	rs17146964	5.8	4.6	4.E-06	1.E-01	5.E-03
PHACTR1	Cells_Transformed_fibroblasts	6	12717893	13288645	rs9349379	-11.8	rs7757858	4.5	4.6	4.E-06	1.E-01	5.E-03
COL4A1	Cells_Transformed_fibroblasts	13	110801318	110959496	rs9515201	-8.3	rs7991842	-6.8	4.6	5.E-06	1.E-01	5.E-03
NT5DC1	Artery_Aorta	6	116422012	116570660	rs12208531	-4.4	rs550480	7.0	-4.5	8.E-06	2.E-01	7.E-03
ATP2B1	Artery_Tibial	12	89981828	90102608	rs6538195	-5.7	rs2681492	7.1	-4.5	8.E-06	2.E-01	7.E-03
CDC42SE1	Cells_Transformed_fibroblasts	1	151023897	151042801	rs3820539	5.5	rs4970998	4.7	-4.5	8.E-06	2.E-01	7.E-03
RPRD2	Whole_Blood	1	150335567	150449042	rs834238	11.8	rs486275	-4.1	4.4	1.E-05	3.E-01	9.E-03
ANKRD42	Artery_Tibial	11	82904837	82967141	rs17519683	-4.7	rs565674	9.3	-4.4	1.E-05	3.E-01	9.E-03
GGCX	Whole_Blood	2	85772404	85788670	rs6705971	-4.6	rs10198569	10.2	-4.4	1.E-05	4.E-01	1.E-02
ANKRD42	Artery_Aorta	11	82904837	82967141	rs17519683	-4.7	rs647644	5.4	-4.3	1.E-05	4.E-01	1.E-02
FRK	Artery_Coronary	6	116252312	116381921	rs12208531	-4.4	rs195529	4.4	-4.3	2.E-05	4.E-01	1.E-02
TARS2	Artery_Tibial	1	150459887	150480078	rs834238	11.8	rs13294	-5.3	4.3	2.E-05	5.E-01	1.E-02
CCDC90B	Artery_Tibial	11	82970139	82997170	rs17519683	-4.7	rs6592108	14.5	-4.3	2.E-05	5.E-01	1.E-02
AGBL2	Artery_Aorta	11	47681143	47736941	rs1377416	4.5	rs11828339	3.9	-4.2	2.E-05	6.E-01	2.E-02
CCDC90B	Artery_Aorta	11	82970139	82997170	rs17519683	-4.7	rs647644	11.4	-4.2	2.E-05	6.E-01	2.E-02
TBC1D7	Artery_Tibial	6	13266774	13328815	rs9349379	-11.8	rs2458307	6.3	4.2	3.E-05	8.E-01	2.E-02
MIA3	Artery_Aorta	1	222791428	222841354	rs17163303	4.0	rs2133189	3.9	4.2	3.E-05	8.E-01	2.E-02
SLC24A3	Artery_Aorta	20	19193290	19703581	rs4813361	4.5	rs6046169	-5.8	-4.2	3.E-05	9.E-01	2.E-02
GGCX	Artery_Tibial	2	85772404	85788670	rs6705971	-4.6	rs10187424	9.3	-4.1	3.E-05	9.E-01	2.E-02
CCDC90B	Artery_Coronary	11	82970139	82997170	rs17519683	-4.7	rs7111383	7.5	-4.1	4.E-05	1.E+00	2.E-02
GJA1	Artery_Tibial	6	121756838	121770873	rs10499112	3.8	rs9490305	5.4	4.1	4.E-05	1.E+00	2.E-02
RAPGEF6	Artery_Aorta	5	130759614	130970929	rs924434	3.7	rs6873582	4.3	4.1	5.E-05	1.E+00	3.E-02
MAT2A	Artery_Tibial	2	85766288	85771845	rs6705971	-4.6	rs3821021	4.6	-4.1	5.E-05	1.E+00	3.E-02
COL4A2	Cells_Transformed_fibroblasts	13	110958159	111165374	rs9515201	-8.3	rs7991842	-6.1	4.0	6.E-05	1.E+00	3.E-02
INO80E	Whole_Blood	16	30006615	30016829	rs3809624	5.1	rs4787491	5.5	4.0	6.E-05	1.E+00	3.E-02
SLC24A3	Artery_Tibial	20	19193290	19703581	rs4813361	4.5	rs3790227	-5.9	-4.0	7.E-05	1.E+00	4.E-02
GGCX	Artery_Coronary	2	85772404	85788670	rs6705971	-4.6	rs2886722	4.0	-4.0	7.E-05	1.E+00	4.E-02
LRCH1	Artery_Aorta	13	47127303	47327175	rs17282423	4.1	rs7982810	5.0	3.9	9.E-05	1.E+00	5.E-02

Supplementary Table 8: Druggable genome lookup

We display genes annotated as potential targets in SCAD genetic risk loci and which are part of the set of genes encoding druggable targets derived by Finan et al. using ChEMBL v17. "Priority" column indicates the position in the drug-development pipeline: targets of approved agents and clinical-phase drug candidates (Tier 1); genes encoding targets with known bioactive drug-like small molecule binding partners and those with substantial sequence) with approved drug targets (Tier 2); and genes encoding other secreted or extracellular proteins, proteins with more distant similarity to approved drug targets, and members of key druggable gene families (Tiers 3 and 4). "Compounds" column indicates the number of compounds with any assay data in ChEMBL against the target. "Drug mechanim compounds" column indicates The number of compounds that have an entry in the ChEMBL drug_mechanism table against the target. This will be drugs that have an indication target pair and the targets can be thought of as efficacy targets for these compounds.

Locus			Druggable genome lookup				ChEMBL compounds								
**Locus**	**SCAD lead SNP**	**Annotated gene**	**Uniprot ID**	**Protein description**	**Priority**	**Drug type**	**Target Chembl ID**	**Target Type**	**Target Name**	**Compounds**	**Drug mechanism compounds**	**Phase I**	**Phase II**	**Phase III**	**Phase IV**
1	rs34370185	*FGGY-DT*													
2	rs1146473	*F3*	P13726	coagulation factor III (thromboplastin, tissue factor)	**1**	BIO_MOL | SMALL_MOL	CHEMBL4081	SINGLE PROTEIN	Coagulation factor III	201	1	3	2	2	1
							CHEMBL2095194	PROTEIN COMPLEX	Coagulation factor VII/tissue factor	623	0	0	3	1	3
3	rs4970935	*ECM1*	Q16610	extracellular matrix protein 1	**4**	BIO_MOL									
		ADAMTSL4	Q6UY14	ADAMTS-like 4	**4**	BIO_MOL									
4	rs6828005	*AFAP1*													
5	rs1507928	*ZNF827*													
6	rs73102285	*ITGA1*	P56199	integrin, alpha 1	**3**	BIO_MOL	CHEMBL3682	SINGLE PROTEIN	Integrin alpha-1	20	0	0	0	0	0
							CHEMBL3137278	PROTEIN COMPLEX	Integrin alpha1/beta1 complex	6	0	0	0	0	0
7	rs9349379	*PHACTR1*													
8	rs2736923	*HTRA1*	Q92743	HtrA serine peptidase 1	**3**	BIO_MOL	CHEMBL4523419	SINGLE PROTEIN	Serine protease HTRA1	187	0	0	0	0	0
9	rs11021221	*SESN3*													
10	rs11172113	*LRP1*	Q07954	low density lipoprotein receptor-related protein 1	**3**	BIO_MOL	CHEMBL4630884	SINGLE PROTEIN	Prolow-density lipoprotein receptor-related protein 1	0	1	0	0	0	0
11	rs1689040	*ATP2B1*													
12	rs7326444	*COL4A1*	P02462	collagen, type IV, alpha 1	**3**	BIO_MOL	CHEMBL2364188	PROTEIN COMPLEX GROUP	Collagen	0	2	0	0	0	0
	rs11838776	*COL4A2*	P08572	collagen, type IV, alpha 2	**3**	BIO_MOL	CHEMBL2364188	PROTEIN COMPLEX GROUP	Collagen	0	2	0	0	0	0
13	rs7174973	*FBN1*	P35555	fibrillin 1	**3**	BIO_MOL									
14	rs10851839	*THSD4*	Q6ZMP0	thrombospondin, type I, domain containing 4	**3**	BIO_MOL									
15	rs28451064	*SLC5A3*	P53794	solute carrier family 5 (sodium/myo-inositol cotransporter), member 3	**2**	SMALL_MOL									
		MRPS6													
16	rs137507	*KCNE2*													
		TIMP3	P35625	TIMP metallopeptidase inhibitor 3	**4**	BIO_MOL									

Supplementary Table 9: Colocalization of association with SCAD and five other traits at SCAD top loci

We display the results of approximate Bayes factor colocalization analysis for the association with SCAD, on one hand, and 14 different traits on the other hand.

Top SNP (SCAD): SNP with lowest P-value for SCAD association in the set of common variants between the two studies. rsID: SNP ID, EA: Effect Allele. OA: Other Allele. Z: Z-score of the indicated top SNP, P: Unadjusted P-values of association obtained from two-sided Wald test. NSNPs: number of SNPs used for the analysis. R2: R2 of linkage disequilibrium between top SCAD SNP and top Trait SNP in the European population of 1000 Genomes reference panel PP.H0-H4.abf: Posterior probability that: H0: neither trait has a genetic association in the region; H1: only trait 1 has a genetic association in the region; H2: only trait 2 has a genetic association in the region; H3: both traits are associated, but with different causal variants; H4: both traits are associated and share a single causal variant.

			Top SCAD SNP							Top Trait SNP													
**Locus**	**Candidate Genes**	**Trait**	**rsID**	**EA**	**OA**	**SCAD Z**	**SCAD P**	**Trait Z**	**Trait P**	**rsID**	**EA**	**OA**	**SCAD Z**	**SCAD P**	**Trait Z**	**Trait P**	**R2**	**NSNPs**	**PP.H0.abf**	**PP.H1.abf**	**PP.H2.abf**	**PP.H3.abf**	**PP.H4.abf**
1	*FGGY-DT*	Intracranial Aneurysm (IA)	rs34370185	T	G	7.09	1.4E-12	-1.0	3.0E-01	rs61292499	A	G	-0.06	9.5E-01	-2.4	1.7E-02	0.00	872	0%	96%	0%	2%	2%
2	*F3*	Intracranial Aneurysm (IA)	rs1146473	C	T	5.82	5.8E-09	1.8	7.6E-02	rs112663538	T	C	-1.53	1.3E-01	2.6	9.0E-03	0.00	1288	0%	90%	0%	4%	6%
3	*ECM1/ADAMTSL4*	Intracranial Aneurysm (IA)	rs4970935	T	C	-13.04	6.1E-39	0.9	3.5E-01	rs17599629	G	A	-1.89	5.8E-02	-2.6	1.0E-02	0.02	831	0%	96%	0%	2%	2%
4	*AFAP1*	Intracranial Aneurysm (IA)	rs6828005	A	G	-6.67	2.6E-11	-3.0	2.4E-03	rs11724720	T	C	-5.45	5.4E-08	-4.1	4.2E-05	0.61	1634	0%	55%	0%	19%	26%
5	*ZNF827*	Intracranial Aneurysm (IA)	rs1507928	C	T	5.75	8.9E-09	-1.2	2.4E-01	rs28626105	C	T	4.99	6.2E-07	-3.6	2.9E-04	0.48	1075	0%	83%	0%	7%	10%
6	*ITGA1*	Intracranial Aneurysm (IA)	rs73102285	G	A	5.72	1.1E-08	2.1	3.3E-02	rs1862191	T	C	0.21	8.3E-01	3.6	3.5E-04	0.00	1743	0%	81%	0%	10%	9%
7	*PHACTR1*	Intracranial Aneurysm (IA)	rs6925904	G	A	-7.50	6.3E-14	-0.9	3.7E-01	rs2876277	G	A	0.19	8.5E-01	-3.1	1.9E-03	0.00	1368	0%	93%	0%	6%	2%
8	*HTRA1*	Intracranial Aneurysm (IA)	rs2736923	A	G	5.47	4.6E-08	1.5	1.4E-01	rs74765115	G	T	-2.97	2.9E-03	-3.8	1.3E-04	0.01	1152	0%	70%	0%	23%	6%
9	*SESN3*	Intracranial Aneurysm (IA)	rs2186739	C	T	-7.23	4.8E-13	0.3	7.4E-01	rs555556	G	T	-1.49	1.4E-01	-2.3	2.3E-02	0.00	1148	0%	96%	0%	3%	1%
10	*LRP1*	Intracranial Aneurysm (IA)	rs11172113	C	T	-11.53	9.0E-31	-0.1	9.0E-01	rs147304335	A	G	0.45	6.5E-01	3.2	1.4E-03	0.00	823	0%	95%	0%	4%	1%
11	*ATP2B1*	Intracranial Aneurysm (IA)	rs1689040	T	C	-6.17	7.0E-10	-2.2	2.6E-02	rs1590008	T	C	2.44	1.5E-02	3.4	5.7E-04	0.31	852	0%	71%	0%	6%	23%
12	*COL4A1*	Intracranial Aneurysm (IA)	rs7326444	A	G	-6.46	1.0E-10	0.2	8.4E-01	rs11619453	A	C	0.75	4.5E-01	-2.2	2.5E-02	0.01	896	0%	97%	0%	2%	1%
12	*COL4A2*	Intracranial Aneurysm (IA)	rs11838776	A	G	-8.74	2.5E-18	-1.4	1.6E-01	rs147233316	A	C	0.45	6.5E-01	-3.3	1.0E-03	0.00	1012	0%	89%	0%	7%	4%
13	*FBN1*	Intracranial Aneurysm (IA)	rs655015	C	T	5.87	4.3E-09	0.8	4.0E-01	rs12909029	T	C	0.88	3.8E-01	3.3	1.1E-03	0.04	411	0%	95%	0%	3%	2%
14	*THSD4*	Intracranial Aneurysm (IA)	rs28398466	A	G	-6.46	1.1E-10	-1.5	1.4E-01	rs55940121	A	G	0.15	8.8E-01	-3.4	6.8E-04	0.08	922	0%	91%	0%	5%	3%
15	*MRPS6/SLC5A3/KCNE2*	Intracranial Aneurysm (IA)	rs9980618	T	C	-8.35	7.2E-17	4.4	1.1E-05	rs9977093	A	G	-7.82	5.3E-15	4.7	3.2E-06	0.88	1298	0%	1%	0%	1%	99%
16	*TIMP3*	Intracranial Aneurysm (IA)	rs137528	A	G	-5.40	6.8E-08	-0.9	3.5E-01	rs78325826	G	A	0.34	7.3E-01	-2.4	1.7E-02	0.00	1558	1%	93%	0%	4%	2%
1	*FGGY-DT*	Cervical Artery Dissection (CeAD)	rs34370185	T	G	7.09	1.4E-12	4.2	2.1E-05	rs7551554	G	A	6.47	9.6E-11	4.6	3.6E-06	0.84	998	0%	1%	0%	4%	95%
2	*F3*	Cervical Artery Dissection (CeAD)	rs1146473	C	T	5.82	5.8E-09	0.3	7.3E-01	rs17400584	A	G	-1.63	1.0E-01	-3.0	3.0E-03	0.01	1473	0%	87%	0%	10%	3%
3	*ECM1/ADAMTSL4*	Cervical Artery Dissection (CeAD)	rs1260387	C	T	-12.21	3.2E-34	-3.2	1.4E-03	rs59898460	C	T	-9.69	3.1E-22	-4.0	5.3E-05	0.22	970	0%	18%	0%	27%	55%
4	*AFAP1*	Cervical Artery Dissection (CeAD)	rs6828005	A	G	-6.67	2.6E-11	-0.6	5.5E-01	rs9330346	C	T	0.05	9.6E-01	3.5	5.4E-04	0.00	2028	0%	80%	0%	18%	2%
5	*ZNF827*	Cervical Artery Dissection (CeAD)	rs1507928	C	T	5.75	8.9E-09	1.5	1.2E-01	rs10017224	T	C	-0.07	9.4E-01	3.3	8.4E-04	0.00	1159	0%	86%	0%	8%	6%
6	*ITGA1*	Cervical Artery Dissection (CeAD)	rs73102285	G	A	5.72	1.1E-08	0.0	9.8E-01	rs12697099	A	T	-3.10	1.9E-03	-3.6	2.6E-04	0.00	1979	0%	85%	0%	13%	2%
7	*PHACTR1*	Cervical Artery Dissection (CeAD)	rs6925904	G	A	-7.50	6.3E-14	-2.9	4.0E-03	rs1937768	G	A	-4.53	5.8E-06	-3.7	1.8E-04	0.03	1565	0%	33%	0%	23%	44%
8	*HTRA1*	Cervical Artery Dissection (CeAD)	rs2736923	A	G	5.47	4.6E-08	0.9	3.4E-01	rs7085188	G	C	-2.15	3.1E-02	-3.4	7.6E-04	0.01	1458	0%	83%	0%	13%	4%
9	*SESN3*	Cervical Artery Dissection (CeAD)	rs11021221	A	T	7.84	4.1E-15	0.7	4.7E-01	rs11021389	A	G	-1.24	2.1E-01	2.8	4.5E-03	0.00	1260	0%	91%	0%	6%	3%
10	*LRP1*	Cervical Artery Dissection (CeAD)	rs11172113	C	T	-11.53	9.0E-31	-5.4	5.1E-08	rs11172113	C	T	-11.53	9.0E-31	-5.4	5.1E-08	1.00	951	0%	0%	0%	0%	100%
11	*ATP2B1*	Cervical Artery Dissection (CeAD)	rs1689040	T	C	-6.17	7.0E-10	0.0	9.7E-01	rs112914514	G	A	1.32	1.9E-01	2.7	6.2E-03	0.01	969	0%	92%	0%	5%	3%
12	*COL4A1*	Cervical Artery Dissection (CeAD)	rs7326444	A	G	-6.46	1.0E-10	-1.7	8.1E-02	rs12584092	T	C	0.51	6.1E-01	3.7	2.6E-04	0.00	1092	0%	76%	0%	16%	7%
12	*COL4A2*	Cervical Artery Dissection (CeAD)	rs11838776	A	G	-8.74	2.5E-18	-1.1	2.8E-01	rs34905765	T	C	-1.81	7.0E-02	2.6	9.6E-03	0.00	1159	0%	91%	0%	5%	4%
13	*FBN1*	Cervical Artery Dissection (CeAD)	rs2437947	A	T	5.94	2.9E-09	0.7	4.9E-01	rs7495591	T	A	0.39	6.9E-01	3.1	1.8E-03	0.05	506	0%	92%	0%	4%	4%
14	*THSD4*	Cervical Artery Dissection (CeAD)	rs10851839	A	T	6.56	5.5E-11	1.1	2.9E-01	rs4357892	C	A	0.20	8.4E-01	-2.8	5.7E-03	0.01	1090	0%	91%	0%	5%	3%
15	*MRPS6/SLC5A3/KCNE2*	Cervical Artery Dissection (CeAD)	rs9980618	T	C	-8.35	7.2E-17	-2.9	3.5E-03	rs9980618	T	C	-8.35	7.2E-17	-2.9	3.5E-03	1.00	1469	0%	40%	0%	3%	57%
16	*TIMP3*	Cervical Artery Dissection (CeAD)	rs137528	A	G	-5.40	6.8E-08	-0.6	5.5E-01	rs74662609	T	C	-1.75	8.0E-02	-3.2	1.6E-03	0.00	1758	1%	84%	0%	12%	4%
1	*FGGY-DT*	Diastolic Blood Pressure (DBP)	rs34370185	T	G	7.09	1.4E-12	5.0	7.0E-07	rs12730750	A	G	5.32	1.1E-07	6.0	1.9E-09	0.52	1047	0%	0%	0%	8%	91%
2	*F3*	Diastolic Blood Pressure (DBP)	rs1146473	C	T	5.82	5.8E-09	0.9	3.7E-01	rs17396055	A	G	-0.13	9.0E-01	-6.3	4.1E-10	0.01	1579	0%	0%	0%	100%	0%
3	*ECM1/ADAMTSL4*	Diastolic Blood Pressure (DBP)	rs4970935	T	C	-13.04	6.1E-39	-2.9	3.6E-03	rs35392872	A	G	0.39	6.9E-01	-3.8	1.2E-04	0.01	1035	0%	89%	0%	4%	8%
4	*AFAP1*	Diastolic Blood Pressure (DBP)	rs6828005	A	G	-6.67	2.6E-11	0.4	6.7E-01	rs7655880	G	A	-1.75	8.0E-02	4.6	4.8E-06	0.00	2213	0%	89%	0%	11%	0%
5	*ZNF827*	Diastolic Blood Pressure (DBP)	rs1507928	C	T	5.75	8.9E-09	-2.7	7.7E-03	rs10022648	G	A	1.31	1.9E-01	-5.1	3.1E-07	0.01	1247	0%	51%	0%	41%	8%
6	*ITGA1*	Diastolic Blood Pressure (DBP)	rs73102285	G	A	5.72	1.1E-08	-0.5	6.5E-01	rs1645761	T	C	-1.48	1.4E-01	4.0	7.3E-05	0.00	2102	0%	93%	0%	6%	0%
7	*PHACTR1*	Diastolic Blood Pressure (DBP)	rs6925904	G	A	-7.50	6.3E-14	-0.2	8.2E-01	rs17679286	G	A	2.74	6.2E-03	2.8	4.6E-03	0.04	1611	0%	99%	0%	1%	0%
8	*HTRA1*	Diastolic Blood Pressure (DBP)	rs2736923	A	G	5.47	4.6E-08	-3.5	4.8E-04	rs10490923	A	G	-3.00	2.7E-03	5.9	5.0E-09	0.02	1553	0%	0%	0%	99%	0%
9	*SESN3*	Diastolic Blood Pressure (DBP)	rs11021221	A	T	7.84	4.1E-15	-8.1	6.9E-16	rs11021221	A	T	7.84	4.1E-15	-8.1	6.9E-16	1.00	1360	0%	0%	0%	0%	100%
10	*LRP1*	Diastolic Blood Pressure (DBP)	rs11172113	C	T	-11.53	9.0E-31	-4.0	5.6E-05	rs2958124	A	C	2.00	4.5E-02	7.7	2.0E-14	0.01	1002	0%	0%	0%	100%	0%
11	*ATP2B1*	Diastolic Blood Pressure (DBP)	rs1689040	T	C	-6.17	7.0E-10	-16.5	2.8E-61	rs2681485	A	G	5.99	2.1E-09	16.7	1.3E-62	1.00	1023	0%	0%	0%	2%	98%
12	*COL4A1*	Diastolic Blood Pressure (DBP)	rs7326444	A	G	-6.46	1.0E-10	5.5	5.0E-08	rs9559749	A	G	0.73	4.6E-01	5.7	1.4E-08	0.00	1147	0%	0%	0%	5%	95%
12	*COL4A2*	Diastolic Blood Pressure (DBP)	rs11838776	A	G	-8.74	2.5E-18	5.1	2.6E-07	rs55940034	G	A	-8.56	1.2E-17	5.3	1.3E-07	0.94	1222	0%	0%	0%	0%	100%
13	*FBN1*	Diastolic Blood Pressure (DBP)	rs2437947	A	T	5.94	2.9E-09	3.7	2.5E-04	rs2245752	A	C	-2.61	9.0E-03	-5.4	8.3E-08	0.31	527	0%	2%	0%	95%	3%
14	*THSD4*	Diastolic Blood Pressure (DBP)	rs10851839	A	T	6.56	5.5E-11	8.7	2.4E-18	rs11853359	A	G	-5.54	3.0E-08	-9.1	1.3E-19	0.96	1140	0%	0%	0%	3%	97%
15	*MRPS6/SLC5A3/KCNE2*	Diastolic Blood Pressure (DBP)	rs9980618	T	C	-8.35	7.2E-17	4.2	2.5E-05	rs71326977	A	C	1.29	2.0E-01	-5.9	2.8E-09	0.01	1517	0%	1%	0%	92%	7%
16	*TIMP3*	Diastolic Blood Pressure (DBP)	rs137528	A	G	-5.40	6.8E-08	0.0	9.8E-01	rs16990642	A	T	0.29	7.7E-01	3.2	1.4E-03	0.00	1861	1%	98%	0%	1%	0%
1	*FGGY-DT*	Systolic Blood Pressure (PP)	rs34370185	T	G	7.09	1.4E-12	-8.0	1.3E-15	rs12758643	T	C	5.06	4.0E-07	-8.3	1.1E-16	0.96	1045	0%	0%	0%	8%	92%
2	*F3*	Systolic Blood Pressure (PP)	rs1146473	C	T	5.82	5.8E-09	0.9	3.8E-01	rs75088623	G	A	-0.60	5.5E-01	-5.8	5.1E-09	0.02	1577	0%	1%	0%	99%	0%
3	*ECM1/ADAMTSL4*	Systolic Blood Pressure (PP)	rs4970935	T	C	-13.04	6.1E-39	2.0	5.1E-02	rs11585169	A	T	-1.39	1.6E-01	5.8	5.3E-09	0.04	1035	0%	0%	0%	100%	0%
4	*AFAP1*	Systolic Blood Pressure (PP)	rs6828005	A	G	-6.67	2.6E-11	3.3	8.7E-04	rs4478172	C	A	5.02	5.3E-07	-4.8	1.5E-06	0.04	2202	0%	31%	0%	30%	39%
5	*ZNF827*	Systolic Blood Pressure (PP)	rs1507928	C	T	5.75	8.9E-09	-6.3	3.8E-10	rs13128814	A	G	5.53	3.2E-08	-7.1	1.2E-12	0.71	1244	0%	0%	0%	2%	98%

6	*ITGA1*	Systolic Blood Pressure (PP)	rs73102285	G	A	5.72	1.1E-08	3.3	9.2E-04	rs74344272	T	A	1.27	2.0E-01	4.3	1.9E-05	0.00	2098	0%	62%	0%	14%	24%
7	*PHACTR1*	Systolic Blood Pressure (PP)	rs6925904	G	A	-7.50	6.3E-14	-6.7	1.7E-11	rs2876301	T	C	-7.24	4.2E-13	-6.9	4.2E-12	0.99	1610	0%	0%	0%	3%	97%
8	*HTRA1*	Systolic Blood Pressure (PP)	rs2736923	A	G	5.47	4.6E-08	-1.8	6.9E-02	rs72834453	G	T	-2.73	6.4E-03	7.0	3.0E-12	0.02	1549	0%	0%	0%	99%	0%
9	*SESN3*	Systolic Blood Pressure (PP)	rs11021221	A	T	7.84	4.1E-15	0.0	9.6E-01	rs1047731	G	A	3.36	7.7E-04	2.6	8.2E-03	0.00	1357	0%	99%	0%	1%	0%
10	*LRP1*	Systolic Blood Pressure (PP)	rs11172113	C	T	-11.53	9.0E-31	1.6	1.2E-01	rs1296041	C	T	-0.35	7.2E-01	4.8	1.4E-06	0.00	1002	0%	31%	0%	69%	0%
11	*ATP2B1*	Systolic Blood Pressure (PP)	rs1689040	T	C	-6.17	7.0E-10	-18.5	3.7E-76	rs17249754	A	G	-5.61	2.0E-08	-21.0	1.3E-97	0.28	1021	0%	0%	0%	8%	92%
12	*COL4A1*	Systolic Blood Pressure (PP)	rs7326444	A	G	-6.46	1.0E-10	0.7	4.9E-01	rs4771653	T	C	0.48	6.3E-01	-4.9	9.9E-07	0.00	1145	0%	47%	0%	53%	0%
12	*COL4A2*	Systolic Blood Pressure (PP)	rs11838776	A	G	-8.74	2.5E-18	1.1	2.6E-01	rs9515270	T	C	-2.47	1.4E-02	5.3	1.0E-07	0.00	1220	0%	8%	0%	92%	0%
13	*FBN1*	Systolic Blood Pressure (PP)	rs2437947	A	T	5.94	2.9E-09	-7.9	2.6E-15	rs2437947	A	T	5.94	2.9E-09	-7.9	2.6E-15	1.00	527	0%	0%	0%	3%	97%
14	*THSD4*	Systolic Blood Pressure (PP)	rs10851839	A	T	6.56	5.5E-11	0.7	4.6E-01	rs985868	G	A	-0.61	5.4E-01	3.4	7.8E-04	0.00	1138	0%	99%	0%	1%	0%
15	*MRPS6/SLC5A3/KCNE2*	Systolic Blood Pressure (PP)	rs9980618	T	C	-8.35	7.2E-17	-2.4	1.4E-02	rs2834257	A	G	-1.34	1.8E-01	5.1	2.8E-07	0.00	1516	0%	21%	0%	78%	1%
16	*TIMP3*	Systolic Blood Pressure (PP)	rs137528	A	G	-5.40	6.8E-08	1.3	2.0E-01	rs2049948	G	A	1.20	2.3E-01	-3.2	1.2E-03	0.00	1857	1%	98%	0%	1%	0%
1	*FGGY-DT*	Fibromuscular Dysplasia	rs34370185	T	G	7.09	1.4E-12	1.4	1.5E-01	rs10493259	C	T	-0.65	5.2E-01	-2.9	3.6E-03	0.02	978	0%	86%	0%	8%	6%
2	*F3*	Fibromuscular Dysplasia	rs1146473	C	T	5.82	5.8E-09	0.8	4.0E-01	rs859068	T	C	-1.84	6.5E-02	-2.8	5.1E-03	0.00	1523	0%	86%	0%	10%	3%
3	*ECM1/ADAMTSL4*	Fibromuscular Dysplasia	rs4446975	T	C	-11.38	5.3E-30	-3.1	2.3E-03	rs11204662	G	T	-10.65	1.7E-26	-3.5	4.5E-04	0.89	808	0%	30%	0%	7%	63%
4	*AFAP1*	Fibromuscular Dysplasia	rs6828005	A	G	-6.67	2.6E-11	-0.8	4.2E-01	rs56411722	C	T	-5.04	4.5E-07	-3.5	5.0E-04	0.27	2031	0%	72%	0%	25%	3%
5	*ZNF827*	Fibromuscular Dysplasia	rs1507928	C	T	5.75	8.9E-09	3.3	8.1E-04	rs4544728	T	C	3.88	1.1E-04	4.1	3.6E-05	0.26	1079	0%	14%	0%	17%	69%
6	*ITGA1*	Fibromuscular Dysplasia	rs73102285	G	A	5.72	1.1E-08	1.0	3.3E-01	rs62357202	A	G	-2.35	1.9E-02	-2.8	4.8E-03	0.00	2040	0%	83%	0%	13%	4%
7	*PHACTR1*	Fibromuscular Dysplasia	rs6925904	G	A	-7.50	6.3E-14	-4.6	4.3E-06	rs7751826	T	C	-7.10	1.3E-12	-4.7	2.1E-06	0.97	1540	0%	0%	0%	2%	97%
8	*HTRA1*	Fibromuscular Dysplasia	rs2736923	A	G	5.47	4.6E-08	2.2	2.5E-02	rs3817285	C	T	-0.12	9.1E-01	-3.5	5.3E-04	0.00	1428	0%	71%	0%	10%	19%
9	*SESN3*	Fibromuscular Dysplasia	rs11021221	A	T	7.84	4.1E-15	-0.3	8.0E-01	rs75887387	T	G	-0.81	4.2E-01	-2.5	1.1E-02	0.00	1202	0%	91%	0%	6%	3%
10	*LRP1*	Fibromuscular Dysplasia	rs11172113	C	T	-11.53	9.0E-31	-6.4	2.0E-10	rs11172113	C	T	-11.53	9.0E-31	-6.4	2.0E-10	1.00	746	0%	0%	0%	0%	100%
11	*ATP2B1*	Fibromuscular Dysplasia	rs1689040	T	C	-6.17	7.0E-10	-3.6	3.1E-04	rs2681492	C	T	-5.51	3.6E-08	-5.6	1.7E-08	0.27	762	0%	0%	0%	10%	90%
12	*COL4A1*	Fibromuscular Dysplasia	rs7326444	A	G	-6.46	1.0E-10	-1.3	1.9E-01	rs80343422	G	A	0.09	9.3E-01	-2.4	1.5E-02	0.00	1040	0%	91%	0%	5%	5%
12	*COL4A2*	Fibromuscular Dysplasia	rs11838776	A	G	-8.74	2.5E-18	-3.7	1.8E-04	rs55940034	G	A	-8.56	1.2E-17	-3.8	1.4E-04	0.94	1130	0%	5%	0%	1%	94%
13	*FBN1*	Fibromuscular Dysplasia	rs2437947	A	T	5.94	2.9E-09	1.2	2.4E-01	rs9972344	G	T	-1.92	5.4E-02	-3.0	2.5E-03	0.00	393	0%	90%	0%	4%	6%
14	*THSD4*	Fibromuscular Dysplasia	rs10851839	A	T	6.56	5.5E-11	3.2	1.3E-03	rs11632680	G	T	2.91	3.6E-03	4.0	6.3E-05	0.21	1089	0%	26%	0%	7%	66%
15	*MRPS6/SLC5A3/KCNE2*	Fibromuscular Dysplasia	rs9980618	T	C	-8.35	7.2E-17	-3.0	2.4E-03	rs3746861	T	C	2.17	3.0E-02	3.3	1.1E-03	0.01	1481	0%	34%	0%	6%	60%
16	*TIMP3*	Fibromuscular Dysplasia	rs137528	A	G	-5.40	6.8E-08	-1.1	2.7E-01	rs142100390	A	C	-3.28	1.1E-03	-3.2	1.2E-03	0.19	1734	1%	81%	0%	15%	4%
1	*FGGY-DT*	Coronary Artery Disease (CAD)	rs34370185	T	G	7.09	1.4E-12	-2.7	6.1E-03	rs932773	C	G	2.06	4.0E-02	4.1	4.1E-05	0.01	947	0%	76%	0%	12%	12%
2	*F3*	Coronary Artery Disease (CAD)	rs1146473	C	T	5.82	5.8E-09	-0.1	9.4E-01	rs17398377	C	T	1.43	1.5E-01	2.7	7.1E-03	0.01	1440	0%	98%	0%	1%	0%
3	*ECM1/ADAMTSL4*	Coronary Artery Disease (CAD)	rs4970935	T	C	-13.04	6.1E-39	4.2	2.2E-05	rs7549723	T	C	-3.31	9.5E-04	5.2	1.8E-07	0.10	868	0%	1%	0%	60%	39%
4	*AFAP1*	Coronary Artery Disease (CAD)	rs6828005	A	G	-6.67	2.6E-11	1.8	7.4E-02	rs9968354	C	T	1.01	3.1E-01	-3.7	2.1E-04	0.08	1658	0%	92%	0%	6%	1%
5	*ZNF827*	Coronary Artery Disease (CAD)	rs1507928	C	T	5.75	8.9E-09	-3.7	2.4E-04	rs4345206	C	T	5.01	5.6E-07	-4.1	4.1E-05	0.63	871	0%	43%	0%	3%	53%
6	*ITGA1*	Coronary Artery Disease (CAD)	rs73102285	G	A	5.72	1.1E-08	0.3	7.8E-01	rs62357230	A	G	-1.37	1.7E-01	3.9	1.2E-04	0.00	1719	0%	85%	0%	15%	0%
7	*PHACTR1*	Coronary Artery Disease (CAD)	rs6925904	G	A	-7.50	6.3E-14	11.5	2.4E-30	rs6925904	G	A	-7.50	6.3E-14	11.5	2.4E-30	1.00	1227	0%	0%	0%	0%	100%
8	*HTRA1*	Coronary Artery Disease (CAD)	rs72631113	T	C	5.42	5.9E-08	-4.9	1.1E-06	rs77494534	T	C	-0.05	9.6E-01	5.6	1.7E-08	0.02	1332	0%	0%	0%	16%	84%
9	*SESN3*	Coronary Artery Disease (CAD)	rs11021221	A	T	7.84	4.1E-15	-2.2	3.0E-02	rs7938589	A	G	-0.76	4.5E-01	-3.6	3.0E-04	0.00	1107	0%	92%	0%	4%	4%
10	*LRP1*	Coronary Artery Disease (CAD)	rs11172113	C	T	-11.53	9.0E-31	3.2	1.2E-03	rs507562	G	C	1.76	7.9E-02	4.7	3.0E-06	0.00	775	0%	27%	0%	57%	16%
11	*ATP2B1*	Coronary Artery Disease (CAD)	rs1689040	T	C	-6.17	7.0E-10	3.3	8.9E-04	rs12579302	G	A	-5.62	2.0E-08	6.4	1.2E-10	0.28	962	0%	0%	0%	9%	91%
12	*COL4A1*	Coronary Artery Disease (CAD)	rs7326444	A	G	-6.46	1.0E-10	6.5	9.4E-11	rs1000989	C	T	-5.08	3.8E-07	7.0	2.3E-12	0.89	1076	0%	0%	0%	15%	85%
12	*COL4A2*	Coronary Artery Disease (CAD)	rs11838776	A	G	-8.74	2.5E-18	6.6	3.6E-11	rs11838776	A	G	-8.74	2.5E-18	6.6	3.6E-11	1.00	1053	0%	0%	0%	0%	100%
13	*FBN1*	Coronary Artery Disease (CAD)	rs2437947	A	T	5.94	2.9E-09	-1.9	5.2E-02	rs11638254	A	T	-0.11	9.2E-01	2.5	1.4E-02	0.01	367	0%	96%	0%	0%	4%
14	*THSD4*	Coronary Artery Disease (CAD)	rs10851839	A	T	6.56	5.5E-11	-1.6	1.2E-01	rs4300598	T	C	0.01	9.9E-01	2.5	1.1E-02	0.00	1050	0%	98%	0%	1%	1%
15	*MRPS6/SLC5A3/KCNE2*	Coronary Artery Disease (CAD)	rs9980618	T	C	-8.35	7.2E-17	9.7	3.0E-22	rs9980618	T	C	-8.35	7.2E-17	9.7	3.0E-22	1.00	1351	0%	0%	0%	0%	100%
16	*TIMP3*	Coronary Artery Disease (CAD)	rs137528	A	G	-5.40	6.8E-08	2.7	7.7E-03	rs5754240	A	G	2.01	4.4E-02	-4.3	2.1E-05	0.05	1652	0%	46%	0%	9%	44%
1	*FGGY-DT*	Any stroke (AS)	rs34370185	T	G	7.09	1.4E-12	0.0	9.8E-01	rs12738848	G	T	-0.21	8.4E-01	3.0	2.9E-03	0.00	1040	0%	98%	0%	2%	1%
2	*F3*	Any stroke (AS)	rs1146473	C	T	5.82	5.8E-09	0.3	8.0E-01	rs77568287	A	G	0.16	8.7E-01	-3.3	8.6E-04	0.00	1560	0%	96%	0%	4%	1%
3	*ECM1/ADAMTSL4*	Any stroke (AS)	rs1260387	C	T	-12.21	3.2E-34	1.2	2.5E-01	rs12403795	T	C	8.04	8.9E-16	-2.3	2.0E-02	0.41	1028	0%	98%	0%	1%	1%
4	*AFAP1*	Any stroke (AS)	rs6828005	A	G	-6.67	2.6E-11	0.5	6.5E-01	rs67667723	C	A	-0.80	4.3E-01	-3.5	5.8E-04	0.00	2204	0%	94%	0%	5%	1%
5	*ZNF827*	Any stroke (AS)	rs1507928	C	T	5.75	8.9E-09	0.2	8.2E-01	rs11733094	A	G	0.44	6.6E-01	3.0	2.6E-03	0.01	1232	0%	96%	0%	3%	0%
6	*ITGA1*	Any stroke (AS)	rs73102285	G	A	5.72	1.1E-08	-3.2	1.5E-03	rs16880353	G	A	5.63	1.8E-08	-3.4	6.0E-04	0.96	2088	0%	52%	0%	3%	44%
7	*PHACTR1*	Any stroke (AS)	rs6925904	G	A	-7.50	6.3E-14	-0.9	3.9E-01	rs6900427	A	G	2.80	5.1E-03	2.1	3.2E-02	0.02	1608	0%	97%	0%	2%	1%
8	*HTRA1*	Any stroke (AS)	rs2736923	A	G	5.47	4.6E-08	0.0	9.7E-01	rs2284665	T	G	4.95	7.6E-07	-5.4	6.0E-08	0.02	1549	0%	0%	0%	18%	82%
9	*SESN3*	Any stroke (AS)	rs11021221	A	T	7.84	4.1E-15	-1.8	7.4E-02	rs7110456	C	T	0.05	9.6E-01	-2.8	5.3E-03	0.00	1353	0%	95%	0%	2%	3%
10	*LRP1*	Any stroke (AS)	rs11172113	C	T	-11.53	9.0E-31	1.9	6.0E-02	rs2229717	T	G	-1.73	8.3E-02	4.0	7.4E-05	0.00	993	0%	92%	0%	5%	3%
11	*ATP2B1*	Any stroke (AS)	rs1689040	T	C	-6.17	7.0E-10	-1.9	5.9E-02	rs7294375	G	T	3.92	9.1E-05	5.1	3.3E-07	0.10	1019	0%	3%	0%	93%	4%
12	*COL4A1*	Any stroke (AS)	rs7326444	A	G	-6.46	1.0E-10	3.9	8.6E-05	rs9521635	C	T	-6.05	1.4E-09	4.5	9.4E-06	0.92	1142	0%	5%	0%	5%	90%
12	*COL4A2*	Any stroke (AS)	rs11838776	A	G	-8.74	2.5E-18	2.4	1.6E-02	rs12865767	T	A	0.32	7.5E-01	2.9	3.4E-03	0.00	1210	0%	90%	0%	2%	8%
13	*FBN1*	Any stroke (AS)	rs2437947	A	T	5.94	2.9E-09	-0.4	7.0E-01	rs7169672	T	C	-0.53	6.0E-01	2.0	4.9E-02	0.01	522	0%	99%	0%	0%	1%
14	*THSD4*	Any stroke (AS)	rs10851839	A	T	6.56	5.5E-11	0.8	4.3E-01	rs1866920	C	T	2.84	4.5E-03	3.0	2.8E-03	0.12	1131	0%	98%	0%	2%	1%
15	*MRPS6/SLC5A3/KCNE2*	Any stroke (AS)	rs9980618	T	C	-8.35	7.2E-17	-1.6	1.1E-01	rs34545319	G	A	0.19	8.5E-01	2.9	4.3E-03	0.01	1516	0%	95%	0%	3%	2%
16	*TIMP3*	Any stroke (AS)	rs137528	A	G	-5.40	6.8E-08	0.1	9.3E-01	rs7289631	A	G	-1.55	1.2E-01	-3.8	1.6E-04	0.18	1852	1%	87%	0%	12%	1%
1	*FGGY-DT*	Cardioembolic Stroke (CES)	rs34370185	T	G	7.09	1.4E-12	0.0	9.8E-01	rs6664131	A	C	1.31	1.9E-01	-3.1	1.9E-03	0.00	1040	0%	94%	0%	5%	1%
2	*F3*	Cardioembolic Stroke (CES)	rs1146473	C	T	5.82	5.8E-09	0.3	7.7E-01	rs79658099	C	T	-0.60	5.5E-01	3.0	3.0E-03	0.00	1560	0%	93%	0%	6%	1%
3	*ECM1/ADAMTSL4*	Cardioembolic Stroke (CES)	rs1260387	C	T	-12.21	3.2E-34	1.8	6.7E-02	rs12134682	C	G	-2.14	3.3E-02	3.3	8.0E-04	0.05	1028	0%	82%	0%	13%	5%
4	*AFAP1*	Cardioembolic Stroke (CES)	rs6828005	A	G	-6.67	2.6E-11	0.2	8.5E-01	rs6848741	G	A	-0.89	3.7E-01	-3.2	1.5E-03	0.01	2204	0%	91%	0%	7%	1%
5	*ZNF827*	Cardioembolic Stroke (CES)	rs1507928	C	T	5.75	8.9E-09	1.1	2.5E-01	rs72729816	A	C	-0.34	7.4E-01	3.0	2.4E-03	0.00	1232	0%	94%	0%	4%	2%

6	*ITGA1*	Cardioembolic Stroke (CES)	rs73102285	G	A	5.72	1.1E-08	-1.8	7.4E-02	rs2126952	C	T	-2.75	6.0E-03	3.8	1.4E-04	0.44	2088	0%	83%	0%	11%	5%
7	*PHACTR1*	Cardioembolic Stroke (CES)	rs6925904	G	A	-7.50	6.3E-14	-0.4	7.1E-01	rs116314410	A	T	1.69	9.1E-02	2.9	3.3E-03	0.00	1608	0%	95%	0%	4%	1%
8	*HTRA1*	Cardioembolic Stroke (CES)	rs2736923	A	G	5.47	4.6E-08	-1.2	2.3E-01	rs1891114	G	A	0.35	7.3E-01	3.0	2.9E-03	0.00	1549	0%	91%	0%	5%	4%
9	*SESN3*	Cardioembolic Stroke (CES)	rs11021221	A	T	7.84	4.1E-15	0.6	5.5E-01	rs10831314	T	C	2.67	7.5E-03	3.5	4.1E-04	0.00	1353	0%	91%	0%	7%	1%
10	*LRP1*	Cardioembolic Stroke (CES)	rs11172113	C	T	-11.53	9.0E-31	-0.3	7.7E-01	rs2229717	T	G	-1.73	8.3E-02	3.3	1.1E-03	0.00	993	0%	97%	0%	2%	1%
11	*ATP2B1*	Cardioembolic Stroke (CES)	rs1689040	T	C	-6.17	7.0E-10	-1.3	1.8E-01	rs7960337	C	T	4.87	1.1E-06	2.4	1.4E-02	0.09	1019	0%	95%	0%	3%	2%
12	*COL4A1*	Cardioembolic Stroke (CES)	rs7326444	A	G	-6.46	1.0E-10	0.6	5.6E-01	rs681777	C	G	0.63	5.3E-01	-2.9	3.3E-03	0.00	1142	0%	94%	0%	4%	1%
12	*COL4A2*	Cardioembolic Stroke (CES)	rs11838776	A	G	-8.74	2.5E-18	0.7	4.9E-01	rs9588247	T	G	-1.81	7.0E-02	2.9	3.6E-03	0.00	1210	0%	95%	0%	4%	1%
13	*FBN1*	Cardioembolic Stroke (CES)	rs2437947	A	T	5.94	2.9E-09	1.6	1.0E-01	rs74773702	C	T	-0.43	6.7E-01	2.0	4.4E-02	0.01	522	0%	94%	0%	1%	5%
14	*THSD4*	Cardioembolic Stroke (CES)	rs10851839	A	T	6.56	5.5E-11	-0.9	3.5E-01	rs28693764	A	C	0.19	8.4E-01	-2.5	1.1E-02	0.00	1131	0%	96%	0%	2%	2%
15	*MRPS6/SLC5A3/KCNE2*	Cardioembolic Stroke (CES)	rs9980618	T	C	-8.35	7.2E-17	1.0	3.0E-01	rs41314677	G	A	-0.86	3.9E-01	2.9	3.6E-03	0.00	1516	0%	93%	0%	5%	2%
16	*TIMP3*	Cardioembolic Stroke (CES)	rs137528	A	G	-5.40	6.8E-08	1.0	3.0E-01	rs9621514	T	C	0.75	4.5E-01	3.6	3.8E-04	0.00	1852	1%	85%	0%	13%	2%
1	*FGGY-DT*	Small Vessel Stroke (SVS)	rs34370185	T	G	7.09	1.4E-12	-0.1	9.3E-01	rs114186484	T	C	-1.01	3.1E-01	2.6	9.6E-03	0.01	1040	0%	96%	0%	3%	1%
2	*F3*	Small Vessel Stroke (SVS)	rs1146473	C	T	5.82	5.8E-09	0.2	8.4E-01	rs6675132	T	G	-1.21	2.3E-01	-2.7	7.1E-03	0.00	1560	0%	93%	0%	6%	1%
3	*ECM1/ADAMTSL4*	Small Vessel Stroke (SVS)	rs1260387	C	T	-12.21	3.2E-34	-0.4	7.0E-01	rs4970966	T	G	2.56	1.1E-02	2.4	1.8E-02	0.01	1028	0%	97%	0%	2%	1%
4	*AFAP1*	Small Vessel Stroke (SVS)	rs6828005	A	G	-6.67	2.6E-11	-0.1	9.5E-01	rs4643793	T	C	0.01	9.9E-01	3.8	1.4E-04	0.00	2204	0%	85%	0%	14%	1%
5	*ZNF827*	Small Vessel Stroke (SVS)	rs1507928	C	T	5.75	8.9E-09	-0.5	6.4E-01	rs7694604	G	T	-0.57	5.7E-01	4.5	8.6E-06	0.00	1232	0%	21%	0%	79%	0%
6	*ITGA1*	Small Vessel Stroke (SVS)	rs73102285	G	A	5.72	1.1E-08	-2.5	1.3E-02	rs72752528	T	C	0.54	5.9E-01	2.8	5.1E-03	0.00	2088	0%	73%	0%	8%	19%
7	*PHACTR1*	Small Vessel Stroke (SVS)	rs6925904	G	A	-7.50	6.3E-14	-1.8	7.7E-02	rs9471949	C	T	-0.03	9.7E-01	3.0	3.0E-03	0.00	1608	0%	91%	0%	5%	4%
8	*HTRA1*	Small Vessel Stroke (SVS)	rs2736923	A	G	5.47	4.6E-08	-1.6	1.2E-01	rs2142308	C	G	4.35	1.3E-05	-4.5	5.6E-06	0.02	1549	0%	3%	0%	10%	87%
9	*SESN3*	Small Vessel Stroke (SVS)	rs11021221	A	T	7.84	4.1E-15	-2.5	1.3E-02	rs4384353	C	T	-4.92	8.9E-07	3.2	1.2E-03	0.40	1353	0%	70%	0%	6%	24%
10	*LRP1*	Small Vessel Stroke (SVS)	rs11172113	C	T	-11.53	9.0E-31	0.3	7.3E-01	rs142888721	A	G	1.00	3.2E-01	3.7	2.2E-04	0.03	993	0%	88%	0%	10%	1%
11	*ATP2B1*	Small Vessel Stroke (SVS)	rs1689040	T	C	-6.17	7.0E-10	0.0	9.6E-01	rs1895711	T	A	3.98	7.2E-05	3.7	2.1E-04	0.10	1019	0%	80%	0%	19%	1%
12	*COL4A1*	Small Vessel Stroke (SVS)	rs7326444	A	G	-6.46	1.0E-10	3.5	4.6E-04	rs9521635	C	T	-6.05	1.4E-09	4.1	3.6E-05	0.92	1142	0%	12%	0%	6%	82%
12	*COL4A2*	Small Vessel Stroke (SVS)	rs11838776	A	G	-8.74	2.5E-18	4.6	4.9E-06	rs55940034	G	A	-8.56	1.2E-17	4.6	4.9E-06	0.94	1210	0%	0%	0%	0%	100%
13	*FBN1*	Small Vessel Stroke (SVS)	rs2437947	A	T	5.94	2.9E-09	-0.2	8.6E-01	rs2009404	T	G	0.49	6.2E-01	-2.6	8.5E-03	0.00	522	0%	96%	0%	2%	2%
14	*THSD4*	Small Vessel Stroke (SVS)	rs10851839	A	T	6.56	5.5E-11	0.0	1.0E+00	rs77103696	A	G	1.05	2.9E-01	2.3	2.2E-02	0.00	1131	0%	96%	0%	3%	1%
15	*MRPS6/SLC5A3/KCNE2*	Small Vessel Stroke (SVS)	rs9980618	T	C	-8.35	7.2E-17	-1.3	2.0E-01	rs7282240	T	C	-3.80	1.5E-04	-3.4	8.0E-04	0.28	1516	0%	90%	0%	6%	4%
16	*TIMP3*	Small Vessel Stroke (SVS)	rs137528	A	G	-5.40	6.8E-08	-0.5	6.0E-01	rs5994625	G	A	0.39	7.0E-01	-3.9	9.6E-05	0.02	1852	1%	78%	0%	19%	3%
1	*FGGY-DT*	Forced expiratory volume in 1 sec (FEV1)	rs34370185	T	G	7.09	1.4E-12	0.0	9.8E-01	rs114186484	T	C	-1.01	3.1E-01	-2.7	6.4E-03	0.01	1045	0%	99%	0%	0%	0%
2	*F3*	Forced expiratory volume in 1 sec (FEV1)	rs1146473	C	T	5.82	5.8E-09	-0.5	6.0E-01	rs112292390	C	G	0.62	5.3E-01	3.2	1.6E-03	0.00	1576	0%	98%	0%	2%	0%
3	*ECM1/ADAMTSL4*	Forced expiratory volume in 1 sec (FEV1)	rs4970935	T	C	-13.04	6.1E-39	-3.8	1.5E-04	rs10888385	C	T	-0.98	3.2E-01	-7.6	3.5E-14	0.04	1033	0%	0%	0%	100%	0%
4	*AFAP1*	Forced expiratory volume in 1 sec (FEV1)	rs6828005	A	G	-6.67	2.6E-11	0.3	7.8E-01	rs4478172	C	A	5.02	5.3E-07	-5.8	6.0E-09	0.04	2214	0%	0%	0%	97%	3%
5	*ZNF827*	Forced expiratory volume in 1 sec (FEV1)	rs1507928	C	T	5.75	8.9E-09	2.7	6.2E-03	rs2135963	G	C	1.62	1.0E-01	4.7	2.9E-06	0.03	1241	0%	68%	0%	28%	4%
6	*ITGA1*	Forced expiratory volume in 1 sec (FEV1)	rs73102285	G	A	5.72	1.1E-08	-1.8	7.0E-02	rs9791046	A	T	-0.42	6.8E-01	5.6	2.1E-08	0.00	2096	0%	1%	0%	99%	0%
7	*PHACTR1*	Forced expiratory volume in 1 sec (FEV1)	rs6925904	G	A	-7.50	6.3E-14	0.3	7.6E-01	rs1931546	C	T	1.29	2.0E-01	-2.6	1.0E-02	0.00	1613	0%	99%	0%	1%	0%
8	*HTRA1*	Forced expiratory volume in 1 sec (FEV1)	rs2736923	A	G	5.47	4.6E-08	-0.9	3.9E-01	rs12571363	A	C	-3.60	3.2E-04	-7.7	1.6E-14	0.00	1553	0%	0%	0%	97%	3%
9	*SESN3*	Forced expiratory volume in 1 sec (FEV1)	rs11021221	A	T	7.84	4.1E-15	-2.9	3.9E-03	rs8181535	T	C	4.26	2.0E-05	-3.5	5.6E-04	0.47	1359	0%	86%	0%	2%	12%
10	*LRP1*	Forced expiratory volume in 1 sec (FEV1)	rs11172113	C	T	-11.53	9.0E-31	0.2	8.1E-01	rs146529565	C	A	0.35	7.3E-01	4.2	2.3E-05	0.03	991	0%	60%	0%	40%	0%
11	*ATP2B1*	Forced expiratory volume in 1 sec (FEV1)	rs1689040	T	C	-6.17	7.0E-10	1.3	1.9E-01	rs17456901	C	T	-1.48	1.4E-01	4.4	1.3E-05	0.00	1017	0%	76%	0%	24%	0%
12	*COL4A1*	Forced expiratory volume in 1 sec (FEV1)	rs7326444	A	G	-6.46	1.0E-10	-0.5	6.3E-01	rs9301442	C	T	1.86	6.2E-02	-3.3	8.4E-04	0.00	1148	0%	99%	0%	1%	0%
12	*COL4A2*	Forced expiratory volume in 1 sec (FEV1)	rs11838776	A	G	-8.74	2.5E-18	0.9	3.7E-01	rs9515279	T	A	1.62	1.1E-01	-3.9	8.3E-05	0.00	1223	0%	93%	0%	7%	0%
13	*FBN1*	Forced expiratory volume in 1 sec (FEV1)	rs2437947	A	T	5.94	2.9E-09	-0.9	3.8E-01	rs8040242	C	A	-0.20	8.5E-01	-2.9	3.6E-03	0.01	527	0%	99%	0%	1%	0%
14	*THSD4*	Forced expiratory volume in 1 sec (FEV1)	rs10851839	A	T	6.56	5.5E-11	6.1	1.4E-09	rs8033889	T	G	-5.31	1.1E-07	-6.8	1.4E-11	0.30	1135	0%	0%	0%	34%	66%
15	*MRPS6/SLC5A3/KCNE2*	Forced expiratory volume in 1 sec (FEV1)	rs9980618	T	C	-8.35	7.2E-17	-2.5	1.4E-02	rs2236610	G	C	0.99	3.2E-01	-4.4	1.3E-05	0.00	1518	0%	66%	0%	31%	3%
16	*TIMP3*	Forced expiratory volume in 1 sec (FEV1)	rs137528	A	G	-5.40	6.8E-08	2.6	1.0E-02	rs5998705	G	A	2.81	5.0E-03	-4.4	1.3E-05	0.20	1863	1%	84%	0%	10%	5%
1	*FGGY-DT*	HDL	rs34370185	T	G	7.09	1.4E-12	-0.4	6.7E-01	rs2492323	T	G	0.05	9.6E-01	3.0	2.3E-03	0.00	1045	0%	99%	0%	1%	0%
2	*F3*	HDL	rs1146473	C	T	5.82	5.8E-09	-0.1	9.1E-01	rs4147820	T	C	-1.11	2.7E-01	-4.4	1.1E-05	0.00	1576	0%	94%	0%	6%	0%
3	*ECM1/ADAMTSL4*	HDL	rs4970935	T	C	-13.04	6.1E-39	2.9	3.8E-03	rs41271951	G	A	-1.31	1.9E-01	7.7	1.2E-14	0.01	1033	0%	0%	0%	100%	0%
4	*AFAP1*	HDL	rs6828005	A	G	-6.67	2.6E-11	1.1	2.6E-01	rs2002574	C	T	0.20	8.5E-01	-4.0	5.7E-05	0.00	2214	0%	97%	0%	2%	0%
5	*ZNF827*	HDL	rs1507928	C	T	5.75	8.9E-09	1.3	1.8E-01	rs13134992	T	C	0.40	6.9E-01	5.6	1.8E-08	0.01	1241	0%	2%	0%	98%	0%
6	*ITGA1*	HDL	rs73102285	G	A	5.72	1.1E-08	0.8	4.2E-01	rs61448800	A	G	0.98	3.3E-01	-3.6	2.8E-04	0.00	2096	0%	95%	0%	4%	0%
7	*PHACTR1*	HDL	rs6925904	G	A	-7.50	6.3E-14	1.4	1.5E-01	rs4714946	A	G	3.74	1.8E-04	-2.9	3.6E-03	0.41	1613	0%	98%	0%	1%	0%
8	*HTRA1*	HDL	rs2736923	A	G	5.47	4.6E-08	-1.7	9.7E-02	rs9423289	T	C	0.09	9.3E-01	4.0	7.6E-05	0.00	1553	0%	96%	0%	3%	1%
9	*SESN3*	HDL	rs11021221	A	T	7.84	4.1E-15	0.6	5.4E-01	rs11021232	C	T	-1.53	1.3E-01	-6.0	1.7E-09	0.05	1359	0%	0%	0%	100%	0%
10	*LRP1*	HDL	rs11172113	C	T	-11.53	9.0E-31	3.8	1.3E-04	rs61352607	T	G	-1.66	9.7E-02	12.3	6.9E-35	0.02	991	0%	0%	0%	100%	0%
11	*ATP2B1*	HDL	rs1689040	T	C	-6.17	7.0E-10	0.5	6.3E-01	rs12371151	T	A	2.02	4.3E-02	-3.3	8.5E-04	0.00	1017	0%	99%	0%	1%	0%
12	*COL4A1*	HDL	rs7326444	A	G	-6.46	1.0E-10	-0.5	6.3E-01	rs11841319	T	C	-1.30	1.9E-01	-3.5	5.0E-04	0.00	1148	0%	99%	0%	1%	0%
12	*COL4A2*	HDL	rs11838776	A	G	-8.74	2.5E-18	0.9	3.5E-01	rs9521977	C	T	-0.66	5.1E-01	-4.3	1.6E-05	0.00	1223	0%	91%	0%	9%	0%
13	*FBN1*	HDL	rs2437947	A	T	5.94	2.9E-09	-2.3	2.4E-02	rs117540572	A	G	0.92	3.6E-01	3.0	2.9E-03	0.00	527	0%	96%	0%	1%	3%
14	*THSD4*	HDL	rs10851839	A	T	6.56	5.5E-11	2.2	3.0E-02	rs75125772	C	G	-0.64	5.2E-01	2.8	5.0E-03	0.00	1135	0%	98%	0%	1%	2%
15	*MRPS6/SLC5A3/KCNE2*	HDL	rs9980618	T	C	-8.35	7.2E-17	1.6	1.0E-01	rs9978142	T	A	-5.53	3.3E-08	2.8	5.7E-03	0.67	1518	0%	98%	0%	1%	1%
16	*TIMP3*	HDL	rs137528	A	G	-5.40	6.8E-08	-0.4	6.6E-01	rs58930195	C	T	0.31	7.6E-01	2.2	2.5E-02	0.00	1863	1%	98%	0%	1%	0%
1	*FGGY-DT*	LDL	rs34370185	T	G	7.09	1.4E-12	2.6	8.7E-03	rs6678642	G	T	6.12	9.7E-10	3.0	2.4E-03	0.70	1045	0%	93%	0%	2%	6%
2	*F3*	LDL	rs1146473	C	T	5.82	5.8E-09	2.0	4.9E-02	rs11807145	T	C	0.73	4.6E-01	-3.6	3.4E-04	0.04	1576	0%	97%	0%	2%	1%
3	*ECM1/ADAMTSL4*	LDL	rs4970935	T	C	-13.04	6.1E-39	-2.2	2.9E-02	rs267733	G	A	-2.99	2.8E-03	-5.2	2.2E-07	0.02	1033	0%	20%	0%	79%	0%
4	*AFAP1*	LDL	rs6828005	A	G	-6.67	2.6E-11	-0.8	4.4E-01	rs2002574	C	T	0.20	8.5E-01	-4.6	3.8E-06	0.00	2214	0%	91%	0%	8%	1%
5	*ZNF827*	LDL	rs1507928	C	T	5.75	8.9E-09	0.1	8.9E-01	rs6819403	A	C	-0.44	6.6E-01	3.0	2.6E-03	0.00	1241	0%	99%	0%	1%	0%

6	*ITGA1*	LDL	rs73102285	G	A	5.72	1.1E-08	-0.6	5.4E-01	rs116734477	T	C	1.50	1.3E-01	-8.7	4.5E-18	0.00	2096	0%	0%	0%	100%	0%
7	*PHACTR1*	LDL	rs6925904	G	A	-7.50	6.3E-14	-1.4	1.7E-01	rs36102538	G	A	0.61	5.4E-01	2.6	8.3E-03	0.01	1613	0%	99%	0%	1%	0%
8	*HTRA1*	LDL	rs2736923	A	G	5.47	4.6E-08	-2.0	4.6E-02	rs9423289	T	C	0.09	9.3E-01	6.9	6.0E-12	0.00	1553	0%	0%	0%	100%	0%
9	*SESN3*	LDL	rs11021221	A	T	7.84	4.1E-15	2.9	4.0E-03	rs3858379	C	T	-0.44	6.6E-01	-4.3	1.6E-05	0.00	1359	0%	82%	0%	7%	11%
10	*LRP1*	LDL	rs11172113	C	T	-11.53	9.0E-31	0.8	4.0E-01	rs2122982	A	G	-1.85	6.5E-02	-6.0	1.5E-09	0.02	991	0%	0%	0%	100%	0%
11	*ATP2B1*	LDL	rs1689040	T	C	-6.17	7.0E-10	2.3	2.3E-02	rs12306780	T	A	1.05	2.9E-01	4.5	5.6E-06	0.00	1017	0%	44%	0%	55%	1%
12	*COL4A1*	LDL	rs7326444	A	G	-6.46	1.0E-10	-1.0	3.4E-01	rs532625	T	A	0.43	6.7E-01	-3.9	8.0E-05	0.01	1148	0%	98%	0%	2%	0%
12	*COL4A2*	LDL	rs11838776	A	G	-8.74	2.5E-18	-5.3	1.2E-07	rs55940034	G	A	-8.56	1.2E-17	-5.4	6.9E-08	0.94	1223	0%	0%	0%	1%	99%
13	*FBN1*	LDL	rs2437947	A	T	5.94	2.9E-09	1.7	9.3E-02	rs363820	C	T	5.16	2.5E-07	1.9	6.0E-02	0.90	527	0%	99%	0%	0%	1%
14	*THSD4*	LDL	rs10851839	A	T	6.56	5.5E-11	-1.5	1.3E-01	rs13379731	C	G	-1.47	1.4E-01	3.4	5.9E-04	0.09	1135	0%	98%	0%	1%	0%
15	*MRPS6/SLC5A3/KCNE2*	LDL	rs9980618	T	C	-8.35	7.2E-17	-1.3	1.8E-01	rs34323310	T	C	0.73	4.7E-01	-4.0	6.5E-05	0.00	1518	0%	93%	0%	6%	0%
16	*TIMP3*	LDL	rs137528	A	G	-5.40	6.8E-08	-0.8	4.0E-01	rs11703454	A	G	-2.36	1.8E-02	-2.7	7.2E-03	0.00	1863	1%	98%	0%	1%	0%
1	*FGGY-DT*	Migraine	rs34370185	T	G	7.09	1.4E-12	0.2	8.2E-01	rs932770	A	G	1.81	7.1E-02	3.2	1.6E-03	0.01	1045	0%	100%	0%	0%	0%
2	*F3*	Migraine	rs1146473	C	T	5.82	5.8E-09	-0.3	7.9E-01	rs560426	T	C	0.76	4.5E-01	-2.8	4.9E-03	0.01	1576	0%	100%	0%	0%	0%
3	*ECM1/ADAMTSL4*	Migraine	rs4970935	T	C	-13.04	6.1E-39	-4.6	5.0E-06	rs6693567	T	C	-11.13	9.8E-29	-5.2	1.9E-07	0.90	1033	0%	11%	0%	2%	86%
4	*AFAP1*	Migraine	rs6828005	A	G	-6.67	2.6E-11	-2.1	3.3E-02	rs4696794	G	A	-2.92	3.5E-03	-3.7	2.1E-04	0.17	2214	0%	99%	0%	0%	0%
5	*ZNF827*	Migraine	rs1507928	C	T	5.75	8.9E-09	-0.1	9.0E-01	rs10029927	T	C	0.33	7.4E-01	2.8	5.3E-03	0.01	1241	0%	100%	0%	0%	0%
6	*ITGA1*	Migraine	rs73102285	G	A	5.72	1.1E-08	0.0	9.9E-01	rs78464352	A	G	-1.55	1.2E-01	-3.2	1.3E-03	0.00	2096	0%	100%	0%	0%	0%
7	*PHACTR1*	Migraine	rs6925904	G	A	-7.50	6.3E-14	-6.4	1.2E-10	rs1332844	T	C	-7.38	1.7E-13	-6.5	8.1E-11	1.00	1613	0%	0%	0%	2%	98%
8	*HTRA1*	Migraine	rs2736923	A	G	5.47	4.6E-08	2.9	3.2E-03	rs139101261	C	G	-3.15	1.6E-03	-3.5	4.0E-04	0.01	1553	0%	97%	0%	1%	2%
9	*SESN3*	Migraine	rs11021221	A	T	7.84	4.1E-15	2.1	3.3E-02	rs148907559	T	A	0.03	9.8E-01	3.0	2.5E-03	0.01	1359	0%	100%	0%	0%	0%
10	*LRP1*	Migraine	rs11172113	C	T	-11.53	9.0E-31	-10.1	4.0E-24	rs11172113	C	T	-11.53	9.0E-31	-10.1	4.0E-24	1.00	991	0%	0%	0%	0%	100%
11	*ATP2B1*	Migraine	rs1689040	T	C	-6.17	7.0E-10	-2.8	5.7E-03	rs6538222	A	G	0.90	3.7E-01	3.0	3.1E-03	0.00	1017	0%	99%	0%	0%	1%
12	*COL4A1*	Migraine	rs7326444	A	G	-6.46	1.0E-10	-1.9	5.3E-02	rs7995770	G	A	-0.33	7.4E-01	-4.0	6.4E-05	0.00	1148	0%	99%	0%	0%	0%
12	*COL4A2*	Migraine	rs11838776	A	G	-8.74	2.5E-18	-2.5	1.4E-02	rs9521762	A	G	0.96	3.4E-01	-3.7	2.6E-04	0.05	1223	0%	99%	0%	0%	0%
13	*FBN1*	Migraine	rs2437947	A	T	5.94	2.9E-09	-0.2	8.1E-01	rs28730795	G	T	1.69	9.1E-02	2.2	2.7E-02	0.24	527	0%	100%	0%	0%	0%
14	*THSD4*	Migraine	rs10851839	A	T	6.56	5.5E-11	0.2	8.1E-01	rs138365904	G	A	-1.95	5.1E-02	3.3	9.5E-04	0.11	1135	0%	100%	0%	0%	0%
15	*MRPS6/SLC5A3/KCNE2*	Migraine	rs9980618	T	C	-8.35	7.2E-17	-3.9	8.5E-05	rs8131284	C	T	-7.61	2.8E-14	-4.0	6.1E-05	0.80	1518	0%	57%	0%	1%	42%
16	*TIMP3*	Migraine	rs137528	A	G	-5.40	6.8E-08	-1.0	2.9E-01	rs8138938	A	G	-0.03	9.8E-01	-3.3	9.6E-04	0.02	1863	1%	99%	0%	0%	0%
1	*FGGY-DT*	Hemoglobin (HGB)	rs34370185	T	G	7.09	1.4E-12	5.1	4.3E-07	rs34370185	T	G	7.09	1.4E-12	5.1	4.3E-07	1.00	1046	0%	0%	0%	2%	97%
2	*F3*	Hemoglobin (HGB)	rs1146473	C	T	5.82	5.8E-09	0.8	4.5E-01	rs871662	G	A	-1.30	1.9E-01	-3.8	1.5E-04	0.04	1576	0%	94%	0%	5%	1%
3	*ECM1/ADAMTSL4*	Hemoglobin (HGB)	rs4970935	T	C	-13.04	6.1E-39	-0.3	7.8E-01	rs72700806	G	A	-1.81	7.0E-02	3.5	3.9E-04	0.06	1037	0%	88%	0%	12%	0%
4	*AFAP1*	Hemoglobin (HGB)	rs6828005	A	G	-6.67	2.6E-11	3.1	2.2E-03	rs12501350	T	C	2.21	2.7E-02	-3.6	3.5E-04	0.47	2215	0%	93%	0%	2%	5%
5	*ZNF827*	Hemoglobin (HGB)	rs1507928	C	T	5.75	8.9E-09	-0.6	5.2E-01	rs714195	C	T	-0.10	9.2E-01	6.3	2.8E-10	0.01	1242	0%	0%	0%	100%	0%
6	*ITGA1*	Hemoglobin (HGB)	rs73102285	G	A	5.72	1.1E-08	-0.5	6.5E-01	rs60925406	C	T	-1.10	2.7E-01	-6.6	3.0E-11	0.00	2096	0%	0%	0%	100%	0%
7	*PHACTR1*	Hemoglobin (HGB)	rs6925904	G	A	-7.50	6.3E-14	-0.5	6.3E-01	rs62386724	C	T	0.09	9.3E-01	3.4	7.1E-04	0.00	1613	0%	97%	0%	3%	0%
8	*HTRA1*	Hemoglobin (HGB)	rs2736923	A	G	5.47	4.6E-08	0.6	5.4E-01	rs4980169	G	A	0.00	1.0E+00	-4.4	9.2E-06	0.00	1556	0%	71%	0%	29%	0%
9	*SESN3*	Hemoglobin (HGB)	rs11021221	A	T	7.84	4.1E-15	-2.7	6.3E-03	rs12800440	T	C	-0.34	7.3E-01	-6.2	5.7E-10	0.00	1360	0%	0%	0%	100%	0%
10	*LRP1*	Hemoglobin (HGB)	rs11172113	C	T	-11.53	9.0E-31	-3.2	1.2E-03	rs3741414	T	C	-0.64	5.2E-01	9.1	8.4E-20	0.02	999	0%	0%	0%	100%	0%
11	*ATP2B1*	Hemoglobin (HGB)	rs1689040	T	C	-6.17	7.0E-10	-2.0	4.6E-02	rs2615834	A	T	-1.28	2.0E-01	-4.9	1.0E-06	0.00	1024	0%	30%	0%	70%	0%
12	*COL4A1*	Hemoglobin (HGB)	rs7326444	A	G	-6.46	1.0E-10	0.8	4.4E-01	rs79692857	A	C	-0.33	7.4E-01	6.3	2.9E-10	0.00	1148	0%	0%	0%	100%	0%
12	*COL4A2*	Hemoglobin (HGB)	rs11838776	A	G	-8.74	2.5E-18	-3.7	2.2E-04	rs4103	T	C	-0.36	7.2E-01	5.2	1.9E-07	0.06	1225	0%	16%	0%	64%	20%
13	*FBN1*	Hemoglobin (HGB)	rs2437947	A	T	5.94	2.9E-09	-0.6	5.7E-01	rs17462641	T	C	-1.89	5.8E-02	4.9	1.1E-06	0.02	527	0%	6%	0%	94%	0%
14	*THSD4*	Hemoglobin (HGB)	rs10851839	A	T	6.56	5.5E-11	-4.9	9.2E-07	rs2625529	C	G	0.36	7.2E-01	-7.8	8.9E-15	0.00	1139	0%	0%	0%	100%	0%
15	*MRPS6/SLC5A3/KCNE2*	Hemoglobin (HGB)	rs9980618	T	C	-8.35	7.2E-17	0.6	5.7E-01	rs2834317	A	G	2.33	2.0E-02	-12.5	8.0E-36	0.00	1519	0%	0%	0%	100%	0%
16	*TIMP3*	Hemoglobin (HGB)	rs137528	A	G	-5.40	6.8E-08	0.9	3.6E-01	rs9606967	C	G	1.22	2.2E-01	-4.7	3.0E-06	0.00	1864	1%	75%	0%	24%	0%

Supplementary Table 10: Genetic correlation between SCAD and cardiovascular and neurovascular diseases and traits

rg: genetic correlation, se: standard error, P: P-value for the genetic correlation obtained from two-sided Wald test.

CAD: coronary artery disease, MI: myocardial infraction, FMD: fibromuscular dysplasia, CeAD: cervical artery, dissection, IA: intracranial aneurysm, SAH: subarachnoid hemorrhage , uIA: unruptured intracranial aneurysm ,AS: any stoke, AIS: any ischemic stroke, LAS: , CES: cardioembolic stroke, SVS: small vessel stroke, MIG: Migraine, T2D: type 2 diabetes, SMK: smoking (never/ever), SBP: systolic blood pressure, DBP: diastolic blood pressure, PP: pulse pressure, HTN: Hypertension, BMI: body mass index , HDL: high density lipoprotein, LDL: low density lipoprotein, TG: triglycerides, HGB: haemoglobin, NEU : Neutrophil count, MONO : Monocyte count, LYMPHO : Lymphocyte count and PLT: Platelet count

Details on used GWAS summary statistics of diseases and traits are available in Supplementary Table 17

	Unadjusted					SBP mtCOJO					DBP mtCOJO				
Disease or trait	rg	se	L95_rg	U95_rg	P	rg	se	L95_rg	U95_rg	P	rg	se	L95_rg	U95_rg	P
CAD	-0.12	0.04	-0.21	-0.04	3.7E-03	-0.19	0.04	-0.28	-0.11	4.6E-06	-0.19	0.04	-0.27	-0.10	1.3E-05
MI	-0.14	0.05	-0.24	-0.04	6.6E-03	-0.21	0.05	-0.31	-0.10	1.5E-04	-0.21	0.06	-0.32	-0.10	1.6E-04
FMD	0.38	0.18	0.03	0.74	3.5E-02	0.30	0.15	0.00	0.60	5.3E-02	0.30	0.15	0.00	0.60	5.2E-02
CeAD	0.61	0.20	0.21	1.00	2.4E-03	0.52	0.20	0.13	0.92	9.3E-03	0.51	0.20	0.11	0.90	1.2E-02
IA	0.22	0.06	0.10	0.33	2.0E-04	0.16	0.06	0.04	0.27	6.9E-03	0.16	0.06	0.04	0.27	8.2E-03
SAH	0.27	0.07	0.14	0.40	6.4E-05	0.20	0.07	0.07	0.33	2.8E-03	0.20	0.07	0.07	0.33	3.3E-03
uIA	0.14	0.08	-0.02	0.30	9.0E-02	0.09	0.08	-0.07	0.26	2.6E-01	0.09	0.08	-0.08	0.26	2.9E-01
AS	0.17	0.06	0.05	0.29	4.5E-03	0.10	0.06	-0.02	0.22	1.1E-01	0.10	0.06	-0.02	0.23	1.1E-01
AIS	0.17	0.06	0.05	0.28	4.6E-03	0.09	0.06	-0.03	0.22	1.3E-01	0.10	0.06	-0.03	0.22	1.3E-01
LAS	0.68	0.55	-0.39	1.76	2.1E-01	0.46	0.28	-0.08	1.01	9.4E-02	0.51	0.30	-0.07	1.10	8.4E-02
CES	0.34	0.09	0.17	0.51	1.0E-04	0.34	0.09	0.16	0.52	2.0E-04	0.34	0.09	0.16	0.53	2.0E-04
SVS	-0.04	0.10	-0.24	0.16	7.2E-01	-0.09	0.11	-0.31	0.13	4.1E-01	-0.11	0.11	-0.32	0.11	3.2E-01
MIG	0.18	0.06	0.07	0.30	1.3E-03	0.18	0.05	0.08	0.29	5.0E-04	0.16	0.05	0.06	0.27	2.5E-03
T2D	0.02	0.08	-0.15	0.18	8.5E-01	-0.03	0.07	-0.17	0.12	7.3E-01	0.00	0.07	-0.15	0.14	9.5E-01
SMK	0.00	0.03	-0.06	0.06	9.0E-01	0.02	0.03	-0.04	0.08	5.0E-01	0.02	0.03	-0.04	0.07	5.5E-01
SBP	0.12	0.03	0.06	0.19	1.0E-04	-	-	-	-	-	-	-	-	-	-
DBP	0.17	0.03	0.11	0.24	2.6E-07	-	-	-	-	-	-	-	-	-	-
BMI	-0.02	0.03	-0.08	0.03	4.1E-01	-0.02	0.03	-0.07	0.03	4.9E-01	-0.02	0.03	-0.07	0.04	5.3E-01
HDL	-0.09	0.03	-0.15	-0.03	1.8E-03	-0.08	0.03	-0.14	-0.03	2.1E-03	-0.08	0.03	-0.13	-0.03	3.6E-03
LDL	-0.07	0.03	-0.14	0.00	4.5E-02	-0.04	0.03	-0.09	0.02	2.0E-01	-0.04	0.03	-0.10	0.02	1.6E-01
TG	0.06	0.03	-0.01	0.12	8.6E-02	0.04	0.03	-0.02	0.09	2.0E-01	0.03	0.03	-0.02	0.09	2.3E-01
HGB	0.12	0.03	0.06	0.17	2.7E-05	0.08	0.03	0.03	0.14	1.2E-03	0.05	0.03	0.00	0.10	3.9E-02
NEU	0.02	0.03	-0.04	0.08	6.1E-01	-0.01	0.03	-0.07	0.04	6.4E-01	-0.01	0.03	-0.06	0.05	8.2E-01
MONO	0.00	0.03	-0.06	0.07	9.0E-01	-0.01	0.03	-0.06	0.04	7.4E-01	-0.01	0.03	-0.06	0.04	7.4E-01
LYMPHO	-0.05	0.04	-0.13	0.02	1.6E-01	-0.08	0.03	-0.14	-0.02	5.3E-03	-0.08	0.03	-0.14	-0.02	5.6E-03
PLT	-0.06	0.03	-0.11	-0.01	1.4E-02	-0.06	0.02	-0.10	-0.01	1.6E-02	-0.05	0.02	-0.10	-0.01	2.3E-02

Supplementary Table 11: Comparison of SCAD meta-analysis results, unstratified and stratified on FMD status

EA: Effect Allele, OA: Other Allele, OR: Odds Ratio, CI: Confidence Interval, EAF: Effect Allele Frequency, P: P-value of genetic association obtained from two-sided Wald test, Het_P: P-value of heterogeneity obtained from Cochran's Q-test implemented in METAL

CHR	POS	LOCUS	TOP SNP SCAD	EA	OA	Unstratified [1268-1917] Cases / [6896-9291] Controls				SCAD with FMD [203-409] Cases / [3036-4961] Controls				SCAD without FMD [305-614] Cases / [3400-5776] Controls			
						**OR (95% CI)**	**P**	**EAF**	**Het_P**	**OR (95% CI)**	**P**	**EAF**	**Het_P**	**OR (95% CI)**	**P**	**EAF**	**Het_P**
1	59656909	*FGGY-DT*	rs34370185	T	G	1.34 (1.24 - 1.46)	1.4E-12	0.29	0.044	1.23 (1.05 - 1.46)	1.2E-02	0.28	0.396	1.35 (1.18 - 1.55)	1.5E-05	0.29	0.076
1	95050472	*F3*	rs1146473	C	T	1.32 (1.2 - 1.45)	5.8E-09	0.19	0.098	1.31 (1.09 - 1.56)	3.5E-03	0.19	0.256	1.32 (1.13 - 1.55)	4.2E-04	0.19	0.001
1	150504062	*ECM1/ADAMTSL4*	rs4970935	C	T	1.72 (1.59 - 1.87)	6.1E-39	0.28	0.636	1.77 (1.51 - 2.08)	3.7E-12	0.27	0.655	1.78 (1.55 - 2.04)	9.0E-17	0.27	0.808
4	7774352	*AFAP1*	rs6828005	G	A	1.29 (1.2 - 1.4)	2.6E-11	0.45	0.816	1.41 (1.21 - 1.65)	9.6E-06	0.44	0.193	1.25 (1.1 - 1.42)	4.9E-04	0.44	0.009
4	146788035	*ZNF827*	rs1507928	C	T	1.25 (1.16 - 1.35)	8.9E-09	0.48	0.385	1.34 (1.15 - 1.56)	1.8E-04	0.47	0.920	1.25 (1.1 - 1.42)	6.1E-04	0.47	0.305
5	52155642	*ITGA1*	rs73102285	G	A	1.27 (1.17 - 1.38)	1.1E-08	0.27	0.312	1.3 (1.1 - 1.54)	1.8E-03	0.27	0.598	1.36 (1.18 - 1.56)	1.7E-05	0.27	0.377
6	12903957	*PHACTR1*	rs9349379	A	G	1.64 (1.51 - 1.78)	2.9E-32	0.62	0.193	1.67 (1.42 - 1.97)	1.3E-09	0.61	0.244	1.63 (1.42 - 1.87)	6.3E-12	0.61	0.184
10	124259062	*HTRA1*	rs2736923	A	G	1.44 (1.26 - 1.64)	4.6E-08	0.89	0.600	1.53 (1.17 - 2.01)	2.1E-03	0.88	0.488	1.4 (1.13 - 1.73)	2.2E-03	0.88	0.424
11	95308854	*SESN3*	rs11021221	A	T	1.47 (1.33 - 1.61)	4.1E-15	0.17	0.192	1.62 (1.35 - 1.95)	3.1E-07	0.17	0.462	1.3 (1.1 - 1.52)	1.9E-03	0.17	0.320
12	57527283	*LRP1*	rs11172113	T	C	1.62 (1.49 - 1.76)	9.0E-31	0.62	0.702	1.61 (1.36 - 1.9)	1.6E-08	0.61	0.477	1.6 (1.39 - 1.83)	2.6E-11	0.61	0.280
12	89978233	*ATP2B1*	rs1689040	C	T	1.28 (1.18 - 1.39)	7.0E-10	0.59	0.660	1.22 (1.04 - 1.42)	1.3E-02	0.58	0.361	1.3 (1.14 - 1.48)	6.9E-05	0.59	0.612
13	110838236	*COL4A1*	rs7326444	G	A	1.31 (1.21 - 1.42)	1.0E-10	0.64	0.517	1.55 (1.31 - 1.84)	4.2E-07	0.65	0.456	1.26 (1.1 - 1.44)	9.3E-04	0.64	0.974
13	111040681	*COL4A2*	rs11838776	G	A	1.5 (1.37 - 1.65)	2.5E-18	0.73	0.420	1.63 (1.35 - 1.97)	3.2E-07	0.72	0.841	1.54 (1.32 - 1.8)	3.7E-08	0.73	0.890
15	48763754	*FBN1*	rs7174973	G	A	1.54 (1.37 - 1.72)	1.6E-13	0.11	0.030	1.9 (1.52 - 2.37)	1.1E-08	0.11	0.232	1.69 (1.41 - 2.02)	1.1E-08	0.11	0.485
15	71628370	*THSD4*	rs10851839	A	T	1.32 (1.22 - 1.44)	5.5E-11	0.68	0.237	1.34 (1.12 - 1.59)	9.7E-04	0.68	0.142	1.34 (1.17 - 1.55)	4.1E-05	0.67	0.550
21	35593827	*MRPS6/SLC5A3/KCNE2*	rs28451064	G	A	2.04 (1.77 - 2.35)	1.2E-22	0.88	0.497	1.71 (1.29 - 2.26)	1.7E-04	0.87	0.889	2.74 (2.08 - 3.61)	7.0E-13	0.88	0.125
22	33282971	*TIMP3*	rs137507	T	C	1.38 (1.23 - 1.55)	3.3E-08	0.11	0.019	1.38 (1.1 - 1.73)	5.2E-03	0.11	0.065	1.42 (1.17 - 1.71)	3.0E-04	0.11	0.248

Supplementary Table 12: Genetic correlation between SCAD and CAD conditioned on cardiovascular risk factors and blood traits (mtCOJO)

rg: genetic correlation, se: standard error, P: P-value for the genetic correlation obtained from two-sided Wald test.

SBP: systolic blood pressure, DBP: diastolic blood pressure, BMI: body mass index, HDL: high density lipoprotein, LDL: low density lipoprotein, TG: triglycerides, SMK: smoking (never/ever), HGB: haemoglobin

Details on used GWAS summary statistics of exposures are available in Supplementary Table 17

Disease or trait	rg	se	L95_rg	U95_rg	z	P
Unadjusted	-0.122	0.042	-0.20	-0.04	-2.91	3.6E-03
SBP	-0.195	0.043	-0.28	-0.11	-4.58	4.6E-06
DBP	-0.188	0.043	-0.27	-0.10	-4.37	1.3E-05
BMI	-0.128	0.041	-0.21	-0.05	-3.16	1.6E-03
HDL	-0.149	0.041	-0.23	-0.07	-3.66	2.0E-04
LDL	-0.123	0.041	-0.20	-0.04	-3.04	2.4E-03
TG	-0.141	0.041	-0.22	-0.06	-3.46	5.0E-04
SMK	-0.123	0.041	-0.20	-0.04	-3.04	2.4E-03
HGB	-0.127	0.041	-0.21	-0.05	-3.08	2.1E-03

Supplementary Table 13: Mendelian randomization (MR) analysis between SCAD or CAD and cardiovascular risk factors and blood traits

N_SNP : count of SNP used as instrumental variables in the MR analysis, BETA: effect size obtained from MR analysis (IVW: Inverse variance weighted, MR-egger, weighted median), SE: standard error of effect size, P: P-value for the association obtained from two-sided Wald test. P.adj: Bonferroni adjusted P-value after adjusting for 9 traits.

CAD: coronary artery disease, SCAD : spontaneous coronary artery dissection, SBP: systolic blood pressure, DBP: diastolic blood pressure, BMI: body mass index , HDL: high density lipoprotein, LDL: low density lipoprotein, TG: triglycerides, SMK: smoking (never/ever), T2D: type 2 diabete, HGB: haemoglobin,

details on used GWAS summary statistics of exposures are available in Supplementry Table 17

	Outcome = SCAD											Outcome = CAD										
		IVW				MR-Egger			Weighted median				IVW				Egger			Weighted median		
Exposure (risk factor)	N_SNP	BETA	SE	P	P.adj	BETA	SE	P	BETA	SE	P	N_SNP	BETA	SE	P	P.adj	BETA	SE	P	BETA	SE	P
SBP	433	0.05	0.01	7.6E-06	8.4E-05	0.12	0.03	2.1E-05	0.04	0.01	1.8E-03	497	0.04	0.002	8.6E-49	9.5E-48	0.04	0.01	2.8E-09	0.03	0.002	1.9E-50
DBP	444	0.10	0.02	1.9E-08	2.1E-07	0.23	0.05	2.7E-06	0.09	0.02	2.1E-05	510	0.06	0.004	1.6E-44	1.8E-43	0.06	0.01	1.3E-08	0.06	0.004	9.6E-46
BMI	276	0.00	0.03	8.9E-01	1.0E+00	0.21	0.09	2.6E-02	0.03	0.05	4.7E-01	319	0.09	0.01	3.0E-35	3.3E-34	0.08	0.02	4.9E-04	0.10	0.01	5.2E-35
HDL	175	-0.89	0.36	1.4E-02	1.5E-01	-0.01	0.67	9.9E-01	-0.55	0.52	2.9E-01	186	-0.66	0.10	1.8E-10	2.0E-09	-0.50	0.18	5.0E-03	-0.46	0.12	1.1E-04
LDL	98	-0.23	0.22	3.0E-01	1.0E+00	-0.10	0.41	8.0E-01	0.07	0.32	8.2E-01	107	0.64	0.09	7.4E-13	8.1E-12	1.00	0.17	3.2E-08	0.67	0.06	1.3E-25
TG	131	0.22	0.13	9.7E-02	1.0E+00	0.16	0.21	4.6E-01	0.18	0.19	3.5E-01	150	0.31	0.05	3.9E-11	4.3E-10	0.19	0.07	6.5E-03	0.24	0.04	2.8E-10
SMK	28	-0.57	1.09	6.0E-01	1.0E+00	4.81	6.65	4.8E-01	-0.53	1.51	7.2E-01	35	0.37	0.18	4.4E-02	4.8E-01	0.43	0.79	5.9E-01	0.43	0.24	6.7E-02
T2D	153	0.08	0.07	2.7E-01	1.0E+00	0.01	0.20	9.5E-01	0.07	0.10	5.0E-01	162	0.12	0.02	1.6E-10	1.8E-09	0.03	0.04	4.8E-01	0.09	0.02	1.5E-07
HGB	296	0.56	0.14	3.3E-05	3.6E-04	0.39	0.28	1.6E-01	0.53	0.21	1.2E-02	210	0.05	0.04	2.3E-01	1.0E+00	-0.14	0.06	2.6E-02	-0.03	0.04	4.4E-01

Supplementary Table 14: Mendelian randomization (MR) analysis between SCAD or CAD and cardiovascular risk factors and blood traits stratified on sex

N_SNP : count of SNP used as instrumental variables in the MR analysis, BETA: effect size obtained from IVW MR analysis, SE: standard error of effect size, P: P-value for the association obtained from two-sided Wald test.

CAD: coronary artery disease, SCAD : spontaneous coronary artery dissection, SBP: systolic blood pressure, DBP: diastolic blood pressure, BMI: body mass index , HDL: high density lipoprotein, LDL: low density lipoprotein, TG: triglycerides, SMK: smoking (never/ever), T2D: type 2 diabete, HGB: haemoglobin

details on used GWAS summary statistics of exposures are available in Supplementary Table 17

	Women								Men			
	Outcome = SCAD				Outcome = CAD				Outcome = CAD			
**Exposure (risk factor)**	**N_SNP**	**BETA**	**SE**	**P**	**N_SNP**	**BETA**	**SE**	**P**	**N_SNP**	**BETA**	**SE**	**P**
SBP	159	0.04	0.01	5.8E-03	185	0.04	0.004	7.9E-23	174	0.03	0.01	6.3E-11
DBP	137	0.09	0.03	7.7E-04	163	0.06	0.01	6.4E-17	158	0.05	0.01	4.1E-10
BMI	277	0.01	0.03	8.3E-01	333	0.09	0.01	9.9E-26	317	0.10	0.01	8.1E-28
HDL	176	-0.81	0.34	1.8E-02	198	-0.59	0.11	1.1E-07	181	-0.84	0.13	4.0E-10
LDL	100	-0.12	0.21	5.7E-01	109	0.48	0.09	4.4E-08	102	0.69	0.10	4.3E-11
TG	128	0.25	0.15	1.0E-01	153	0.38	0.06	5.3E-11	142	0.26	0.04	2.1E-09
SMK	28	-0.23	1.07	8.3E-01	37	0.44	0.27	1.0E-01	35	0.29	0.22	1.8E-01
T2D	153	0.09	0.07	2.2E-01	161	0.13	0.02	2.1E-09	156	0.12	0.02	2.9E-08
HGB	172	0.53	0.19	5.6E-03	215	0.05	0.05	3.6E-01	200	0.04	0.04	4.3E-01

Supplementary Table 15: **Details of genotyping, pre-imputation and post-imputation quality control steps per study.**
N : number of samples, IBD: Identity by descent , PCA: Principal component analysis.

*UK Biobank controls randomly selected after exclusion for relatedness (K>0.044), heterozygosity and self-reported ethnicity
**Only include UBC samples from CanSCAD
**Only include UBC samples from CanSCAD, exclude U.S
**Age,sex, and ancestry (PC1-PC3) matched controls.

					**QC steps**							
**Country**	**Cohort**	**Status**	**Genotyping array**	**N (genotyped)**	**sub-Cohort selection filter**	**Heterozygosity / Call Rate**	**Relatdness (IBD>0.185)**	**Non European excluded. (PCA analysis)**	**Non SCAD or unknown**	**genetic syndrome cases**	**Sex check**	**N included in GWAS**
FRA	DISCO	Cases	Infinium OmniExpressExome-8v1.3	412	-	12	1	41	45	-	-	313
	3 Cities study	Controls	Illumina-Human660W-Quad v1.0	1487	-	0	0	0	NR	-	-	1487
UK	SCAD-UK Study I	Cases	Sequenced	383				0				383
		Controls*	Affymetrix Axiom UK Biobank array*	1925		0	0	0	NR			1925
UK	SCAD-UK Study II	Cases	Illumina-Infinium Global Screening Array-24 v2.0 +MD	163		20	0	0	0			143
		Controls*	Affymetrix Axiom UK Biobank array*	815		0	0	0	NR			815
USA	MAYO	Cases	Infinium OmniExpress-24 v1.2	506		0	0	0	0			506
		Controls	Infinium HumanHap550, 610, 660; OmniExpress	1549		0	0	0	NR			1549

USA	CanSCAD	Case	illumina-Infinium CoreExome-24v1.1 BeadArray with 607,778 SNP markers (UM_HUNT_Biobank _v1-1_20006200_A)	502	49	1	1	88	2	2	2	357**
	MGI	Controls		13756		0	0	11631	NR			2125***
USA	DEFINE-SCAD Study	Cases	Infinium OmniExpressExome-8v1.6	47		1	0	0	30			16
		Cases	Illumina-Human-Omni-Express-Exome	172		3	0	0	143			26
		Controls	Infinium OmniExpressExome-8v1.6	1		0	0	0	0			1
		Controls	Illumina-Human-Omni-Express-Exome	164		2	6	0	1		3	152
AU	VCCRI I	Cases	Whole Genome Sequencing	88		0	0	0	0			88
		Controls		1127		0	0	0	NR			1127
AU	VCCRI2	Cases	Axiom UK Biobank array-cases Axiom PMDA-controls	91		0	2	4	0			85
		Controls		111		0	0	0	NR			111

Supplementary Table 16: Epigenomic datasets used for annotations of SCAD associated variants.

Tissue enrichment (Supplementary figure S3)				
**File accession**	**File format**	**Assay**	**Biosample term name**	**Links to Download**
ENCFF559GDW	bed narrowPeak	H3K27ac ChIP-seq	spleen	
ENCFF714TUQ	bed narrowPeak	H3K27ac ChIP-seq	gastrocnemius medialis	
ENCFF271OHD	bed narrowPeak	H3K27ac ChIP-seq	body of pancreas	
ENCFF200VYQ	bed narrowPeak	H3K27ac ChIP-seq	upper lobe of left lung	
ENCFF635VBV	bed narrowPeak	H3K27ac ChIP-seq	heart left ventricle	
ENCFF382EMP	bed narrowPeak	H3K27ac ChIP-seq	right lobe of liver	
ENCFF033TXD	bed narrowPeak	H3K27ac ChIP-seq	ascending aorta	
ENCFF267KHM	bed narrowPeak	H3K27ac ChIP-seq	gastrocnemius medialis	
ENCFF057CRD	bed narrowPeak	H3K27ac ChIP-seq	esophagus squamous epithelium	
ENCFF670PZG	bed narrowPeak	H3K27ac ChIP-seq	gastroesophageal sphincter	
ENCFF814RAE	bed narrowPeak	H3K27ac ChIP-seq	Peyer's patch	
ENCFF731GWD	bed narrowPeak	H3K27ac ChIP-seq	body of pancreas	
ENCFF266NWL	bed narrowPeak	H3K27ac ChIP-seq	transverse colon	
ENCFF538QNE	bed narrowPeak	H3K27ac ChIP-seq	adrenal gland	
ENCFF524AKO	bed narrowPeak	H3K27ac ChIP-seq	ascending aorta	
ENCFF231SUE	bed narrowPeak	H3K27ac ChIP-seq	uterus	
ENCFF214NTL	bed narrowPeak	H3K27ac ChIP-seq	spleen	
ENCFF208DJA	bed narrowPeak	H3K27ac ChIP-seq	sigmoid colon	
ENCFF561EZS	bed narrowPeak	H3K27ac ChIP-seq	right atrium auricular region	
ENCFF776YOM	bed narrowPeak	H3K27ac ChIP-seq	gastrocnemius medialis	
ENCFF739QLX	bed narrowPeak	H3K27ac ChIP-seq	tibial nerve	
ENCFF004ANL	bed narrowPeak	H3K27ac ChIP-seq	esophagus muscularis mucosa	
ENCFF703WKZ	bed narrowPeak	H3K27ac ChIP-seq	tibial nerve	
ENCFF394NUN	bed narrowPeak	H3K27ac ChIP-seq	adrenal gland	
ENCFF417GYW	bed narrowPeak	H3K27ac ChIP-seq	thoracic aorta	
ENCFF289XOR	bed narrowPeak	H3K27ac ChIP-seq	skin epidermis	
ENCFF087WUV	bed narrowPeak	H3K27ac ChIP-seq	skin epidermis	
ENCFF489GFM	bed narrowPeak	H3K27ac ChIP-seq	skin epidermis	
ENCFF683QWQ	bed narrowPeak	H3K27ac ChIP-seq	left lung	
ENCFF758DTU	bed narrowPeak	H3K27ac ChIP-seq	spleen	
ENCFF877IEN	bed narrowPeak	H3K27ac ChIP-seq	adrenal gland	
ENCFF317ENJ	bed narrowPeak	H3K27ac ChIP-seq	adrenal gland	
ENCFF770HCQ	bed narrowPeak	H3K27ac ChIP-seq	pancreas	
ENCFF862CEW	bed narrowPeak	H3K27ac ChIP-seq	spleen	
ENCFF875LMK	bed narrowPeak	H3K27ac ChIP-seq	skin epidermis	
ENCFF987KRH	bed narrowPeak	H3K27ac ChIP-seq	skin epidermis	
ENCFF677BHR	bed narrowPeak	H3K27ac ChIP-seq	chorionic villus	

ENCFF101BIB	bed narrowPeak	H3K27ac	ChIP-seq	gastroesophageal sphincter
ENCFF988ZYW	bed narrowPeak	H3K27ac	ChIP-seq	breast epithelium
ENCFF322YIK	bed narrowPeak	H3K27ac	ChIP-seq	spleen
ENCFF111SYA	bed narrowPeak	H3K27ac	ChIP-seq	sigmoid colon
ENCFF787TWQ	bed narrowPeak	H3K27ac	ChIP-seq	gastroesophageal sphincter
ENCFF379RWI	bed narrowPeak	H3K27ac	ChIP-seq	skin epidermis
ENCFF257VPC	bed narrowPeak	H3K27ac	ChIP-seq	stomach
ENCFF285RPL	bed narrowPeak	H3K27ac	ChIP-seq	ovary
ENCFF219SMW	bed narrowPeak	H3K27ac	ChIP-seq	chorionic villus
ENCFF477MXB	bed narrowPeak	H3K27ac	ChIP-seq	placental basal plate
ENCFF105SAZ	bed narrowPeak	H3K27ac	ChIP-seq	temporal lobe
ENCFF722LRM	bed narrowPeak	H3K27ac	ChIP-seq	stomach smooth muscle
ENCFF874GTL	bed narrowPeak	H3K27ac	ChIP-seq	placenta
ENCFF645UIY	bed narrowPeak	H3K27ac	ChIP-seq	chorionic villus
ENCFF286ERH	bed narrowPeak	H3K27ac	ChIP-seq	chorion
ENCFF382PYP	bed narrowPeak	H3K27ac	ChIP-seq	mucosa of rectum
ENCFF177IAO	bed narrowPeak	H3K27ac	ChIP-seq	trophoblast
ENCFF398EEO	bed narrowPeak	H3K27ac	ChIP-seq	suprapubic skin
ENCFF310DII	bed narrowPeak	H3K27ac	ChIP-seq	Peyer's patch
ENCFF337KNQ	bed narrowPeak	H3K27ac	ChIP-seq	coronary artery
ENCFF230KBG	bed narrowPeak	H3K27ac	ChIP-seq	skin epidermis
ENCFF860TAY	bed narrowPeak	H3K27ac	ChIP-seq	middle frontal area 46
ENCFF169QSG	bed narrowPeak	H3K27ac	ChIP-seq	middle frontal area 46
ENCFF165SPP	bed narrowPeak	H3K27ac	ChIP-seq	vagina
ENCFF423CQD	bed narrowPeak	H3K27ac	ChIP-seq	skin epidermis
ENCFF337DEA	bed narrowPeak	H3K27ac	ChIP-seq	middle frontal area 46
ENCFF899KVD	bed narrowPeak	H3K27ac	ChIP-seq	spleen
ENCFF507WEL	bed narrowPeak	H3K27ac	ChIP-seq	breast epithelium
ENCFF730HDE	bed narrowPeak	H3K27ac	ChIP-seq	middle frontal area 46
ENCFF078CLH	bed narrowPeak	H3K27ac	ChIP-seq	thoracic aorta
ENCFF595JKW	bed narrowPeak	H3K27ac	ChIP-seq	middle frontal area 46
ENCFF280JLL	bed narrowPeak	H3K27ac	ChIP-seq	adrenal gland
ENCFF970DMJ	bed narrowPeak	H3K27ac	ChIP-seq	right atrium auricular region
ENCFF007OIY	bed narrowPeak	H3K27ac	ChIP-seq	heart left ventricle
ENCFF797XLL	bed narrowPeak	H3K27ac	ChIP-seq	heart right ventricle
ENCFF014ZMR	bed narrowPeak	H3K27ac	ChIP-seq	sigmoid colon
ENCFF751RTT	bed narrowPeak	H3K27ac	ChIP-seq	thyroid gland
ENCFF910HDI	bed narrowPeak	H3K27ac	ChIP-seq	stomach
ENCFF577QNA	bed narrowPeak	H3K27ac	ChIP-seq	vagina
ENCFF987GGX	bed narrowPeak	H3K27ac	ChIP-seq	body of pancreas
ENCFF283HUT	bed narrowPeak	H3K27ac	ChIP-seq	middle frontal area 46
ENCFF270GEZ	bed narrowPeak	H3K27ac	ChIP-seq	thyroid gland

ENCFF336ETH	bed narrowPeak	H3K27ac ChIP-seq	heart left ventricle
ENCFF945XND	bed narrowPeak	H3K27ac ChIP-seq	tibial artery
ENCFF933DUA	bed narrowPeak	H3K27ac ChIP-seq	middle frontal area 46
ENCFF736SWI	bed narrowPeak	H3K27ac ChIP-seq	stomach
ENCFF108SAD	bed narrowPeak	H3K27ac ChIP-seq	uterus
ENCFF216XRU	bed narrowPeak	H3K27ac ChIP-seq	middle frontal area 46
ENCFF124JXP	bed narrowPeak	H3K27ac ChIP-seq	middle frontal area 46
ENCFF448PFH	bed narrowPeak	H3K27ac ChIP-seq	lower lobe of left lung
ENCFF346GZD	bed narrowPeak	H3K27ac ChIP-seq	skin epidermis
ENCFF924OGR	bed narrowPeak	H3K27ac ChIP-seq	skin epidermis
ENCFF600QTP	bed narrowPeak	H3K27ac ChIP-seq	heart right ventricle
ENCFF045ADI	bed narrowPeak	H3K27ac ChIP-seq	middle frontal area 46
ENCFF714JDQ	bed narrowPeak	H3K27ac ChIP-seq	pancreas
ENCFF721ZGP	bed narrowPeak	H3K27ac ChIP-seq	middle frontal area 46
ENCFF462XVH	bed narrowPeak	H3K27ac ChIP-seq	skin epidermis
ENCFF861YME	bed narrowPeak	H3K27ac ChIP-seq	middle frontal area 46
ENCFF654QRM	bed narrowPeak	H3K27ac ChIP-seq	body of pancreas
ENCFF114SOB	bed narrowPeak	H3K27ac ChIP-seq	esophagus muscularis mucosa
ENCFF882VLE	bed narrowPeak	H3K27ac ChIP-seq	upper lobe of left lung
ENCFF003TGC	bed narrowPeak	H3K27ac ChIP-seq	skin epidermis
ENCFF214YFB	bed narrowPeak	H3K27ac ChIP-seq	heart left ventricle
ENCFF229NLK	bed narrowPeak	H3K27ac ChIP-seq	middle frontal area 46
ENCFF847EPP	bed narrowPeak	H3K27ac ChIP-seq	esophagus squamous epithelium
ENCFF024XNY	bed narrowPeak	H3K27ac ChIP-seq	middle frontal area 46
ENCFF712CHB	bed narrowPeak	H3K27ac ChIP-seq	middle frontal area 46
ENCFF528AIU	bed narrowPeak	H3K27ac ChIP-seq	middle frontal area 46
ENCFF496RDA	bed narrowPeak	H3K27ac ChIP-seq	upper lobe of left lung
ENCFF253VVF	bed narrowPeak	H3K27ac ChIP-seq	esophagus squamous epithelium
ENCFF032CSL	bed narrowPeak	H3K27ac ChIP-seq	testis
ENCFF474VLR	bed narrowPeak	H3K27ac ChIP-seq	middle frontal area 46
ENCFF581YQU	bed narrowPeak	H3K27ac ChIP-seq	middle frontal area 46
ENCFF960JWJ	bed narrowPeak	H3K27ac ChIP-seq	esophagus muscularis mucosa
ENCFF549AXK	bed narrowPeak	H3K27ac ChIP-seq	colonic mucosa
ENCFF189UQY	bed narrowPeak	H3K27ac ChIP-seq	middle frontal area 46
ENCFF380YWT	bed narrowPeak	H3K27ac ChIP-seq	heart left ventricle
ENCFF283LVU	bed narrowPeak	H3K27ac ChIP-seq	middle frontal area 46
ENCFF693JGW	bed narrowPeak	H3K27ac ChIP-seq	middle frontal area 46
ENCFF017NRE	bed narrowPeak	H3K27ac ChIP-seq	gastroesophageal sphincter
ENCFF381SFJ	bed narrowPeak	H3K27ac ChIP-seq	upper lobe of left lung
ENCFF756JDB	bed narrowPeak	H3K27ac ChIP-seq	middle frontal area 46
ENCFF521HKS	bed narrowPeak	H3K27ac ChIP-seq	heart right ventricle
ENCFF110LKJ	bed narrowPeak	H3K27ac ChIP-seq	parathyroid adenoma

ENCFF010VMY	bed narrowPeak	H3K27ac ChIP-seq	middle frontal area 46	
ENCFF089YVE	bed narrowPeak	H3K27ac ChIP-seq	middle frontal area 46	
ENCFF300SDH	bed narrowPeak	H3K27ac ChIP-seq	middle frontal area 46	
ENCFF570DDI	bed narrowPeak	H3K27ac ChIP-seq	heart right ventricle	
ENCFF319KZD	bed narrowPeak	H3K27ac ChIP-seq	middle frontal area 46	
ENCFF717AYR	bed narrowPeak	H3K27ac ChIP-seq	tibial nerve	
ENCFF761QUK	bed narrowPeak	H3K27ac ChIP-seq	middle frontal area 46	
ENCFF936SMV	bed narrowPeak	H3K27ac ChIP-seq	Peyer's patch	
ENCFF540YUQ	bed narrowPeak	H3K27ac ChIP-seq	testis	
ENCFF970PFD	bed narrowPeak	H3K27ac ChIP-seq	middle frontal area 46	
ENCFF749JEY	bed narrowPeak	H3K27ac ChIP-seq	heart right ventricle	
ENCFF142COP	bed narrowPeak	H3K27ac ChIP-seq	transverse colon	
ENCFF487FOU	bed narrowPeak	H3K27ac ChIP-seq	adrenal gland	
ENCFF128AQO	bed narrowPeak	H3K27ac ChIP-seq	middle frontal area 46	
ENCFF787FST	bed narrowPeak	H3K27ac ChIP-seq	skin epidermis	https://www.encodeproject.org/
ENCFF240EGF	bed narrowPeak	H3K27ac ChIP-seq	skin epidermis	
ENCFF499HFY	bed narrowPeak	H3K27ac ChIP-seq	heart left ventricle	
ENCFF579OOI	bed narrowPeak	H3K27ac ChIP-seq	Peyer's patch	
ENCFF600DLN	bed narrowPeak	H3K27ac ChIP-seq	skin epidermis	
ENCFF895PGR	bed narrowPeak	H3K27ac ChIP-seq	skin epidermis	
ENCFF441AHV	bed narrowPeak	H3K27ac ChIP-seq	tibial nerve	
ENCFF676LJR	bed narrowPeak	H3K27ac ChIP-seq	middle frontal area 46	
ENCFF523WDP	bed narrowPeak	H3K27ac ChIP-seq	tibial artery	
ENCFF205LFV	bed narrowPeak	H3K27ac ChIP-seq	middle frontal area 46	
ENCFF978AOD	bed narrowPeak	H3K27ac ChIP-seq	stomach	
ENCFF294GNO	bed narrowPeak	H3K27ac ChIP-seq	transverse colon	
ENCFF673PCP	bed narrowPeak	H3K27ac ChIP-seq	esophagus squamous epithelium	
ENCFF639KHG	bed narrowPeak	H3K27ac ChIP-seq	skin epidermis	
ENCFF805FFP	bed narrowPeak	H3K27ac ChIP-seq	spleen	
ENCFF913SWW	bed narrowPeak	H3K27ac ChIP-seq	heart right ventricle	
ENCFF065ZDX	bed narrowPeak	H3K27ac ChIP-seq	thyroid gland	
ENCFF984YUQ	bed narrowPeak	H3K27ac ChIP-seq	coronary artery	
ENCFF457YRY	bed narrowPeak	H3K27ac ChIP-seq	placental basal plate	
ENCFF095OXH	bed narrowPeak	H3K27ac ChIP-seq	heart left ventricle	
ENCFF083HKS	bed narrowPeak	H3K27ac ChIP-seq	skin epidermis	
ENCFF551MWX	bed narrowPeak	H3K27ac ChIP-seq	prostate gland	
ENCFF087FDQ	bed narrowPeak	H3K27ac ChIP-seq	gastrocnemius medialis	
ENCFF368OSK	bed narrowPeak	H3K27ac ChIP-seq	transverse colon	
ENCFF414SKU	bed narrowPeak	H3K27ac ChIP-seq	sigmoid colon	
ENCFF594VVB	bed narrowPeak	H3K27ac ChIP-seq	skin epidermis	
ENCFF143DRH	bed narrowPeak	H3K27ac ChIP-seq	skin epidermis	
ENCFF138WNV	bed narrowPeak	H3K27ac ChIP-seq	thyroid gland	

ENCFF498SSN	bed narrowPeak	H3K27ac ChIP-seq	pancreas
ENCFF788DLD	bed narrowPeak	H3K27ac ChIP-seq	middle frontal area 46
ENCFF610VHP	bed narrowPeak	H3K27ac ChIP-seq	pancreas
ENCFF245RGA	bed narrowPeak	H3K27ac ChIP-seq	heart right ventricle
ENCFF034GZW	bed narrowPeak	H3K27ac ChIP-seq	skeletal muscle tissue
ENCFF173ZPC	bed narrowPeak	H3K27ac ChIP-seq	mucosa of rectum
ENCFF937WTH	bed narrowPeak	H3K27ac ChIP-seq	caudate nucleus
ENCFF737HGQ	bed narrowPeak	H3K27ac ChIP-seq	kidney
ENCFF236DDT	bed narrowPeak	H3K27ac ChIP-seq	temporal lobe
ENCFF066PAQ	bed narrowPeak	H3K27ac ChIP-seq	layer of hippocampus
ENCFF703WCD	bed narrowPeak	H3K27ac ChIP-seq	middle frontal area 46
ENCFF056SCA	bed narrowPeak	H3K27ac ChIP-seq	rectal smooth muscle tissue
ENCFF089APD	bed narrowPeak	H3K27ac ChIP-seq	colonic mucosa
ENCFF778VTG	bed narrowPeak	H3K27ac ChIP-seq	placenta
ENCFF745JHJ	bed narrowPeak	H3K27ac ChIP-seq	trophoblast
ENCFF016AAS	bed narrowPeak	H3K27ac ChIP-seq	skin epidermis
ENCFF014OZD	bed narrowPeak	H3K27ac ChIP-seq	lower lobe of left lung
ENCFF729IPL	bed narrowPeak	H3K27ac ChIP-seq	skin epidermis
ENCFF925RBS	bed narrowPeak	H3K27ac ChIP-seq	middle frontal area 46
ENCFF658NHX	bed narrowPeak	H3K27ac ChIP-seq	subcutaneous abdominal adipose tissue
ENCFF552UIM	bed narrowPeak	H3K27ac ChIP-seq	chorionic villus
ENCFF805YRQ	bed narrowPeak	H3K27ac ChIP-seq	liver
ENCFF155FWO	bed narrowPeak	H3K27ac ChIP-seq	middle frontal area 46
ENCFF201VZW	bed narrowPeak	H3K27ac ChIP-seq	prostate gland
ENCFF275GAS	bed narrowPeak	H3K27ac ChIP-seq	large intestine
ENCFF982LEG	bed narrowPeak	H3K27ac ChIP-seq	esophagus muscularis mucosa
ENCFF341BPG	bed narrowPeak	H3K27ac ChIP-seq	colonic mucosa
ENCFF150MNN	bed narrowPeak	H3K27ac ChIP-seq	layer of hippocampus
ENCFF551LSF	bed narrowPeak	H3K27ac ChIP-seq	layer of hippocampus
ENCFF857JHM	bed narrowPeak	H3K27ac ChIP-seq	muscle layer of duodenum
ENCFF516MFW	bed narrowPeak	H3K27ac ChIP-seq	spinal cord
ENCFF659NCA	bed narrowPeak	H3K27ac ChIP-seq	endocrine pancreas
ENCFF340JNX	bed narrowPeak	H3K27ac ChIP-seq	amnion
ENCFF062NKK	bed narrowPeak	H3K27ac ChIP-seq	pancreas
ENCFF592GQB	bed narrowPeak	H3K27ac ChIP-seq	esophagus
ENCFF066ROS	bed narrowPeak	H3K27ac ChIP-seq	stomach
ENCFF153OUB	bed narrowPeak	H3K27ac ChIP-seq	right cardiac atrium
ENCFF287VIA	bed narrowPeak	H3K27ac ChIP-seq	liver
ENCFF906EQK	bed narrowPeak	H3K27ac ChIP-seq	heart right ventricle
ENCFF718QPZ	bed narrowPeak	H3K27ac ChIP-seq	thymus
ENCFF859UCD	bed narrowPeak	H3K27ac ChIP-seq	muscle of trunk
ENCFF618CUQ	bed narrowPeak	H3K27ac ChIP-seq	small intestine

ENCFF466EWZ	bed narrowPeak	H3K27ac ChIP-seq	muscle layer of colon
ENCFF208GHP	bed narrowPeak	H3K27ac ChIP-seq	urinary bladder
ENCFF450OXP	bed narrowPeak	H3K27ac ChIP-seq	pancreas
ENCFF672SGH	bed narrowPeak	H3K27ac ChIP-seq	middle frontal area 46
ENCFF610WJV	bed narrowPeak	H3K27ac ChIP-seq	heart left ventricle
ENCFF401XVC	bed narrowPeak	H3K27ac ChIP-seq	psoas muscle
ENCFF824TUN	bed narrowPeak	H3K27ac ChIP-seq	heart right ventricle
ENCFF445XCF	bed narrowPeak	H3K27ac ChIP-seq	esophagus
ENCFF329CDU	bed narrowPeak	H3K27ac ChIP-seq	heart right ventricle
ENCFF037RUM	bed narrowPeak	H3K27ac ChIP-seq	heart left ventricle
ENCFF231VWX	bed narrowPeak	H3K27ac ChIP-seq	sigmoid colon
ENCFF346BOQ	bed narrowPeak	H3K27ac ChIP-seq	adrenal gland
ENCFF654RND	bed narrowPeak	H3K27ac ChIP-seq	heart left ventricle
ENCFF872ZJD	bed narrowPeak	H3K27ac ChIP-seq	middle frontal area 46
ENCFF129DPW	bed narrowPeak	H3K27ac ChIP-seq	adipose tissue
ENCFF994ZGB	bed narrowPeak	H3K27ac ChIP-seq	aorta
ENCFF145NKN	bed narrowPeak	H3K27ac ChIP-seq	spleen
ENCFF372OTI	bed narrowPeak	H3K27ac ChIP-seq	stomach
ENCFF231NZU	bed narrowPeak	H3K27ac ChIP-seq	adrenal gland
ENCFF154ZCF	bed narrowPeak	H3K27ac ChIP-seq	muscle of leg
ENCFF872XZM	bed narrowPeak	H3K27ac ChIP-seq	aorta
ENCFF340SAM	bed narrowPeak	H3K27ac ChIP-seq	left ventricle myocardium inferior
ENCFF954CRD	bed narrowPeak	H3K27ac ChIP-seq	middle frontal area 46
ENCFF059ERB	bed narrowPeak	H3K27ac ChIP-seq	heart left ventricle
ENCFF668BZJ	bed narrowPeak	H3K27ac ChIP-seq	thymus
ENCFF023HKA	bed narrowPeak	H3K27ac ChIP-seq	psoas muscle
ENCFF311BUM	bed narrowPeak	H3K27ac ChIP-seq	middle frontal area 46
ENCFF610ZPW	bed narrowPeak	H3K27ac ChIP-seq	middle frontal area 46
ENCFF307QYO	bed narrowPeak	H3K27ac ChIP-seq	middle frontal area 46
ENCFF104GTG	bed narrowPeak	H3K27ac ChIP-seq	heart left ventricle
ENCFF442MTS	bed narrowPeak	H3K27ac ChIP-seq	heart right ventricle
ENCFF646VQX	bed narrowPeak	H3K27ac ChIP-seq	parathyroid adenoma
ENCFF681YKY	bed narrowPeak	H3K27ac ChIP-seq	middle frontal area 46
ENCFF846LAZ	bed narrowPeak	H3K27ac ChIP-seq	left lung
ENCFF927GAF	bed narrowPeak	H3K27ac ChIP-seq	spleen
ENCFF745ROB	bed narrowPeak	H3K27ac ChIP-seq	middle frontal area 46
ENCFF508PQT	bed narrowPeak	H3K27ac ChIP-seq	middle frontal area 46
ENCFF272ROP	bed narrowPeak	H3K27ac ChIP-seq	middle frontal area 46
ENCFF384SYD	bed narrowPeak	H3K27ac ChIP-seq	middle frontal area 46
ENCFF595CLG	bed narrowPeak	H3K27ac ChIP-seq	middle frontal area 46
ENCFF297NSU	bed narrowPeak	H3K27ac ChIP-seq	placenta
ENCFF922GGA	bed narrowPeak	H3K27ac ChIP-seq	duodenal mucosa

ENCFF444CZR	bed narrowPeak	H3K27ac ChIP-seq	caudate nucleus
ENCFF418XDA	bed narrowPeak	H3K27ac ChIP-seq	kidney
ENCFF072GKG	bed narrowPeak	H3K27ac ChIP-seq	angular gyrus
ENCFF193GDV	bed narrowPeak	H3K27ac ChIP-seq	liver
ENCFF100WAF	bed narrowPeak	H3K27ac ChIP-seq	cingulate gyrus
ENCFF650YIZ	bed narrowPeak	H3K27ac ChIP-seq	cingulate gyrus
ENCFF976WTG	bed narrowPeak	H3K27ac ChIP-seq	endocrine pancreas
ENCFF008PSH	bed narrowPeak	H3K27ac ChIP-seq	stomach
ENCFF860MVH	bed narrowPeak	H3K27ac ChIP-seq	middle frontal area 46
ENCFF791TAO	bed narrowPeak	H3K27ac ChIP-seq	substantia nigra
ENCFF026YOC	bed narrowPeak	H3K27ac ChIP-seq	chorion
ENCFF684COM	bed narrowPeak	H3K27ac ChIP-seq	angular gyrus
ENCFF273NFQ	bed narrowPeak	H3K27ac ChIP-seq	spleen
ENCFF761PXE	bed narrowPeak	H3K27ac ChIP-seq	adrenal gland
ENCFF574FYX	bed narrowPeak	H3K27ac ChIP-seq	small intestine
ENCFF328KKO	bed narrowPeak	H3K27ac ChIP-seq	ovary
ENCFF464RXV	bed narrowPeak	H3K27ac ChIP-seq	psoas muscle
ENCFF015DHA	bed narrowPeak	H3K27ac ChIP-seq	spleen
ENCFF759DJM	bed narrowPeak	H3K27ac ChIP-seq	sigmoid colon
ENCFF965GSQ	bed narrowPeak	H3K27ac ChIP-seq	stomach
ENCFF898XDK	bed narrowPeak	H3K27ac ChIP-seq	small intestine
ENCFF074WDV	bed narrowPeak	H3K27ac ChIP-seq	small intestine
ENCFF314QTE	bed narrowPeak	H3K27ac ChIP-seq	lung
ENCFF652JPJ	bed narrowPeak	H3K27ac ChIP-seq	lung

snATAC-Seq enrichment (Figure 2, Supplementary Figure 4)

Cell type	File format	Type	closest Cell Ontology term(s)	Link to download
Fibroblast (General)	bed	snATAC-Seq	fibroblast	
Fibroblast (Epithelial)	bed	snATAC-Seq	skin fibroblast	
Fibroblast (Gastrointestinal)	bed	snATAC-Seq	fibroblast	
Adipocyte	bed	snATAC-Seq	fat cell	
Mesothelial Cell	bed	snATAC-Seq	mesothelial cell	
Fibroblast (Peripheral Nerve)	bed	snATAC-Seq	fibroblast	
Fibroblast (Sk Muscle Associated)	bed	snATAC-Seq	skeletal muscle fibroblast	
Satellite Cell	bed	snATAC-Seq	skeletal muscle satellite cell	
Fibroblast (Liver Adrenal)	bed	snATAC-Seq	fibroblast	
Thyroid Follicular Cell	bed	snATAC-Seq	thyroid follicular cell	
Pancreatic Acinar Cell	bed	snATAC-Seq	pancreatic acinar cell	
T Lymphocyte 1 (CD8+)	bed	snATAC-Seq	CD8-positive, alpha-beta T cell	
T lymphocyte 2 (CD4+)	bed	snATAC-Seq	CD4-positive, alpha-beta T cell	
Natural Killer T Cell	bed	snATAC-Seq	mature NK T cell	
Naive T cell	bed	snATAC-Seq	naive t cell	
Cardiac Pericyte 1	bed	snATAC-Seq	pericyte cell	

Pericyte (General) 1	bed	snATAC-Seq	pericyte cell	
Pericyte (General) 2	bed	snATAC-Seq	pericyte cell	
Pericyte (General) 3	bed	snATAC-Seq	pericyte cell	
Cardiac Pericyte 2	bed	snATAC-Seq	pericyte cell	
Pericyte (Esophageal Muscularis)	bed	snATAC-Seq	pericyte cell	
Pericyte (General) 4	bed	snATAC-Seq	pericyte cell	
Cardiac Pericyte 3	bed	snATAC-Seq	pericyte cell	
Cardiac Pericyte 4	bed	snATAC-Seq	pericyte cell	
Esophageal Epithelial Cell	bed	snATAC-Seq	non keratinizing barrier epithelial cell	
Pancreatic Beta Cell 1	bed	snATAC-Seq	type B pancreatic cell	
Pancreatic Alpha Cell 1	bed	snATAC-Seq	pancreatic A cell	
Pancreatic Beta Cell 2	bed	snATAC-Seq	type B pancreatic cell	
Pancreatic Delta,Gamma cell	bed	snATAC-Seq	pancreatic D cell, pancreatic PP cell	
Pancreatic Alpha Cell 2	bed	snATAC-Seq	pancreatic A cell	
Gastric Neuroendocrine Cell	bed	snATAC-Seq	stomach neuroendocrine cell	
Alveolar Type 2 (AT2) Cell	bed	snATAC-Seq	type II pneumocyte	
Alveolar Type 1 (AT1) Cell	bed	snATAC-Seq	type I pneumocyte	
Alverolar Type 2,Immune	bed	snATAC-Seq	type II pneumocyte	
Club Cell	bed	snATAC-Seq	club cell	
Cilliated Cell	bed	snATAC-Seq	ciliated cell	
Mammary Luminal Epithelial Cell 1	bed	snATAC-Seq	luminal epithelial cell of mammary glaı	
Basal Epithelial (Mammary)	bed	snATAC-Seq	basal cell	
Granular Epidermal (Skin)	bed	snATAC-Seq	granular cell of epidermis	
Mammary Luminal Epithelial Cell 2	bed	snATAC-Seq	luminal epithelial cell of mammary glaı	
Eccrine Epidermal (Skin)	bed	snATAC-Seq	eccrine cell	
Airway Goblet Cell	bed	snATAC-Seq	respiratory goblet cell	
Basal Epidermal (Skin)	bed	snATAC-Seq	basal cell of epidermis	
Chief Cell	bed	snATAC-Seq	peptic cell	
Parietal Cell	bed	snATAC-Seq	parietal cell	
Foveolar Cell	bed	snATAC-Seq	foveolar cell of stomach	
Schwann Cell (General)	bed	snATAC-Seq	schwann cell	
Melanocyte	bed	snATAC-Seq	melanocyte	
Macrophage (General)	bed	snATAC-Seq	macrophage	
Macrophage (General,Alveolar)	bed	snATAC-Seq	alveolar macrophage	
Microglia	bed	snATAC-Seq	microglial cell	
Glutamatergic Neuron 1	bed	snATAC-Seq	glutamatergic neuron	
Glutamatergic Neuron 2	bed	snATAC-Seq	glutamatergic neuron	
GABAergic Neuron 1	bed	snATAC-Seq	gabaergic neuron	
GABAergic Neuron 2	bed	snATAC-Seq	gabaergic neuron	
CNS,Enteric Neuron	bed	snATAC-Seq	enteric neuron	http://renlab.sdsc.edu/kai/Key_Processed_Data/Peaks/
Keratinocyte 1	bed	snATAC-Seq	keratinocyte	
Keratinocyte 2	bed	snATAC-Seq	keratinocyte	

Transitional Zone Cortical Cell	bed	snATAC-Seq	cortical cell of adrenal gland
Zona Fasciculata Cortical Cell	bed	snATAC-Seq	type II cell of adrenal cortex
Zona Glomerulosa Cortical Cell	bed	snATAC-Seq	type I cell of adrenal cortex
Cortical Epithelial-like	bed	snATAC-Seq	cortical cell of adrenal gland
Mammary Epithelial	bed	snATAC-Seq	mammary gland epithelial cell
Myoepithelial (Skin)	bed	snATAC-Seq	myoepithelial cell
Luteal Cell (Ovarian)	bed	snATAC-Seq	luteal cell
Plasma Cell	bed	snATAC-Seq	plasma cell
Memory B Cell	bed	snATAC-Seq	memory b cell
Hepatocyte	bed	snATAC-Seq	hepatocyte
Oligodendrocyte Precursor	bed	snATAC-Seq	oligodendrocyte precursor cell
Astrocyte 1	bed	snATAC-Seq	astrocyte
Astrocyte 2	bed	snATAC-Seq	astrocyte
Ductal Cell (Pancreatic)	bed	snATAC-Seq	pancreatic ductal cell
Mast Cell	bed	snATAC-Seq	mast cell
Ventricular Cardiomyocyte	bed	snATAC-Seq	ventricular cardiac muscle cell
Atrial Cardiomyocyte	bed	snATAC-Seq	regular atrial cardiac myocyte
Oligodendrocyte	bed	snATAC-Seq	oligodendrocyte
Endothelial Cell (General) 1	bed	snATAC-Seq	endothelial cell
Endothelial Cell (Myocardial)	bed	snATAC-Seq	cardiac endothelial cell
Lymphatic Endothelial Cell	bed	snATAC-Seq	endothelial cell of lymphatic vessel
Endothelial Cell (General) 2	bed	snATAC-Seq	endothelial cell
Alveolar Capillary Endothelial Cell	bed	snATAC-Seq	endothelial cell
Endothelial Cell (General) 3	bed	snATAC-Seq	endothelial cell
Endocardial Cell	bed	snATAC-Seq	endocardial cell
Endothelial (Exocrine Tissues)	bed	snATAC-Seq	endothelial cell
Blood Brain Barrier Endothelial Cell	bed	snATAC-Seq	endothelial cell
Smooth Muscle (Esophageal Muscul	bed	snATAC-Seq	smooth muscle cell of the esophagus
Smooth Muscle (Vaginal)	bed	snATAC-Seq	smooth muscle cell
Smooth Muscle (Esophageal Mucosa	bed	snATAC-Seq	smooth muscle cell
Smooth Muscle (Colon) 1	bed	snATAC-Seq	enteric smooth muscle cell
Smooth Muscle (Esophageal Muscul	bed	snATAC-Seq	smooth muscle cell of the esophagus
Smooth Muscle (Uterine)	bed	snATAC-Seq	uterine smooth muscle cell
Smooth Muscle (General)	bed	snATAC-Seq	smooth muscle cell
Smooth Muscle (GE Junction)	bed	snATAC-Seq	smooth muscle cell
Smooth Muscle (Colon) 2	bed	snATAC-Seq	enteric smooth muscle cell
Smooth Muscle (Esophageal Muscul	bed	snATAC-Seq	smooth muscle cell
Smooth Muscle (General Gastrointe	bed	snATAC-Seq	smooth muscle cell
Type I Skeletal Myocyte	bed	snATAC-Seq	type I muscle cell
Type II Skeletal Myocyte	bed	snATAC-Seq	type II muscle cell
Vascular Smooth Muscle 1	bed	snATAC-Seq	blood vessel smooth muscle cell
Vascular Smooth Muscle 2	bed	snATAC-Seq	blood vessel smooth muscle cell

Cardiac Fibroblasts	bed	snATAC-Seq	fibroblast of cardiac tissue	
Peripheral Nerve Stromal	bed	snATAC-Seq	stromal cell	
Colon Epithelial Cell 1	bed	snATAC-Seq	colon epithelial cell	
Small Intestinal Enterocyte	bed	snATAC-Seq	enterocyte of epithelium of small intest	
Colonic Goblet Cell	bed	snATAC-Seq	colon goblet cell	
Small Intestinal Goblet Cell	bed	snATAC-Seq	small intestine goblet cell	
Colon Epithelial Cell 2	bed	snATAC-Seq	colon epithelial cell	
Colon Epithelial Cell 3	bed	snATAC-Seq	colon epithelial cell	
Enterochromaffin Cell	bed	snATAC-Seq	enterochromaffin-like cell	
Tuft Cell	bed	snATAC-Seq	intestinal tuft cell	
Paneth Cell	bed	snATAC-Seq	paneth cell	
**Additional datasets for SNP annotation (Supplementary Figure 5, Supplementary Table 4)**				
Coronary artery-1	fastq	ATAC-Seq	coronary artery	https://www.ncbi.nlm.nih.gov/sra/?term=SRR2378591
Coronary artery-2	fastq	ATAC-Seq	coronary artery	https://www.ncbi.nlm.nih.gov/sra/?term=SRR2378592
Coronary artery-3	fastq	ATAC-Seq	coronary artery	https://www.ncbi.nlm.nih.gov/sra/?term=SRR2378593
scATAC	fragments.bed	snATAC-Seq	25 tissues	https://www.ncbi.nlm.nih.gov/geo/query/acc.cgi?acc=GSE184462

Supplementary Table 17: details of summary statistics used in to calculate the genetic correlation with SCAD

for continuous traits Neff= sample size of study

for binary traits Neff was calculated as follow : neff= 4/(1/Ncases+1/Nctrls)

* : range.

Trait	abreveation	Type	Strata	Neff	N Cases	N controls	Consortia / study	study reference	Source of summary statistics
Coronary Artery Disease	CAD	binary	Bothsex, Female and male	613021	181522	984168	CARDIoGRAMplusC4D Consortium (Aragam et al)	https://doi.org/10.1101/2021.05.24.21257377	shared by authors
Myocardial Infarction	MI	binary	bothsex	126612	42561	123504	CARDIoGRAMplusC4D Consortium (Nikpay et al)	PMID: 26343387	http://www.cardiogramplusc4d.org/data-downloads/
Fibromuscular dysplasia	FMD	binary	Bothsex and Female	5105	1556	7100	Georges et al	PMID: 34654805	http://ftp.ebi.ac.uk/pub/databases/gwas/summary_statistics/GCST90026001-GCST90027000/GCST90026612
Cervical Artery Dissection	CeAD	binary	Bothsex	5081	1393	14416	Stéphanie Debette et al	PMID: 25420145	shared by authors
Intracranial Aneurysm	IA	binary	Bothsex	[14576 - 24253]*	7495	71934	Mark K Bakker et al	PMID: 33199917	shared by authors
Subarachnoid Haemorrhage	SAH	binary	Bothsex	[10203 - 17019]*	5140	71934	Mark K Bakker et al	PMID: 33199917	shared by authors
unruptured Intracranial Aneurysm (uIA)	uIA	binary	Bothsex	[5225 - 7721]*	2070	71934	Mark K Bakker et al	PMID: 33199917	shared by authors
Any stroke	AS	binary	Bothsex	147590	40585	406111	MEGASTROKE consortium	PMID:29531354	https://www.megastroke.org/
Any Ischemic Stroke	AIS	binary	Bothsex	126232	34217	406111	MEGASTROKE consortium	PMID:29531354	https://www.megastroke.org/
Large Artery Stroke	LAS	binary	Bothsex	16985	4373	146392	MEGASTROKE consortium	PMID:29531354	https://www.megastroke.org/
Cardioembolic Stroke	CES	binary	Bothsex	27795	7193	204570	MEGASTROKE consortium	PMID:29531354	https://www.megastroke.org/
Small Vessel Stroke	SVS	binary	Bothsex	20958	5386	192662	MEGASTROKE consortium	PMID:29531354	https://www.megastroke.org/
Migraine	MIG	binary	Bothsex, Female and male	41332	10647	350494	UK BioBank Neale lab summary statistics	-	http://www.nealelab.is/uk-biobank
Type 2 diabetes	T2D	binary	Bothsex	272026	74124	824006	Mahajan et al	PMID: 30297969	https://diagram-consortium.org/downloads.html
Smoking (ever)	SMK	binary	Bothsex, Female and male	357132	164638	195068	UK BioBank Neale lab summary statistics	-	http://www.nealelab.is/uk-biobank
Systolic blood pressure	SBP	continuous	Bothsex, Female and male	340159	-	-	UK BioBank Neale lab summary statistics	-	http://www.nealelab.is/uk-biobank
Diastolic blood pressure	DBP	continuous	Bothsex, Female and male	340162	-	-	UK BioBank Neale lab summary statistics	-	http://www.nealelab.is/uk-biobank
Systolic blood pressure	SBP	continuous	Bothsex	[637578 - 745820]*	-	-	Evangelou et al	PMID: 30224653	ftp://ftp.ebi.ac.uk/pub/databases/gwas/summary_statistics/EvangelouE_30224653_GCST006624
Diastolic blood pressure	DBP	continuous	Bothsex	[647307 - 757601]*	-	-	Evangelou et al	PMID: 30224653	ftp://ftp.ebi.ac.uk/pub/databases/gwas/summary_statistics/EvangelouE_30224653_GCST006630

Body mass index	BMI	continuous	Bothsex, Female and male	359983	-	-	UK BioBank Neale lab summary statistics	-	http://www.nealelab.is/uk-biobank
High density	HDL	continuous	Bothsex, Female and male	315133	-	-	UK BioBank Neale lab summary statistics	-	http://www.nealelab.is/uk-biobank
Low density	LDL	continuous	Bothsex, Female and male	343621	-	-	UK BioBank Neale lab summary statistics	-	http://www.nealelab.is/uk-biobank
Trygliceryde	TG	continuous	Bothsex, Female and male	343992	-	-	UK BioBank Neale lab summary statistics	-	http://www.nealelab.is/uk-biobank
Hemoglobin	HGB	continuous	Bothsex	408112	-	-	Vuckovic et al	PMID: 32888494	http://ftp.ebi.ac.uk/pub/databases/gwas/summary_statistics/GCST90002001-GCST90003000/GCST90002384/
Neutrophil count	NEU	continuous	Bothsex	408112	-	-	Vuckovic et al	PMID: 32888494	http://ftp.ebi.ac.uk/pub/databases/gwas/summary_statistics/GCST90002001-GCST90003000/GCST90002398
Monocyte count	MONO	continuous	Bothsex	408112	-	-	Vuckovic et al	PMID: 32888494	http://ftp.ebi.ac.uk/pub/databases/gwas/summary_statistics/GCST90002001-GCST90003000/GCST90002393
Lymphocyte counts	LYMPHO	continuous	Bothsex	408112	-	-	Vuckovic et al	PMID: 32888494	http://ftp.ebi.ac.uk/pub/databases/gwas/summary_statistics/GCST90002001-GCST90003000/GCST90002388
Platelet count	PLT	continuous	Bothsex	408112	-	-	Vuckovic et al	PMID: 32888494	http://ftp.ebi.ac.uk/pub/databases/gwas/summary_statistics/GCST90002001-GCST90003000/GCST90002402

Consortium Authors

DISCO Investigators

Nicolas COMBARET[1,] MD and the DISCO Investigators : Pascal MOTREFF[1], MD, PhD ; Géraud SOUTEYRAND[1], MD ; Edouard GERBAUD[2], MD, PhD ; François DERIMAY[3], MD ; Sara BOUAJILA[4], MD; Stéphane MANZO-SILBERMAN[4], MD ; Grégoire RANGE[5], MD ; Nicolas MENEVEAU[6], MD, PhD ; Brahim HARBAOUI[7], MD, PhD ; Benoit LATTUCA[8], MD, PhD ; Didier BRESSON[9], MD ; Lionel MANGIN[10], MD ; Thibault LHERMUSIER[11], MD ; Emmanuel BOIFFARD[12]MD ; Emmanuelle FILIPPI[13], MD ; Vincent ROULE[14], MD, PhD; Jean-Louis GEORGES[15], MD ; Arnaud FLUTTAZ[16], MD ; Stéphanie MARLIERE[17]

1: Department of cardiology, CHU Clermont-Ferrand, CNRS, Université Clermont Auvergne, Clermont-Ferrand, France
2: Cardiology Intensive Care Unit and Interventional Cardiology, Hôpital Cardiologique du Haut Lévêque, CHU de Bordeaux, 5 Avenue de Magellan, 33604 Pessac, France. Bordeaux Cardio-Thoracic Research Centre, U1045, Bordeaux University, Hôpital Xavier Arnozan, Avenue du Haut Lévêque, 33600 Pessac, France
3: Department of Interventional Cardiology, Cardiovascular Hospital and Claude-Bernard University, INSERM Unit 1060 CARMEN, Lyon, France.
4: Department of Cardiology, Hôpital Lariboisière, Assistance Publique des Hôpitaux de Paris, Paris, France.
5: Service de cardiologie, Les Hôpitaux de Chartres, Le Coudray, France
6: Besancon University Hospital, EA3920, University of Burgundy Franche-Comté, Besancon, France.
7: Service de cardiologie, hôpital Croix-Rousse et hôpital Lyon Sud, hospices civils de Lyon, Lyon, France; Université Lyon 1, CREATIS UMR5220, Inserm U1044, INSA-15, Lyon, France.
8: Department of Cardiology, Nimes University Hospital, Montpellier University, Nimes, France
9: Department of Cardiology, Intensive Coronary Care Unit, Emile Muller Hospital, Mulhouse, France.
10: Department of cardiology, centre hospitalier Annecy-Genevois, 74370 Metz-Tessy, France
11: Department of Cardiology, University Hospital of Rangueil, Toulouse, France
12: Department of Cardiology, Centre Hospitalier Départemental (CHD) de Vendée ,La Roche sur Yon, France.
13: Department of Cardiology, General Hospital of Atlantic Brittany, Vannes, France.
14: Department of Cardiology, Caen University Hospital, Caen, France.
15 : Department of cardiology, Centre Hospitalier de Versailles, Le Chesnay-Rocquencourt, France.
16 : Department of Cardiology, Centre Hospitalier Metropole Savoie, Chambery, France.
17 : Department of Cardiology, Grenoble University Hospital, Grenoble, France.

International Stroke Genetics Consortium (ISGC) Intracranial Aneurysm Working Group:

Mark K. Bakker
Department of Neurology and Neurosurgery, University Medical Center Utrecht Brain Center, Utrecht University, Utrecht, The Netherlands.
M.K.Bakker-25@umcutrecht.nl

Philippe Bijlenga
Neurosurgery Division, Department of Clinical Neurosciences, Faculty of Medicine, Geneva University Hospitals, Geneva, Switzerland.
Philippe.Bijlenga@hcuge.ch

Romain Bourcier
Université de Nantes, CHU Nantes, INSERM, CNRS, l'institut du thorax, Nantes, France.
CHU Nantes, Department of Neuroradiology, Nantes, France.
Romain.BOURCIER@chu-nantes.fr

Joseph P. Broderick
University of Cincinnati College of Medicine, Cincinnati, OH, USA.
BRODERJP@UCMAIL.UC.EDU

Mikael Fraunberg
Neurosurgery NeuroCenter, Kuopio University Hospital, Kuopio, Finland
Institute of Clinical Medicine, Faculty of Health Sciences, University of Eastern Finland, Kuopio, Finland.
Mikael.Fraunberg@kuh.fi

Emilia Gaal–Paavola
Department of Neurosurgery, Helsinki University Hospital, University of Helsinki, Helsinki, Finland and Clinical Neurosciences, University of Helsinki, Helsinki, Finland.
emilia.gaal-paavola@hus.fi

Isabel C. Hostettler
Department of Neurosurgery, Kantonspital St. Gallen, Rorschacher Strasse 95, 9007, St. Gallen, Switzerland.
isabel.hostettler@gmail.com

Juha E. Jaaskelainen
Neurosurgery NeuroCenter, Kuopio University Hospital, Kuopio, Finland. And Institute of Clinical Medicine, Faculty of Health Sciences, University of Eastern Finland, Kuopio, Finland.
Juha.E.Jaaskelainen@kuh.fi

Yoichiro Kamatani
Graduate School of Frontier Sciences, The University of Tokyo, Tokyo, Japan.
kamatani.yoichiro@gmail.com

Antti Lindgren
Neurosurgery NeuroCenter, Kuopio University Hospital, Kuopio, Finland.
Institute of Clinical Medicine, Faculty of Health Sciences, University of Eastern Finland, Kuopio, Finland.
Antti.Lindgren@kuh.fi

Sandrine Morel

Neurosurgery Division, Department of Clinical Neurosciences, Faculty of Medicine, Geneva University Hospitals, Geneva, Switzerland and Department of Pathology and Immunology, Faculty of Medicine, University of Geneva, Geneva, Switzerland.
sandrine.morel@hcuge.ch

Mika Niemela
Department of Neurosurgery, Helsinki University Hospital, University of Helsinki, Helsinki, Finland.
mika.niemela@hus.fi

Joanna Pera
Department of Neurology, Faculty of Medicine, Jagiellonian University Medical College, ul. Botaniczna 3, 31-503, Krakow, Poland.
pera@su.krakow.pl

Richard Redon
l'institut du thorax Université de Nantes, CHU Nantes, INSERM, CNRS, Nantes, France.
richard.redon@univ-nantes.fr

Gabriel J.E. Rinkel
Department of Neurology and Neurosurgery, University Medical Center Utrecht Brain Center, Utrecht University, Utrecht, The Netherlands.
g.j.e.rinkel@umcutrecht.nl

Guy A Rouleau
Montréal Neurological Institute and Hospital, McGill University, Montréal, QC, Canada.
guy.rouleau@mcgill.ca

Ynte M. Ruigrok
Department of Neurology and Neurosurgery, University Medical Center Utrecht Brain Center, Utrecht University, Utrecht, The Netherlands.
ij.m.ruigrok@umcutrecht.nl

Agnieszka Slowik
Department of Neurology, Faculty of Medicine, Jagiellonian University Medical College, ul. Botaniczna 3, 31-503, Krakow, Poland.
slowik@neuro.cm-uj.krakow.pl

Robin G. Walters
Clinical Trial Service Unit and Epidemiological Studies Unit, Nuffield Department of Population Health, University of Oxford, Oxford, U.K.
Medical Research Council Population Health Research Unit, University of Oxford, Oxford, U.K.
robin.walters@ndph.ox.ac.uk

David J. Werring
Stroke Research Centre, University College London Queen Square Institute of Neurology, London, UK.
d.werring@ucl.ac.uk

Bendik S Winsvold
Department of Research, Innovation and Education, Division of Clinical Neuroscience, Oslo University Hospital, Oslo, Norway.
K. G. Jebsen Center for Genetic Epidemiology, Department of Public Health and Nursing, Faculty of Medicine and Health Sciences, Norwegian University of Science and Technology, Trondheim, Norway.
bendik.s.winsvold@gmail.com

Daniel Woo
University of Cincinnati College of Medicine, Cincinnati, OH, USA.
WOODL@UCMAIL.UC.EDU

Bradford B. Worrall
Departments of Neurology and Public Health Sciences, University of Virginia School of Medicine, Charlottesville, VA, USA.
BBW9R@hscmail.mcc.virginia.edu

MEGASTROKE CONSORTIUM

Rainer Malik [1], Ganesh Chauhan [2], Matthew Traylor [3], Muralidharan Sargurupremraj [4,5], Yukinori Okada [6,7,8], Aniket Mishra [4,5], Loes Rutten-Jacobs [3], Anne-Katrin Giese [9], Sander W van der Laan [10], Solveig Gretarsdottir [11], Christopher D Anderson [12,13,14,14], Michael Chong [15], Hieab HH Adams [16,17], Tetsuro Ago [18], Peter Almgren [19], Philippe Amouyel [20,21], Hakan Ay [22,13], Traci M Bartz [23], Oscar R Benavente [24], Steve Bevan [25], Giorgio B Boncoraglio [26], Robert D Brown, Jr. [27], Adam S Butterworth [28,29], Caty Carrera [30,31], Cara L Carty [32,33], Daniel I Chasman [34,35], Wei-Min Chen [36], John W Cole [37], Adolfo Correa [38], Ioana Cotlarciuc [39], Carlos Cruchaga [40,41], John Danesh [28,42,43,44], Paul IW de Bakker [45,46], Anita L DeStefano [47,48], Marcel den Hoed [49], Qing Duan [50], Stefan T Engelter [51,52], Guido J Falcone [53,54], Rebecca F Gottesman [55], Raji P Grewal [56], Vilmundur Gudnason [57,58], Stefan Gustafsson [59], Jeffrey Haessler [60], Tamara B Harris [61], Ahamad Hassan [62], Aki S Havulinna [63,64], Susan R Heckbert [65], Elizabeth G Holliday [66,67], George Howard [68], Fang-Chi Hsu [69], Hyacinth I Hyacinth [70], M Arfan Ikram [16], Erik Ingelsson [71,72], Marguerite R Irvin [73], Xueqiu Jian [74], Jordi Jiménez-Conde [75], Julie A Johnson [76,77], J Wouter Jukema [78], Masahiro Kanai [6,7,79], Keith L Keene [80,81], Brett M Kissela [82], Dawn O Kleindorfer [82], Charles Kooperberg [60], Michiaki Kubo [83], Leslie A Lange [84], Carl D Langefeld [85], Claudia Langenberg [86], Lenore J Launer [87], Jin-Moo Lee [88], Robin Lemmens [89,90], Didier Leys [91], Cathryn M Lewis [92,93], Wei-Yu Lin [28,94], Arne G Lindgren [95,96], Erik Lorentzen [97], Patrik K Magnusson [98], Jane Maguire [99], Ani Manichaikul [36], Patrick F McArdle [100], James F Meschia [101], Braxton D Mitchell [100,102], Thomas H Mosley [103,104], Michael A Nalls [105,106], Toshiharu Ninomiya [107], Martin J O'Donnell [15,108], Bruce M Psaty [109,110,111,112], Sara L Pulit [113,45], Kristiina Rannikmäe [114,115], Alexander P Reiner [65,116], Kathryn M Rexrode [117], Kenneth Rice [118], Stephen S Rich [36], Paul M Ridker [34,35], Natalia S Rost [9,13], Peter M Rothwell [119], Jerome I Rotter [120,121], Tatjana Rundek [122], Ralph L Sacco [122], Saori Sakaue [7,123], Michele M Sale [124], Veikko Salomaa [63], Bishwa R Sapkota [125], Reinhold Schmidt [126], Carsten O Schmidt [127], Ulf Schminke [128], Pankaj Sharma [39], Agnieszka Slowik [129], Cathie LM Sudlow [114,115], Christian Tanislav [130], Turgut Tatlisumak [131,132], Kent D Taylor [120,121], Vincent NS Thijs [133,134], Gudmar Thorleifsson [11], Unnur Thorsteinsdottir [11], Steffen Tiedt [1], Stella Trompet [135], Christophe Tzourio [5,136,137], Cornelia M van Duijn [138,139], Matthew Walters [140], Nicholas J Wareham [86], Sylvia Wassertheil-Smoller [141], James G Wilson [142], Kerri L Wiggins [109], Qiong Yang [47], Salim Yusuf [15], Najaf Amin [16], Hugo S Aparicio [185,48], Donna K Arnett [186], John Attia [187], Alexa S Beiser [47,48], Claudine Berr [188], Julie E Buring [34,35], Mariana Bustamante [189], Valeria Caso [190], Yu-Ching Cheng [191], Seung Hoan Choi [192,48], Ayesha Chowhan [185,48], Natalia Cullell [31], Jean-François Dartigues [193,194], Hossein Delavaran [95,96], Pilar Delgado [195], Marcus Dörr [196,197], Gunnar Engström [19], Ian Ford [198], Wander S Gurpreet [199], Anders Hamsten [200,201], Laura Heitsch [202], Atsushi Hozawa [203], Laura Ibanez [204], Andreea Ilinca [95,96], Martin Ingelsson [205], Motoki Iwasaki [206], Rebecca D Jackson [207], Katarina Jood [208], Pekka Jousilahti [63], Sara Kaffashian [4,5], Lalit Kalra [209], Masahiro Kamouchi [210], Takanari Kitazono [211], Olafur Kjartansson [212], Manja Kloss [213], Peter J Koudstaal [214], Jerzy Krupinski [215], Daniel L Labovitz [216], Cathy C Laurie [118], Christopher R Levi [217], Linxin Li [218], Lars Lind [219], Cecilia M Lindgren [220,221], Vasileios Lioutas [222,48], Yong Mei Liu [223],

Oscar L Lopez [224], Hirata Makoto [225], Nicolas Martinez-Majander [172], Koichi Matsuda [225], Naoko Minegishi [203], Joan Montaner [226], Andrew P Morris [227,228], Elena Muiño [31], Martina Müller-Nurasyid [229,230,231], Bo Norrving [95,96], Soichi Ogishima [203], Eugenio A Parati [232], Leema Reddy Peddareddygari [56], Nancy L Pedersen [98,233], Joanna Pera [129], Markus Perola [63,234], Alessandro Pezzini [235], Silvana Pileggi [236], Raquel Rabionet [237], Iolanda Riba-Llena [30], Marta Ribasés [238], Jose R Romero [185,48], Jaume Roquer [239,240], Anthony G Rudd [241,242], Antti-Pekka Sarin [243,244], Ralhan Sarju [199], Chloe Sarnowski [47,48], Makoto Sasaki [245], Claudia L Satizabal [185,48], Mamoru Satoh [245], Naveed Sattar [246], Norie Sawada [206], Gerli Sibolt [172], Ásgeir Sigurdsson [247], Albert Smith [248], Kenji Sobue [245], Carolina Soriano-Tárraga [240], Tara Stanne [249], O Colin Stine [250], David J Stott [251], Konstantin Strauch [229,252], Takako Takai [203], Hideo Tanaka [253,254], Kozo Tanno [245], Alexander Teumer [255], Liisa Tomppo [172], Nuria P Torres-Aguila [31], Emmanuel Touze [256,257], Shoichiro Tsugane [206], Andre G Uitterlinden [258], Einar M Valdimarsson [259], Sven J van der Lee [16], Henry Völzke [255], Kenji Wakai [253], David Weir [260], Stephen R Williams [261], Charles DA Wolfe [241,242], Quenna Wong [118], Huichun Xu [191], Taiki Yamaji [206], Dharambir K Sanghera [125,169,170], Olle Melander [19], Christina Jern [171], Daniel Strbian [172,173], Israel Fernandez-Cadenas [31,30], W T Longstreth, Jr [174,65], Arndt Rolfs [175], Jun Hata [107], Daniel Woo [82], Jonathan Rosand [12,13,14], Guillaume Pare [15], Jemma C Hopewell [176], Danish Saleheen [177], Kari Stefansson [11,178], Bradford B Worrall [179], Steven J Kittner [37], Sudha Seshadri [180,48], Myriam Fornage [74,181], Hugh S Markus [3], Joanna MM Howson [28], Yoichiro Kamatani [6,182], Stephanie Debette [4,5], Martin Dichgans [1,183,184]

1 Institute for Stroke and Dementia Research (ISD), University Hospital, LMU Munich, Munich, Germany

2 Centre for Brain Research, Indian Institute of Science, Bangalore, India

3 Stroke Research Group, Division of Clinical Neurosciences, University of Cambridge, UK

4 INSERM U1219 Bordeaux Population Health Research Center, Bordeaux, France

5 University of Bordeaux, Bordeaux, France

6 Laboratory for Statistical Analysis, RIKEN Center for Integrative Medical Sciences, Yokohama, Japan

7 Department of Statistical Genetics, Osaka University Graduate School of Medicine, Osaka, Japan

8 Laboratory of Statistical Immunology, Immunology Frontier Research Center (WPI-IFReC), Osaka University, Suita, Japan.

9 Department of Neurology, Massachusetts General Hospital, Harvard Medical School, Boston, MA, USA

10 Laboratory of Experimental Cardiology, Division of Heart and Lungs, University Medical Center Utrecht, University of Utrecht, Utrecht,Netherlands

11 deCODE genetics/AMGEN inc, Reykjavik, Iceland

12 Center for Genomic Medicine, Massachusetts General Hospital (MGH), Boston, MA, USA

13 J. Philip Kistler Stroke Research Center, Department of Neurology, MGH, Boston, MA, USA

14 Program in Medical and Population Genetics, Broad Institute, Cambridge, MA, USA

15 Population Health Research Institute, McMaster University, Hamilton, Canada

16 Department of Epidemiology, Erasmus University Medical Center, Rotterdam, Netherlands
17 Department of Radiology and Nuclear Medicine, Erasmus University Medical Center, Rotterdam, Netherlands
18 Department of Medicine and Clinical Science, Graduate School of Medical Sciences, Kyushu University, Fukuoka, Japan
19 Department of Clinical Sciences, Lund University, Malmö, Sweden
20 Univ. Lille, Inserm, Institut Pasteur de Lille, LabEx DISTALZ-UMR1167, Risk factors and molecular determinants of aging-related diseases, F-59000 Lille, France
21 Centre Hosp. Univ Lille, Epidemiology and Public Health Department, F-59000 Lille, France
22 AA Martinos Center for Biomedical Imaging, Department of Radiology, Massachusetts General Hospital, Harvard Medical School, Boston, MA, USA
23 Cardiovascular Health Research Unit, Departments of Biostatistics and Medicine, University of Washington, Seattle, WA, USA
24 Division of Neurology, Faculty of Medicine, Brain Research Center, University of British Columbia, Vancouver, Canada
25 School of Life Science, University of Lincoln, Lincoln, UK
26 Department of Cerebrovascular Diseases, Fondazione IRCCS Istituto Neurologico "Carlo Besta", Milano, Italy
27 Department of Neurology, Mayo Clinic Rochester, Rochester, MN, USA
28 MRC/BHF Cardiovascular Epidemiology Unit, Department of Public Health and Primary Care, University of Cambridge, Cambridge, UK
29 The National Institute for Health Research Blood and Transplant Research Unit in Donor Health and Genomics, University of Cambridge, UK
30 Neurovascular Research Laboratory, Vall d'Hebron Institut of Research, Neurology and Medicine Departments-Universitat Autònoma de Barcelona, Vall d'Hebrón Hospital, Barcelona, Spain
31 Stroke Pharmacogenomics and Genetics, Fundacio Docència i Recerca MutuaTerrassa, Terrassa, Spain
32 Children's Research Institute, Children's National Medical Center, Washington, DC, USA
33 Center for Translational Science, George Washington University, Washington, DC, USA
34 Division of Preventive Medicine, Brigham and Women's Hospital, Boston, MA, USA
35 Harvard Medical School, Boston, MA, USA
36 Center for Public Health Genomics, Department of Public Health Sciences, University of Virginia, Charlottesville, VA, USA
37 Department of Neurology, University of Maryland School of Medicine and Baltimore VAMC, Baltimore, MD, USA
38 Departments of Medicine, Pediatrics and Population Health Science, University of Mississippi Medical Center, Jackson, MS, USA
39 Institute of Cardiovascular Research, Royal Holloway University of London, UK & Ashford and St Peters Hospital, Surrey UK

40 Department of Psychiatry,The Hope Center Program on Protein Aggregation and Neurodegeneration (HPAN),Washington University, School of Medicine, St. Louis, MO, USA
41 Department of Developmental Biology, Washington University School of Medicine, St. Louis, MO, USA
42 NIHR Blood and Transplant Research Unit in Donor Health and Genomics, Department of Public Health and Primary Care, University of Cambridge, Cambridge, UK
43 Wellcome Trust Sanger Institute, Wellcome Trust Genome Campus, Hinxton, Cambridge, UK
44 British Heart Foundation, Cambridge Centre of Excellence, Department of Medicine, University of Cambridge, Cambridge, UK
45 Department of Medical Genetics, University Medical Center Utrecht, Utrecht, Netherlands
46 Department of Epidemiology, Julius Center for Health Sciences and Primary Care, University Medical Center Utrecht, Utrecht, Netherlands
47 Boston University School of Public Health, Boston, MA, USA
48 Framingham Heart Study, Framingham, MA, USA
49 Department of Immunology, Genetics and Pathology and Science for Life Laboratory, Uppsala University, Uppsala, Sweden
50 Department of Genetics, University of North Carolina, Chapel Hill, NC, USA
51 Department of Neurology and Stroke Center, Basel University Hospital, Switzerland
52 Neurorehabilitation Unit, University and University Center for Medicine of Aging and Rehabilitation Basel, Felix Platter Hospital, Basel, Switzerland
53 Department of Neurology, Yale University School of Medicine, New Haven, CT, USA
54 Program in Medical and Population Genetics, The Broad Institute of Harvard and MIT, Cambridge, MA, USA
55 Department of Neurology, Johns Hopkins University School of Medicine, Baltimore, MD, USA
56 Neuroscience Institute, SF Medical Center, Trenton, NJ, USA
57 Icelandic Heart Association Research Institute, Kopavogur, Iceland
58 University of Iceland, Faculty of Medicine, Reykjavik, Iceland
59 Department of Medical Sciences, Molecular Epidemiology and Science for Life Laboratory, Uppsala University, Uppsala, Sweden
60 Division of Public Health Sciences, Fred Hutchinson Cancer Research Center, Seattle, WA, USA
61 Laboratory of Epidemiology and Population Science, National Institute on Aging, National Institutes of Health, Bethesda, MD, USA
62 Department of Neurology, Leeds General Infirmary, Leeds Teaching Hospitals NHS Trust, Leeds, UK
63 National Institute for Health and Welfare, Helsinki, Finland
64 FIMM - Institute for Molecular Medicine Finland, Helsinki, Finland
65 Department of Epidemiology, University of Washington, Seattle, WA, USA
66 Public Health Stream, Hunter Medical Research Institute, New Lambton, Australia
67 Faculty of Health and Medicine, University of Newcastle, Newcastle, Australia
68 School of Public Health, University of Alabama at Birmingham, Birmingham, AL, USA

69 Department of Biostatistical Sciences, Wake Forest School of Medicine, Winston-Salem, NC, USA
70 Aflac Cancer and Blood Disorder Center, Department of Pediatrics, Emory University School of Medicine, Atlanta, GA, USA
71 Department of Medicine, Division of Cardiovascular Medicine, Stanford University School of Medicine, CA, USA
72 Department of Medical Sciences, Molecular Epidemiology and Science for Life Laboratory, Uppsala University, Uppsala, Sweden
73 Epidemiology, School of Public Health, University of Alabama at Birmingham, USA
74 Brown Foundation Institute of Molecular Medicine, University of Texas Health Science Center at Houston, Houston, TX, USA
75 Neurovascular Research Group (NEUVAS), Neurology Department, Institut Hospital del Mar d'Investigació Mèdica, Universitat Autònoma de Barcelona, Barcelona, Spain
76 Department of Pharmacotherapy and Translational Research and Center for Pharmacogenomics, University of Florida, College of Pharmacy, Gainesville, FL, USA
77 Division of Cardiovascular Medicine, College of Medicine, University of Florida, Gainesville, FL, USA
78 Department of Cardiology, Leiden University Medical Center, Leiden, the Netherlands
79 Program in Bioinformatics and Integrative Genomics, Harvard Medical School, Boston, MA, USA
80 Department of Biology, East Carolina University, Greenville, NC, USA
81 Center for Health Disparities, East Carolina University, Greenville, NC, USA
82 University of Cincinnati College of Medicine, Cincinnati, OH, USA
83 RIKEN Center for Integrative Medical Sciences, Yokohama, Japan
84 Department of Medicine, University of Colorado Denver, Anschutz Medical Campus, Aurora, CO, USA
85 Center for Public Health Genomics and Department of Biostatistical Sciences, Wake Forest School of Medicine, Winston-Salem, NC, USA
86 MRC Epidemiology Unit, University of Cambridge School of Clinical Medicine, Institute of Metabolic Science, Cambridge Biomedical Campus, Cambridge, UK
87 Intramural Research Program, National Institute on Aging, National Institutes of Health, Bethesda, MD, USA
88 Department of Neurology, Radiology, and Biomedical Engineering, Washington University School of Medicine, St. Louis, MO, USA
89 KU Leuven – University of Leuven, Department of Neurosciences, Experimental Neurology, Leuven, Belgium
90 VIB Center for Brain & Disease Research, University Hospitals Leuven, Department of Neurology, Leuven, Belgium
91 Univ.-Lille, INSERM U 1171. CHU Lille. Lille, France
92 Department of Medical and Molecular Genetics, King's College London, London, UK

93 SGDP Centre, Institute of Psychiatry, Psychology & Neuroscience, King's College London, London, UK
94 Northern Institute for Cancer Research, Paul O'Gorman Building, Newcastle University, Newcastle, UK
95 Department of Clinical Sciences Lund, Neurology, Lund University, Lund, Sweden
96 Department of Neurology and Rehabilitation Medicine, Skåne University Hospital, Lund, Sweden
97 Bioinformatics Core Facility, University of Gothenburg, Gothenburg, Sweden
98 Department of Medical Epidemiology and Biostatistics, Karolinska Institutet, Stockholm, Sweden
99 University of Technology Sydney, Faculty of Health, Ultimo, Australia
100 Department of Medicine, University of Maryland School of Medicine, MD, USA
101 Department of Neurology, Mayo Clinic, Jacksonville, FL, USA
102 Geriatrics Research and Education Clinical Center, Baltimore Veterans Administration Medical Center, Baltimore, MD, USA
103 Division of Geriatrics, School of Medicine, University of Mississippi Medical Center, Jackson, MS, USA
104 Memory Impairment and Neurodegenerative Dementia Center, University of Mississippi Medical Center, Jackson, MS, USA
105 Laboratory of Neurogenetics, National Institute on Aging, National institutes of Health, Bethesda, MD, USA
106 Data Tecnica International, Glen Echo MD, USA
107 Department of Epidemiology and Public Health, Graduate School of Medical Sciences, Kyushu University, Fukuoka, Japan
108 Clinical Research Facility, Department of Medicine, NUI Galway, Galway, Ireland
109 Cardiovascular Health Research Unit, Department of Medicine, University of Washington, Seattle, WA, USA
110 Department of Epidemiology, University of Washington, Seattle, WA
111 Department of Health Services, University of Washington, Seattle, WA, USA
112 Kaiser Permanente Washington Health Research Institute, Seattle, WA, USA
113 Brain Center Rudolf Magnus, Department of Neurology, University Medical Center Utrecht, Utrecht, The Netherlands
114 Usher Institute of Population Health Sciences and Informatics, University of Edinburgh, Edinburgh, UK
115 Centre for Clinical Brain Sciences, University of Edinburgh, Edinburgh, UK
116 Fred Hutchinson Cancer Research Center, University of Washington, Seattle, WA, USA
117 Department of Medicine, Brigham and Women's Hospital, Boston, MA, USA
118 Department of Biostatistics, University of Washington, Seattle, WA, USA
119 Nuffield Department of Clinical Neurosciences, University of Oxford, UK

120 Institute for Translational Genomics and Population Sciences, Los Angeles Biomedical Research Institute at Harbor-UCLA Medical Center, Torrance, CA, USA
121 Division of Genomic Outcomes, Department of Pediatrics, Harbor-UCLA Medical Center, Torrance, CA, USA
122 Department of Neurology, Miller School of Medicine, University of Miami, Miami, FL, USA
123 Department of Allergy and Rheumatology, Graduate School of Medicine, the University of Tokyo, Tokyo, Japan
124 Center for Public Health Genomics, University of Virginia, Charlottesville, VA, USA
125 Department of Pediatrics, College of Medicine, University of Oklahoma Health Sciences Center, Oklahoma City, OK, USA
126 Department of Neurology, Medical University of Graz, Graz, Austria
127 University Medicine Greifswald, Institute for Community Medicine, SHIP-KEF, Greifswald, Germany
128 University Medicine Greifswald, Department of Neurology, Greifswald, Germany
129 Department of Neurology, Jagiellonian University, Krakow, Poland
130 Department of Neurology, Justus Liebig University, Giessen, Germany
131 Department of Clinical Neurosciences/Neurology, Institute of Neuroscience and Physiology, Sahlgrenska Academy at University of Gothenburg, Gothenburg, Sweden
132 Sahlgrenska University Hospital, Gothenburg, Sweden
133 Stroke Division, Florey Institute of Neuroscience and Mental Health, University of Melbourne, Heidelberg, Australia
134 Austin Health, Department of Neurology, Heidelberg, Australia
135 Department of Internal Medicine, Section Gerontology and Geriatrics, Leiden University Medical Center, Leiden, the Netherlands
136 INSERM U1219, Bordeaux, France
137 Department of Public Health, Bordeaux University Hospital, Bordeaux, France
138 Genetic Epidemiology Unit, Department of Epidemiology, Erasmus University Medical Center Rotterdam, Netherlands
139 Center for Medical Systems Biology, Leiden, Netherlands
140 School of Medicine, Dentistry and Nursing at the University of Glasgow, Glasgow, UK
141 Department of Epidemiology and Population Health, Albert Einstein College of Medicine, NY, USA
142 Department of Physiology and Biophysics, University of Mississippi Medical Center, Jackson, MS, USA
143 A full list of members and affiliations appears in the Supplementary Note
144 Department of Human Genetics, McGill University, Montreal, Canada
145 Department of Pathophysiology, Institute of Biomedicine and Translation Medicine, University of Tartu, Tartu, Estonia
146 Department of Cardiac Surgery, Tartu University Hospital, Tartu, Estonia
147 Clinical Gene Networks AB,Stockholm, Sweden

148 Department of Genetics and Genomic Sciences, The Icahn Institute for Genomics and Multiscale Biology Icahn School of Medicine at Mount Sinai, New York, NY , USA
149 Department of Pathophysiology, Institute of Biomedicine and Translation Medicine, University of Tartu, Biomeedikum, Tartu, Estonia
150 Integrated Cardio Metabolic Centre, Department of Medicine, Karolinska Institutet, Karolinska Universitetssjukhuset, Huddinge, Sweden.
151 Clinical Gene Networks AB, Stockholm, Sweden
152 Sorbonne Universités, UPMC Univ. Paris 06, INSERM, UMR_S 1166, Team Genomics & Pathophysiology of Cardiovascular Diseases, Paris, France
153 ICAN Institute for Cardiometabolism and Nutrition, Paris, France
154 Department of Biomedical Engineering, University of Virginia, Charlottesville, VA, USA
155 Group Health Research Institute, Group Health Cooperative, Seattle, WA, USA
156 Seattle Epidemiologic Research and Information Center, VA Office of Research and Development, Seattle, WA, USA
157 Cardiovascular Research Center, Massachusetts General Hospital, Boston, MA, USA
158 Department of Medical Research, Bærum Hospital, Vestre Viken Hospital Trust, Gjettum, Norway
159 Saw Swee Hock School of Public Health, National University of Singapore and National University Health System, Singapore
160 National Heart and Lung Institute, Imperial College London, London, UK
161 Department of Gene Diagnostics and Therapeutics, Research Institute, National Center for Global Health and Medicine, Tokyo, Japan
162 Department of Epidemiology, Tulane University School of Public Health and Tropical Medicine, New Orleans, LA, USA
163 Department of Cardiology,University Medical Center Groningen, University of Groningen, Netherlands
164 MRC-PHE Centre for Environment and Health, School of Public Health, Department of Epidemiology and Biostatistics, Imperial College London, London, UK
165 Department of Epidemiology and Biostatistics, Imperial College London, London, UK
166 Department of Cardiology, Ealing Hospital NHS Trust, Southall, UK
167 National Heart, Lung and Blood Research Institute, Division of Intramural Research, Population Sciences Branch, Framingham, MA, USA
168 A full list of members and affiliations appears at the end of the manuscript
169 Department of Phamaceutical Sciences, Collge of Pharmacy, University of Oklahoma Health Sciences Center, Oklahoma City, OK, USA
170 Oklahoma Center for Neuroscience, Oklahoma City, OK, USA
171 Department of Pathology and Genetics, Institute of Biomedicine, The Sahlgrenska Academy at University of Gothenburg, Gothenburg, Sweden
172 Department of Neurology, Helsinki University Hospital, Helsinki, Finland
173 Clinical Neurosciences, Neurology, University of Helsinki, Helsinki, Finland

174 Department of Neurology, University of Washington, Seattle, WA, USA
175 Albrecht Kossel Institute, University Clinic of Rostock, Rostock, Germany
176 Clinical Trial Service Unit and Epidemiological Studies Unit, Nuffield Department of Population Health, University of Oxford, Oxford, UK
177 Department of Genetics, Perelman School of Medicine, University of Pennsylvania, PA, USA
178 Faculty of Medicine, University of Iceland, Reykjavik, Iceland
179 Departments of Neurology and Public Health Sciences, University of Virginia School of Medicine, Charlottesville, VA, USA
180 Department of Neurology, Boston University School of Medicine, Boston, MA, USA
181 Human Genetics Center, University of Texas Health Science Center at Houston, Houston, TX, USA
182 Center for Genomic Medicine, Kyoto University Graduate School of Medicine, Kyoto, Japan
183 Munich Cluster for Systems Neurology (SyNergy), Munich, Germany
184 German Center for Neurodegenerative Diseases (DZNE), Munich, Germany
185 Boston University School of Medicine, Boston, MA, USA
186 University of Kentucky College of Public Health, Lexington, KY, USA
187 University of Newcastle and Hunter Medical Research Institute, New Lambton, Australia
188 Univ. Montpellier, Inserm, U1061, Montpellier, France
189 Centre for Research in Environmental Epidemiology, Barcelona, Spain
190 Department of Neurology, Università degli Studi di Perugia, Umbria, Italy
191 Department of Medicine, University of Maryland School of Medicine, Baltimore, MD, USA
192 Broad Institute, Cambridge, MA, USA
193 Univ. Bordeaux, Inserm, Bordeaux Population Health Research Center, UMR 1219, Bordeaux, France
194 Bordeaux University Hospital, Department of Neurology, Memory Clinic, Bordeaux, France
195 Neurovascular Research Laboratory. Vall d'Hebron Institut of Research, Neurology and Medicine Departments-Universitat Autònoma de Barcelona. Vall d’Hebrón Hospital, Barcelona, Spain
196 University Medicine Greifswald, Department of Internal Medicine B, Greifswald, Germany
197 DZHK, Greifswald, Germany
198 Robertson Center for Biostatistics, University of Glasgow, Glasgow, UK
199 Hero DMC Heart Institute, Dayanand Medical College & Hospital, Ludhiana, India
200 Atherosclerosis Research Unit, Department of Medicine Solna, Karolinska Institutet, Stockholm, Sweden
201 Karolinska Institutet, Stockholm, Sweden
202 Division of Emergency Medicine, and Department of Neurology, Washington University School of Medicine, St. Louis, MO, USA
203 Tohoku Medical Megabank Organization, Sendai, Japan
204 Department of Psychiatry, Washington University School of Medicine, St. Louis, MO, USA

205 Department of Public Health and Caring Sciences / Geriatrics, Uppsala University, Uppsala, Sweden
206 Epidemiology and Prevention Group, Center for Public Health Sciences, National Cancer Center, Tokyo, Japan
207 Department of Internal Medicine and the Center for Clinical and Translational Science, The Ohio State University, Columbus, OH, USA
208 Institute of Neuroscience and Physiology, the Sahlgrenska Academy at University of Gothenburg, Goteborg, Sweden
209 Department of Basic and Clinical Neurosciences, King's College London, London, UK
210 Department of Health Care Administration and Management, Graduate School of Medical Sciences, Kyushu University, Japan
211 Department of Medicine and Clinical Science, Graduate School of Medical Sciences, Kyushu University, Japan
212 Landspitali National University Hospital, Departments of Neurology & Radiology, Reykjavik, Iceland
213 Department of Neurology, Heidelberg University Hospital, Germany
214 Department of Neurology, Erasmus University Medical Center
215 Hospital Universitari Mutua Terrassa, Terrassa (Barcelona), Spain
216 Albert Einstein College of Medicine, Montefiore Medical Center, New York, NY, USA
217 John Hunter Hospital, Hunter Medical Research Institute and University of Newcastle, Newcastle, NSW, Australia
218 Centre for Prevention of Stroke and Dementia, Nuffield Department of Clinical Neurosciences, University of Oxford, UK
219 Department of Medical Sciences, Uppsala University, Uppsala, Sweden
220 Genetic and Genomic Epidemiology Unit, Wellcome Trust Centre for Human Genetics, University of Oxford, Oxford, UK
221 The Wellcome Trust Centre for Human Genetics, Oxford, UK
222 Beth Israel Deaconess Medical Center, Boston, MA, USA
223 Wake Forest School of Medicine, Wake Forest, NC, USA
224 Department of Neurology, University of Pittsburgh, Pittsburgh, PA, USA
225 BioBank Japan, Laboratory of Clinical Sequencing, Department of Computational biology and medical Sciences, Graduate school of Frontier Sciences, The University of Tokyo, Tokyo, Japan
226 Neurovascular Research Laboratory, Vall d'Hebron Institut of Research, Neurology and Medicine Departments-Universitat Autònoma de Barcelona. Vall d’Hebrón Hospital, Barcelona, Spain
227 Department of Biostatistics, University of Liverpool, Liverpool, UK
228 Wellcome Trust Centre for Human Genetics, University of Oxford, Oxford, UK
229 Institute of Genetic Epidemiology, Helmholtz Zentrum München - German Research Center for Environmental Health, Neuherberg, Germany
230 Department of Medicine I, Ludwig-Maximilians-Universität, Munich, Germany

231 DZHK (German Centre for Cardiovascular Research), partner site Munich Heart Alliance, Munich, Germany
232 Department of Cerebrovascular Diseases, Fondazione IRCCS Istituto Neurologico “Carlo Besta”, Milano, Italy
233 Karolinska Institutet, MEB, Stockholm, Sweden
234 University of Tartu, Estonian Genome Center, Tartu, Estonia, Tartu, Estonia
235 Department of Clinical and Experimental Sciences, Neurology Clinic, University of Brescia, Italy
236 Translational Genomics Unit, Department of Oncology, IRCCS Istituto di Ricerche Farmacologiche Mario Negri, Milano, Italy
237 Department of Genetics, Microbiology and Statistics, University of Barcelona, Barcelona, Spain
238 Psychiatric Genetics Unit, Group of Psychiatry, Mental Health and Addictions, Vall d’Hebron Research Institute (VHIR), Universitat Autònoma de Barcelona, Biomedical Network Research Centre on Mental Health (CIBERSAM), Barcelona, Spain
239 Department of Neurology, IMIM-Hospital del Mar, and Universitat Autònoma de Barcelona, Spain
240 IMIM (Hospital del Mar Medical Research Institute), Barcelona, Spain
241 National Institute for Health Research Comprehensive Biomedical Research Centre, Guy's & St. Thomas' NHS Foundation Trust and King's College London, London, UK
242 Division of Health and Social Care Research, King's College London, London, UK
243 FIMM-Institute for Molecular Medicine Finland, Helsinki, Finland
244 THL-National Institute for Health and Welfare, Helsinki, Finland
245 Iwate Tohoku Medical Megabank Organization, Iwate Medical University, Iwate, Japan
246 BHF Glasgow Cardiovascular Research Centre, Faculty of Medicine, Glasgow, UK
247 deCODE Genetics/Amgen, Inc., Reykjavik, Iceland
248 Icelandic Heart Association, Reykjavik, Iceland
249 Institute of Biomedicine, the Sahlgrenska Academy at University of Gothenburg, Goteborg, Sweden
250 Department of Epidemiology, University of Maryland School of Medicine, Baltimore, MD, USA
251 Institute of Cardiovascular and Medical Sciences, Faculty of Medicine, University of Glasgow, Glasgow, UK
252 Chair of Genetic Epidemiology, IBE, Faculty of Medicine, LMU Munich, Germany
253 Division of Epidemiology and Prevention, Aichi Cancer Center Research Institute, Nagoya, Japan
254 Department of Epidemiology, Nagoya University Graduate School of Medicine, Nagoya, Japan
255 University Medicine Greifswald, Institute for Community Medicine, SHIP-KEF, Greifswald, Germany
256 Department of Neurology, Caen University Hospital, Caen, France

257 University of Caen Normandy, Caen, France
258 Department of Internal Medicine, Erasmus University Medical Center, Rotterdam, Netherlands
259 Landspitali University Hospital, Reykjavik, Iceland
260 Survey Research Center, University of Michigan, Ann Arbor, MI, USA
261 University of Virginia Department of Neurology, Charlottesville, VA, USA

CARDIoGRAMplusC4D Consortium

Krishna G Aragam[1,2,3,4]*, Tao Jiang[5]*, Anuj Goel[6,7]*, Stavroula Kanoni[8]*, Brooke N Wolford[9]*, Elle M Weeks[4], Minxian Wang[3,4], George Hindy[10], Wei Zhou[4,11,12,9], Christopher Grace[6,7], Carolina Roselli[3], Nicholas A Marston[13], Frederick K Kamanu[13], Ida Surakka[14], Loreto Muñoz Venegas[15,16], Paul Sherliker[17], Satoshi Koyama[18], Kazuyoshi Ishigaki[19], Bjørn O Åsvold[20,21,22], Michael R Brown[23], Ben Brumpton[20,21], Paul S de Vries[23], Olga Giannakopoulou[8], Panagiota Giardoglou[24], Daniel F Gudbjartsson[25,26], Ulrich Güldener[27], Syed M. Ijlal Haider[15], Anna Helgadottir[25], Maysson Ibrahim[28], Adnan Kastrati[27,29], Thorsten Kessler[27,29], Ling Li[27], Lijiang Ma[30,31], Thomas Meitinger[32,33,29], Sören Mucha[15], Matthias Munz[15], Federico Murgia[28], Jonas B Nielsen[34,20], Markus M Nöthen[35], Shichao Pang[27], Tobias Reinberger[15], Gudmar Thorleifsson[25], Moritz von Scheidt[27,29], Jacob K Ulirsch[4,11,36], EPIC-CVD Consortium, Biobank Japan, David O Arnar[25,37,38], Deepak S Atri[39,3], Noël P Burtt[4], Maria C Costanzo[4], Jason Flannick[40], Rajat M Gupta[39,3,4], Kaoru Ito[18], Dong-Keun Jang[4], Yoichiro Kamatani[41], Amit V Khera[2,3,4], Issei Komuro[42], Iftikhar J Kullo[43], Luca A Lotta[44], Christopher P Nelson[45], Robert Roberts[46], Gudmundur Thorgeirsson[25,37,38], Unnur Thorsteinsdottir[25,37], Thomas R Webb[45], Aris Baras[44], Johan LM Björkegren[47,48,49], Eric Boerwinkle[23,50], George Dedoussis[24], Hilma Holm[25], Kristian Hveem[20,21], Olle Melander[51], Alanna C Morrison[23], Marju Orho-Melander[51], Loukianos S Rallidis[52], Arno Ruusalepp[53], Marc S Sabatine[13], Kari Stefansson[25,37], Pierre Zalloua[54,55], Patrick T Ellinor[1,3], Martin Farrall[6,7], John Danesh[5,56,57,58,59,60], Christian T Ruff[13], Hilary K Finucane[4,11,12], Jemma C Hopewell[28], Robert Clarke[28], Jeanette Erdmann[15,61†], Nilesh J Samani[45†], Heribert Schunkert[27,29†], Hugh Watkins[6,7†], Cristen J Willer[14,9,62†], Panos Deloukas[8,63†], Sekar Kathiresan[64†], Adam S Butterworth[5,56,60,59,57†] on behalf of.

[1]Cardiovascular Research Center, Massachusetts General Hospital, 185 Cambridge St., Boston, MA, 02114, USA

[2]Center for Genomic Medicine, Massachusetts General Hospital, 185 Cambridge St., Boston, MA, 02114, USA

[3]Cardiovascular Disease Initiative, Broad Institute of MIT and Harvard, 75 Ames St., Cambridge, MA, 02142, USA

[4]Program in Medical and Population Genetics, Broad Institute of MIT and Harvard, 75 Ames St., Cambridge, MA, 02142, USA

[5]BHF Cardiovascular Epidemiology Unit, Department of Public Health and Primary Care, University of Cambridge, Worts Causeway, Cambridge, CB1 8RN, UK

[6]Radcliffe Department of Medicine, Division of Cardiovascular Medicine, University of Oxford, Headley Way, Oxford, OX3 9DU, UK

[7]Wellcome Centre for Human Genetics, University of Oxford, Roosevelt Drive, Oxford, OX3 7BN, UK

[8]William Harvey Research Institute, Barts and the London School of Medicine and Dentistry, Queen Mary University of London, Charterhouse square, London, EC1M 6BQ, UK

[9]Department of Computational Medicine & Bioinformatics, University of Michigan, Palmer Ave., Ann Arbor, Michigan, 48109, USA

[10]Department of Population Medicine, Qatar University College of Medicine, Doha, Qatar

[11]Analytic and Translational Genetics Unit, Massachusetts General Hospital, Boston, Massachusetts, 02114, USA

[12]Stanley Center for Psychiatric Research, Broad Institute of MIT and Harvard, Cambridge, Massachusetts, USA

[13]TIMI Study Group, Division of Cardiovascular Medicine, Brigham and Women's Hospital, Harvard Medical School, 60 Fenwood Rd., Boston, Massachusetts, 02115, USA

[14]Department of Internal Medicine, Cardiology, University of Michigan, E. Catherine St., Ann Arbor, Michigan, 48109, USA

[15]Institute for Cardiogenetics, University of Lübeck, Lübeck, 23562, Germany [16]DZHK (German Research Center for Cardiovascular Research), partner site Hamburg-Lübeck-Kiel, Lübeck, Germany

[17]Medical Research Council Population Health Research Unit, CTSU - Nuffield Department of Population Health, Medical Sciences Division, University of Oxford, Roosevelt Drive, Oxford, OX3 7LF, UK

[18]Laboratory for Cardiovascular Genomics and Informatics, RIKEN Center for Integrative Medical Sciences, 1-7-22 Suehiro-cho, Tsurumi-ku, Yokohama, Kanagawa, 230-0045, Japan

[19]Laboratory for Statistical and Translational Genetics, RIKEN Center for Integrative Medical Sciences, 1- 7-22 Suehiro-cho, Tsurumi-ku, Yokohama, Kanagawa, 230-0045, Japan

[20]K.G. Jebsen Center for Genetic Epidemiology, Department of Public Health and Nursing, Norwegian University of Science and Technology, NTNU, Trondheim, Norway

[21]HUNT Research Centre, Norwegian University of Science and Technology, Levanger, Norway

[22]Department of Endocrinology, Clinic of Medicine, St. Olavs Hospital, Trondheim, Norway

[23]Human Genetics Center, Department of Epidemiology, Human Genetics, and Environmental Sciences, School of Public Health, The University of Texas Health Science Center at Houston, 1200 Pressler St., Houston, TX, 77030, USA

[24]School of Health Science & Education, Department of Nutrition-Dietetics, Harokopio University, Eleftheriou Venizelou 70, Athens, 176 71, Greece

[25]deCODE genetics/Amgen, Inc., Sturlugata 8, Reykjavik, 102, Iceland

[26]School of Engineering and Natural Sciences, University of Iceland, Sæmundargötu 2, Reykjavik, 102, Iceland

[27]German Heart Centre Munich, Department of Cardiology, Technical University of Munich, Lazarettstr. 36, Munich, 80636, Germany

[28]CTSU - Nuffield Department of Population Health, Medical Sciences Division, University of Oxford, Roosevelt Drive, Oxford, OX3 7LF, UK

[29]German Research Center for Cardiovascular Research (DZHK e.V.), partner site Munich Heart Alliance, Lazarettstr. 36, Munich, 80636, Germany

[30]Department of Genetics and Genomic Science, Icahn Institute for Genomics and Multiscale Biology, Icahn School of Medicine at Mount Sinai, New York, NY, 10029, USA

[31]The Zena and Michael A. Wiener Cardiovascular Institute, Icahn School of Medicine at Mount Sinai, New York, NY, 10029, USA

[32]Institute of Human Genetics, Helmholtz Zentrum München, German Research Center for Environmental Health, Neuherberg, Germany

[33]Klinikum rechts der Isar, Institute of Human Genetics, Technical University of Munich, Munich, Germany

[34]Department of Internal Medicine, Cardiology, University of Michigan, E. Catherine St., Ann Arbor, Michigan, 48109, US

[35]School of Medicine & University Hospital Bonn, Institute of Human Genetics, University of Bonn, Bonn, Germany

[36]Program in Biological and Biomedical Sciences, Harvard Medical School, Boston, Massachusetts, 02115, USA

[37]Faculty of Medicine, University of Iceland, Sæmundargötu 2, Reykjavik, 102, Iceland

[38]Department of Internal Medicine, Division of Cardiology, Landspitali – National University Hospital of Iceland, Hringbraut, Reykjavik, 101, Iceland

[39]Divisions of Cardiovascular Medicine and Genetics, Brigham and Women's Hospital, Harvard Medical School, 75 Francis St., Boston, MA, 02115, USA

[40]Division of Genetics and Genomics, Boston Children's Hospital, 300 Longwood Ave., Boston, MA, 02115, USA

[41]Department of Computational Biology and Medical Sciences, Graduate School of Frontier Sciences, The University of Tokyo, 4-6-1 Shirokanedai, Minato-ku, Tokyo, Tokyo, 108-8639, Japan

[42]Department of Cardiovascular Medicine, The University of Tokyo, 7-3-1 Hongo, Bunyko-ku, Tokyo, Tokyo, 113-8655, Japan

[43]Department of Cardiovascular Medicine, Mayo Clinic, MN, USA
[44]Regeneron Genetics Center, Regeneron Pharmaceuticals, 777 Old Saw Mill River Rd., Tarrytown, NY, 10591, USA
[45]Department of Cardiovascular Sciences and NIHR Leicester Biomedical Research Centre, University of Leicester, Glenfield Hospital, Groby Road, Leicester, LE3 9QP, UK
[46]Cardiovascular Genomics & Genetics, University of Arizona, College of Medicine, Phoenix, Arizona, USA
[47]Clinical Gene Networks AB, Stockholm, Sweden
[48]Department of Genetics & Genomic Sciences, Institute of Genomics and Multiscale Biology, Icahn School of Medicine at Mount Sinai, New York, USA
[49]Integrated Cardio Metabolic Centre, Karolinska Institutet, Karolinska Universitetssjukhuset, Huddinge, Sweden
[50]Human Genome Sequencing Center, Baylor College of Medicine, 1 Baylor Plaza, Houston, TX, 77030, USA
[51]Department of Clinical Sciences in Malmö, Lund University, Malmo, Sweden
[52]Second Department of Cardiology, Medical School, National and Kapodistrian University of Athens, University General Hospital Attikon, Athens, Greece
[53]Department of Cardiac Surgery and The Heart Clinic, Tartu University Hospital, Tartu, Estonia
[54]University of Balamand, East Med Res Institute, School of Medicine, P.O. Box 33, Amioun, Lebanon
[55]Harvard T.H.Chan School of Public Health, Boston, MA, USA
[56]Health Data Research UK Cambridge, Wellcome Genome Campus and University of Cambridge, Cambridge, UK
[57]The National Institute for Health Research Blood and Transplant Unit (NIHR BTRU) in Donor Health and Genomics at the University of Cambridge, Cambridge, UK
[58]Human Genetics, Wellcome Sanger Institute, Saffron Walden, UK
[59]National Institute for Health Research Cambridge Biomedical Research Centre, Cambridge University Hospitals, Cambridge, UK
[60]British Heart Foundation Centre of Excellence, Division of Cardiovascular Medicine, Addenbrooke's Hospital, Hills Road, Cambridge, UK
[61]DZHK (German Research Center for Cardiovascular Research), partner site Hamburg-Lübeck-Kiel, 23562, Germany
[62]Department of Human Genetics, University of Michigan, E. Catherine St., Ann Arbor, Michigan, 48109, USA
[63]Princess Al-Jawhara Al-Brahim Centre of Excellence in Research of Hereditary Disorders (PACER-HD), King Abdulaziz University, Jeddah, Saudi Arabia
[64]Verve Therapeutics, Cambridge, MA, 02139, USA
