# Supplementary material for: Genome-wide association meta-analysis of spontaneous coronary artery dissection identifies risk variants and genes related to artery integrity and tissue-mediated coagulation
Source: Nat Genet. 2023 May 29;55(6):964–72. doi: 10.1038/s41588-023-01410-1 (PMC10260398; doi:10.1038/s41588-023-01410-1)
Supplement: Supplementary file 2 — Reporting Summary [file 41588_2023_1410_MOESM2_ESM.pdf]

nature portfolio

Corresponding author(s): Nabila Bouatia-Naji
Last updated by author(s): Apr 17, 2023

Reporting Summary

Nature Portfolio wishes to improve the reproducibility of the work that we publish. This form provides structure for consistency and transparency in reporting. For further information on Nature Portfolio policies, see our Editorial Policies and the Editorial Policy Checklist.

Statistics

For all statistical analyses, confirm that the following items are present in the figure legend, table legend, main text, or Methods section.

n/a	Confirmed	
☐	☒	The exact sample size (*n*) for each experimental group/condition, given as a discrete number and unit of measurement
☒	☐	A statement on whether measurements were taken from distinct samples or whether the same sample was measured repeatedly
☐	☒	The statistical test(s) used AND whether they are one- or two-sided<br>*Only common tests should be described solely by name; describe more complex techniques in the Methods section.*
☐	☒	A description of all covariates tested
☐	☒	A description of any assumptions or corrections, such as tests of normality and adjustment for multiple comparisons
☐	☒	A full description of the statistical parameters including central tendency (e.g. means) or other basic estimates (e.g. regression coefficient) AND variation (e.g. standard deviation) or associated estimates of uncertainty (e.g. confidence intervals)
☐	☒	For null hypothesis testing, the test statistic (e.g. *F*, *t*, *r*) with confidence intervals, effect sizes, degrees of freedom and *P* value noted<br>*Give P values as exact values whenever suitable.*
☐	☒	For Bayesian analysis, information on the choice of priors and Markov chain Monte Carlo settings
☒	☐	For hierarchical and complex designs, identification of the appropriate level for tests and full reporting of outcomes
☒	☐	Estimates of effect sizes (e.g. Cohen's *d*, Pearson's *r*), indicating how they were calculated

Our web collection on statistics for biologists contains articles on many of the points above.

Software and code

Policy information about availability of computer code

Data collection

No software was used for data collection.

Data analysis

1. For genotyped cohorts, genotypes were imputed to HRC v1.1 reference panel on the Michigan Imputation Server.
2. GWAS was conducted in each study under an additive genetic model using PLINK v2.0.
3. Meta-analysis was performed using METAL software (March 2011 version).
4. LocusZoom 0.12.0 (http://locuszoom.org/) was used to provide regional visualization of results on April 14th 2022.
5. TWAS was performed using FUSION R/python package (version released 15th November 2021).
6. Statistical analyses and plotting were performed using R (4.1.0), through RStudio interface software (v1.2.335). Following packages were used: colorspace_2.0-3, ggnewscale_0.4.7, corrcoverage_1.2.1, disgenet2r_0.99.2, locuscomparer_1.0.0, coloc_5.1.0, dplyr_1.0.8, tidyr_1.2.0, ggrepel_0.9.1, RColorBrewer_1.1-3, shades_1.4.0, rtracklayer_1.52.1, LDlinkR_1.1.2, ggplot2_3.3.5, GenomicRanges_1.44.0, GenomeInfoDb_1.28.4, IRanges_2.26.0, S4Vectors_0.30.2, BiocGenerics_0.38.0, readr_2.1.2.
7. bedtools (v2.29.0) was used to handle bed files, merge peak files from the same tissue, generate bedGraph files for visualization of snATAC-Seq datasets.
8. snATAC-Seq peaks were detected using MACS2 bdgpeakcall function (Galaxy Version 2.1.1.20160309.0) on Galaxy webserver (https://usegalaxy.org/).
9. We used Integrated Genome Browser (IGB, v9.1.8) to visualize read density profiles.
10. SNP enrichment was calculated using GREGOR package (v1.4.0)
11. Heritability estimates were generated using LD score regression (LDSC) implemented in the ldsc package (v1.0.1, https://github.com/bulik/ldsc/) and SumHer implemented in LDAK software (version 5.2 , www.ldak.org).
12. Conditioning was performed using multi-trait-based conditional and joint analysis (mtCOJO) tool from GCTA pipeline (v1.94.1, https://yanglab.westlake.edu.cn/software/gcta/).

13. TwoSampleMR (v0.5.6) R package was used for Mendelian randomisation (MR) analyses.
14. RIMBANET (version 26th June 2019, https://labs.icahn.mssm.edu/zhulab/?s=rimbanet) and Enrichr webserver (Version of 29th March 2021, https://maayanlab.cloud/Enrichr/) were used for Bayesian network queries for prioritized genes.
15. The code for the druggability analysis can be found in GitLab (https://cfinan.gitlab.io/biomisc/scripts/drug_lookups.html).

For manuscripts utilizing custom algorithms or software that are central to the research but not yet described in published literature, software must be made available to editors and reviewers. We strongly encourage code deposition in a community repository (e.g. GitHub). See the Nature Portfolio guidelines for submitting code & software for further information.

Data

Policy information about availability of data

All manuscripts must include a data availability statement. This statement should provide the following information, where applicable:

- Accession codes, unique identifiers, or web links for publicly available datasets
- A description of any restrictions on data availability
- For clinical datasets or third party data, please ensure that the statement adheres to our policy

1. Gene reference names and coordinates were retrieved from GENCODE project through EBI FTP server. gencode.v38.annotation.gff3 and gencode.v38lift37.annotation.gff3 files were used.
2. eQTL data was retrieved from the Genotype Tissue Expression (GTEx) version 8 (https://gtexportal.org/home/datasets).
3. H3K27ac ChIP-Seq datasets (narrowpeaks beds) in any tissue were retrieved from ENCODE (https://www.encodeproject.org/).
4. Single nuclei ATAC-Seq peak files (bed format) from Human enhancer atlas (http://catlas.org/humanenhancer).
5. Open chromatin regions in healthy coronary arteries were generated from raw reads retrieved from Sequence Read Archive (SRR2378591, SRR2378592, SRR2378593).
6. Raw snATAC-Seq data in 25 adult tissues was retrieved from Gene Expression Omnibus (GSE184462).
7. Gene expression models for TWAS were retrieved from gusev lab website (http://gusevlab.org/projects/fusion/), based on GTEx data (v8 release).
8. Gene expression data from aorta artery, coronary artery, tibial artery, and cultured fibroblast was curated from the Genotype Tissue Expression (GTEx) version 8 (www.gtexportal.org/home/datasets).
9. Gene expression data from the mouse aorta was curated from the Hybrid Mouse Diversity Panel (HMDP).
10. Genes associated to mouse cardiovascular phenotypes (code MP:0005385) were retrieved from Mouse Genome informatics (www.informatics.jax.org).
11. Summary statistics were retrieved from http://www.cardiogramplusc4d.org/data-downloads/, http://ftp.ebi.ac.uk/pub/databases/gwas/summary_statistics/, https://www.megastroke.org/, http://www.nealelab.is/uk-biobank , https://diagram-consortium.org/downloads.html, or retrieved from authors as detailed in Supplementary table 17.
12. The set of genes encoding druggable targets was derived using ChEMBL v17, and further analyzed using ChEMBL v30 and the British National Formulary (BNF) (accessed 09/04/2021).
13. Summary statistics for SCAD association are available in GWAS catalog (GCP000522).

Human research participants

Policy information about studies involving human research participants and Sex and Gender in Research.

Reporting on sex and gender

Any findings in the present study only apply to sex and no indication about gender was collected and/or analyzed. Initial information about sex was collected by clinicians and the consistence with genotyping was assessed at the quality control step. Five samples were excluded due to a discrepancy between the clinical information and sex determined by genetic analysis. Sex stratified analyses were ran whenever possible. However, the low proportion of males in SCAD cohorts did not allow to perform a GWAS meta-analysis on males only.

Population characteristics

1. DISCO, (France), Total (n): 313, Women (n,%): 285 (91), Age at SCAD (Median, Q1/Q3): 52.2, 44.55, 60, Age at study
2. inclusion (Median, Q1,Q3): 51, 44, 59, FMD (Yes, No, NA): 140, 152, 21.
3. 3C-Study, (France), Total (n): 1487, Women (n,%): 876 (58.9), Age at SCAD (Median, Q1/Q3): NR, 51, 44, 59: 74.36 ± 5.5
4. [65 - 94], 140, 152, 21: NR.
5. SCAD-UK Study I - Cases, (UK), Total (n): 383, Women (n,%): 361 (94.2), Age at SCAD (Median, Q1/Q3): 47, 41, 52, 74.36 ± 5.5
6. [65 - 94]: NA, NR: 104,108,171.
7. SCAD-UK Study I - Controls, (UK), Total (n): 1925, Women (n,%): 1815 (94.3), Age at SCAD (Median, Q1/Q3): NR, NA: 56,49,62, 104,108,171: .
8. SCAD-UK Study II - Cases, (UK), Total (n): 139, Women (n,%): 115 (82.7), Age at SCAD (Median, Q1/Q3): 49.0, 43, 54, 56,49,62: NA, : 20,71,48.
9. SCAD-UK Study II - Controls, (UK), Total (n): 815, Women (n,%): 665 (81.6), Age at SCAD (Median, Q1/Q3): NR, NA: 56, 48, 61, 20,71,48: .
10. Mayo Clinic Study - Cases, (US), Total (n): 506, Women (n,%): 484, Age at SCAD (Median, Q1/Q3): 46.6, 39, 53, 56, 48, 61: 46.6 ± 9.2, : 175, 140, 169.
11. Mayo Clinic Study - Controls, (US), Total (n): 1549, Women (n,%): 1477, Age at SCAD (Median, Q1/Q3): NR, 46.6 ± 9.2: 64 ± 14.5, 175, 140, 169: unknown.
12. CanSCAD/MGI Study - Cases, (Canada/US), Total (n): 357, Women (n,%): 315 (88.2%), Age at SCAD (Median, Q1/Q3): , 64 ± 14.5: 53, 46, 60, unknown: 149,123,85.
13. CanSCAD/MGI Study - Controls, (Canada/US), Total (n): 2125, Women (n,%): 1873 (88.1%), Age at SCAD (Median, Q1/Q3): NR, 53, 46, 60: 53, 46, 61, 149,123,85: NR.
14. DEFINE-SCAD Study - Cases, (US), Total (n): 42, Women (n,%): 41 (97.6%), Age at SCAD (Median, Q1/Q3): 45.5, 36, 50.25
15. 6 missing values, 53, 46, 61: 49, 41.5, 53.75, NR: 31, 10, 1.
16. DEFINE-SCAD Study - Controls, (US), Total (n): 153, Women (n,%): 153 (100%), Age at SCAD (Median, Q1/Q3): NA, 49, 41.5, 53.75: 50 (43-58), 31, 10, 1: NR.

17. VCCRI Study I - Cases, (Australia), Total (n): 88, Women (n,%): 80, 90.9%, Age at SCAD (Median, Q1/Q3): 44, 39, 52, 50 (43-58): 50, 44, 59, NR: 14, 32, 42.
18. VCCRI Study I - Controls, (Australia), Total (n): 1127, Women (n,%): 672, 59.6%, Age at SCAD (Median, Q1/Q3): NA, 50, 44, 59: all >70 years old, 14, 32, 42: NR.
19. VCCRI Study II - Cases, (Australia), Total (n): 85, Women (n,%): 83, 97.6%, Age at SCAD (Median, Q1/Q3): 49, 43, 56, all >70 years old: 52, 48, 60, NR: 10, 22, 53.
20. VCCRI Study II - Controls, (Australia), Total (n): 111, Women (n,%): 46, 41.4%, Age at SCAD (Median, Q1/Q3): NA, 52, 48, 60: 61, 52, 67, 10, 22, 53: NR.

Recruitment

Altogether, the meta-analysis included participants of European ancestry from eight studies: DISCO-3C, SCAD-UK I, SCAD-UK II, Mayo Clinic, DEFINE-SCAD, CanSCAD/MGI, VCCRI I and VCCRI II. SCAD patients presented similar clinical characteristics and homogeneous diagnosis, exclusion and inclusion criteria. Controls were selected from local population-based studies or clinical studies led in the same centers. In the second case studies, SCAD or related vascular diseases were exclusion criteria. The rare presence of males in SCAD cohorts may be partly due to a lack of diagnosis in this population, considering that women are considered to be more at risk of SCAD. The limited presence of non-European cases and controls, likely related at least in part to socio-economic factors, prevents the analysis of these populations.
1. DISCO, (France).
1) Method of recruitment: Clinical based.
2) Inclusion criteria: age> 18, retrospective with a diagnostic of SCAD made from 2010, or prospective at the time of hospitalization during which the diagnosis of SCAD was made.
3) Exclusion criteria: Age <18; atherosclerotic ischemic disease; iatrogenic hematoma.
2. 3C-Study, (France).
1) Method of recruitment: Population based.
2) Inclusion criteria: Geographic sampling.
3) Exclusion criteria: Age < 65y.
3. SCAD-UK Study I - Cases, (UK).
1) Method of recruitment: Clinical based.
2) Inclusion criteria: SCAD confirmed on invasive angiography.
3) Exclusion criteria: Atherosclerotic dissection, iatrogenic dissection.
4. SCAD-UK Study I - Controls, (UK).
1) Method of recruitment: Population based.
2) Inclusion criteria: None.
3) Exclusion criteria: None.
5. SCAD-UK Study II - Cases, (UK).
1) Method of recruitment: Clinical based.
2) Inclusion criteria: SCAD confirmed on invasive angiography.
3) Exclusion criteria: Atherosclerotic dissection, iatrogenic dissection.
6. SCAD-UK Study II - Controls, (UK).
1) Method of recruitment: Population based.
2) Inclusion criteria: None.
3) Exclusion criteria: None.
7. Mayo Clinic Study - Cases, (US).
1) Method of recruitment: Clinical based.
2) Inclusion criteria: SCAD confirmed by angiogram.
3) Exclusion criteria: Diagnosis of connective tissue disorder or aortopathy; iatrogenic.
8. Mayo Clinic Study - Controls, (US).
1) Method of recruitment: Healthy volunteers.
2) Inclusion criteria: No reported SCAD.
3) Exclusion criteria: Diagnosis of atherosclerotic coronary artery disease, acute myocardial infarction, FMD, arterial aneurysm or dissection, cerebral infarction, Marfan syndrome, Ehlers-Danlos syndrome.
9. CanSCAD/MGI Study - Cases, (Canada/US).
1) Method of recruitment: Clinical based.
2) Inclusion criteria: SCAD diagnosis was confirmed on coronary angiography by the UBC core laboratory research team, and categorized according to previously established Saw classification.
3) Exclusion criteria: Angiogram unavailable or did not appear to be SCAD; from N=502, only Canadian samples consistent with 1000G non-Finish European ancestry (+/- 6 SD of PC1 and PC2) were retained for analysis. .
10. CanSCAD/MGI Study - Controls, (Canada/US).
1) Method of recruitment: Population based.
2) Inclusion criteria: Age, Sex, PC (PC1-PC3) matched controls.
3) Exclusion criteria: Of 13,756 MGI samples eligible for the study after exclusion of vascular or connective tissue diagnoses, and matching for age, sex and ancestry (based upon genetic PC's) 2,125 matched MGI controls were retained for analysis.
11. DEFINE-SCAD Study - Cases, (US).
1) Method of recruitment: Clinical based.
2) Inclusion criteria: SCAD confirmed on invasive angiography.
3) Exclusion criteria: Age < 18, diagnosis of connective tissue disorder or aortopathy; iatrogenic. Any diagnosis of other major diseases.
12. DEFINE-SCAD Study - Controls, (US).
1) Method of recruitment: Clinical based.
2) Inclusion criteria: Vascular disease excluded on history and physical exam. Also matched to SCAD cases by age, BMI, sex.
3) Exclusion criteria: Any diagnosis of vascular disease and other major diseases.
13. VCCRI Study I - Cases, (Australia)
1) Method of recruitment: Clinical based.
2) Inclusion criteria: SCAD confirmed by angiogram.
3) Exclusion criteria: Angiogram unavailable or did not appear to be SCAD.

14. VCCRI Study I - Controls, (Australia).
1) Method of recruitment: Population based.
2) Inclusion criteria: No reported SCAD.
3) Exclusion criteria: No reported history of cancer, cardiovascular disease or neurodegenerative diseases before 70 years old.
15. VCCRI Study II - Cases, (Australia).
1) Method of recruitment: Clinical based.
2) Inclusion criteria: SCAD confirmed by angiogram.
3) Exclusion criteria: Angiogram unavailable or did not appear to be SCAD.
16. VCCRI Study II - Controls, (Australia).
1) Method of recruitment: Clinical Based.
2) Inclusion criteria: No Reported SCAD.
3) Exclusion criteria: Related to other sample.

Ethics oversight

1. DISCO, (France): Clinical Trials ID: NCT02799186, regional committee CPP (comité de protection des personnes) Sud-Est 6 2016 AU-1258
2. 3C-Study, (France): «comité consultatif de protection des personnes dans la recherche biomédicale» Bicêtre Hôpital Bicêtre n°99-28 CCPPRB approved 10/06/99, 11/03/2003 and 17/03/2006.
3. SCAD-UK Study (UK): The UK SCAD study (ISRCTN42661582) was approved by the UK National Research Ethics Service (14/EM/0056) and the UK Health Research Authority.
4. Mayo Clinic Study (US): Mayo Clinic Institutional Review Board (NCT01429727; NCT01427179).
5. CanSCAD/MGI Study (Canada/US): Research ethics board approvals were obtained at each site of SCAD patient inclusion, and all patients provided informed consent for participation. IRB approval: HUM00113268, SCAD Registry and Research. IRB approval: HUM00112101, genetic analysis of arterial dysplasia and remodeling (MGI/AOS)
6. DEFINE-SCAD Study - Cases, (US): DEFINE study was approved by the Human Research Ethics Committee of the Icahn School of Medicine at Mount Sinai (Study ID: HS#13-00575/GCO#13–1118 and is registered with ClinicalTrials.gov Identifier: NCT01967511.
7. VCCRI Study I, (Australia): St. Vincent's Hospital Human Research Ethics Committee (HREC/16/SVH/338, protocol number SVH 16/245)
8. VCCRI Study II - Cases, (Australia): St. Vincent's Hospital Human Research Ethics Committee (HREC/17/SVH/315)

Note that full information on the approval of the study protocol must also be provided in the manuscript.

Field-specific reporting

Please select the one below that is the best fit for your research. If you are not sure, read the appropriate sections before making your selection.

☒ Life sciences ☐ Behavioural & social sciences ☐ Ecological, evolutionary & environmental sciences

For a reference copy of the document with all sections, see nature.com/documents/nr-reporting-summary-flat.pdf

Life sciences study design

All studies must disclose on these points even when the disclosure is negative.

Sample size

Recruitment of SCAD patients was the main limiting factor for sample size of GWAS case-control studies and by far represents the more thorough genetic investigation in SCAD. In 7 out of 8 case-control studies, the number of controls was picked to have a minimum 2.5x ratio over SCAD cases, in order to allow a robust measure of variant frequency, and maximum ratio of 15x the number of SCAD cases, to avoid artifical inflation of effective sample size. Number of controls in VCCRI arm II study was more limited due to constraints of clinical recruitment, but was set to outnumber SCAD patients in the study.

Data exclusions

1. Several datasets were excluded on the basis of classical quality control criteria. The detail of excluded datasets per study and per criterion is given in Supplementary table 13 and in the Methods and Supplementary Methods sections. In brief, 49 samples were excluded from CanSCAD study because they wer involved in another case-control study, 39 samples were excluded on the basis of heterozigocity/call rate assessment, 10 samples were excluded because of a high relatedness with other samples in the same study, 219 samples were excluded based on corrected diagnosis (non-SCAD), 2 samples were excluded due to a diagnosis of another genetic syndrome and 5 samples were excluded because sex determined by genotyping or sequencing did not match clinical data. All these criteria were included in study design. In addition, the analysis was restricted to European samples based on principal component analysis, as the number of non-European samples in control cohorts was insufficient.

2. Prior to meta-analysis, we removed single nucleotide polymorphisms (SNPs) with low minor allele frequencies (MAF<0.01), low imputation quality (r2 < 0.8), and deviations from Hardy-Weinberg equilibrium (P<10−5).

Replication

Here we only considered as risk loci for SCAD those that provided several associated SNPs with SCAD consistently across the 8 case control studies, with the same direction of effects, and no evidence for heterogeneity between studies We consider these criteria as evidence of replication accross studies, given that we used data from 6 independant recruitment centers from 5 countries. To maximise power, we explored results from the meta-analysis. Details about the association of each genome-wide associated locus are presented in Suppelementary table 2

Randomization

Individuals were allocated to patients and control groups through clinical recruitment and could not be randomized. To adjust for population

Randomization	covariates, genetic models were adjusted for population structure using the first five principal components, sex (except in the women-only analyses) and study specific genomic control.
Blinding	The investigators could not be blinded during data collection and analysis, as case and control cohorts originated from different protocols and/or required a thorough clinical investigation.

Reporting for specific materials, systems and methods

We require information from authors about some types of materials, experimental systems and methods used in many studies. Here, indicate whether each material, system or method listed is relevant to your study. If you are not sure if a list item applies to your research, read the appropriate section before selecting a response.

Materials & experimental systems

n/a	Involved in the study
☒	☐ Antibodies
☒	☐ Eukaryotic cell lines
☒	☐ Palaeontology and archaeology
☒	☐ Animals and other organisms
☒	☐ Clinical data
☒	☐ Dual use research of concern

Methods

n/a	Involved in the study
☒	☐ ChIP-seq
☒	☐ Flow cytometry
☒	☐ MRI-based neuroimaging
